# Improving Education and Training of Dutch Major Hazard Control Inspectors: A 15 Years Longitudinal Case Study

**DOI:** 10.3390/ijerph17061959

**Published:** 2020-03-17

**Authors:** Paul Lindhout, Karel van der Werff, Genserik L. L. M. E. Reniers

**Affiliations:** 1 Delft University of Technology, TPM Safety & Security Science Group, 2628 BX Delft, The Netherlands; G.L.L.M.E.Reniers@tudelft.nl; 2 Ministry of Social Affairs and Employment, Interdepartmental LOP-team, 2511 VX The Hague, The Netherlands; Kvdwerff@InspectieSZW.nl; 3 ARGoSS, Faculty of Applied Economics, University of Antwerp, 2000 Antwerp, Belgium; 4 CEDON, Campus Brussels, Faculty of Economics and Management, KU Leuven, 1000 Brussels, Belgium

**Keywords:** regulator, inspector education and training, major accident hazard establishment, Seveso III directive

## Abstract

The education and training program for inspectors of Major Accident Hazard Establishments, specifically the EC Seveso III directive implicated Dutch chemical companies, changed considerably over a fifteen year period. This longitudinal, time-series cross sectional case study describes the development of the education and training program for Major Hazard Control inspectors, acting as regulators from the Labour inspectorate, belonging to the Dutch Ministry of Social Affairs and Employment. A blueprint had to be constructed in order to assess the development and quality of this program in four cross sections over time. The description highlights both the safety related content and the regulator skills parts of the program in its changing context. Professional standards, educational objectives, quality of education, evaluation method, education change process and the response to the dynamic operational environment were examined. The main findings are that the education and training program kept the same main structure over the time period while its contents were adapted to respond to external context changes. Internal evaluation of performance data and education style led to a shift in contents from theoretical knowledge towards safety management and inspection practice oriented experience related knowledge. An active teaching style, increased usage of professional standards and more systematic evaluation, starting from the blue print in this study, offer the best opportunities for further improvement. Current insights on regulatory performance lead to a recommended future perspective for the inspectors’ role to be translated into education and training: balancing empathy, inquisitiveness and support with control and enforcement, or rather: *exert tough love*, staying between *too soft* and *too hard*.

“*It is time to accept that the harsh confrontational practices of the past are generally ineffective, potentially harmful, and professionally inappropriate.*”

*Quote: White & Miller, 2007* [1]

## 1. Introduction

In the Netherlands, Major Accident Hazard Establishments (MAHE), mainly situated in the chemical industry and using large quantities of hazardous substances, are inspected by regulator inspectors from several governmental inspectorates. 

The European Community (EC) Seveso III directive [2] applies to such companies. The directive is implemented in Dutch legislation by means of the Besluit Risico’s Zware Ongevallen (BRZO) [3] and a ministerial policy [4]. Several revisions since the first BRZO 1999 version have been implemented in The Netherlands, following successive changes in related standards and directives in the European Union. 

The BRZO implementation is connected to three laws, resulting in a cooperative inspection activity between three government institutions, the BRZO inspection partners: 

(A) The Labour Inspectorate, belonging to the Dutch Ministry of Social Affairs and Employment (MinSZW), was given a key role with the strongest law enforcement tools and its Major Hazard Control (MHC) inspectors, located within the Inspection of MinSZW (ISZW) as they are provided with an extensive technical and managerial knowledge base to be able to assess preventive safety performance in MAHE indicated companies. MinSZW operates within the legal framework of Labour legislation [5], Administrative law [6] for law enforcement and Fine policy [7]. Inspectors doing accident investigation, act within Criminal law [8,9].

(B) The local and regional inspection partners taking care of the environmental performance aspect were given a two sided and leading role. One side is the initiator and organizer of inspections and the other is holding the responsibility for the issue of permits and the license to operate to companies. These partners operate in the Environmental legislation framework [10]. 

(C) The fire brigade’s regional safety organizations became the main authority for the repression and emergency preparedness parts of safety and all emergency and disaster related activities. They operate within the bounds of the Safety regions and disaster mitigation law [11].

Since its first introduction, shortly after the 1999 version of BRZO [12] came into force, the Education and Training Program (ETP) for newly recruited Dutch (MHC) inspectors changed considerably over time. The changes concern both the role of inspectors in the BRZO field and the contents of the ETP. Parts of the ETP were discarded, changed or newly added in response to changing context, new insights and criticism on regulator performance. Managing such changes is a process taking place within the cooperation among government institutions, all being subject to political control on local, regional or national levels. The outcome of government activities regarding MAHE company safety performance has been subject of several evaluations and field studies, either associated with industrial accidents or with the implementation of new legislation. Information is being gathered and published online by the joint cooperation platform “BRZO+” [13]. 

Although the MHC inspector performance in the field has been subject of MinSZW customer satisfaction polls, the ETP itself has only been subject of internal MinSZW review. No scientific—nor third party—external evaluation of the Dutch MHC inspectors ETP content and quality has been conducted. The authors contend that such a scientific evaluation is important to support choices in future development. Thus far, the evolution of the ETP was influenced by many factors and many stakeholders during the development from 1999 onwards. Inspired by Niskanen et al. [14], pointing at an opportunity for longitudinal research in occupational health and safety, this study was conducted by three authors joining their expertise with education and training of regulator MHC inspectors, built-up over some 15 years. Thanks to their involvement from different points of view, this study provides rare insight into how MinSZW as a government organization, safeguards, adapts and improves the quality of their MHC regulator inspector ETP in response to the changing operational environment. This study deals with the following research question:

What can be said about the quality of the Dutch MHC inspector’s education and training program over the 2003–2018 time period?

## 2. Materials and Methods 

### 2.1. Design

Describing the development of education and training of regulator inspectors over a 15 year period can best be done using a longitudinal case study method according to Yin [15]. The setting up this case study was done according to a simplified version of the generally applicable steps of Eisenhardt [16]. Critical changes over time can be identified using a time series cross sectional case study design. This study uses 4 time sections, each structured in the same way with the help of a “blue print” template. The template contains a number of aspects that characterize the subject matter, its context, in this case the operational environment [17] and a number of education and training quality aspects as derived from a theoretical reference framework constructed in this study. Applying several data gathering methods (data triangulation) is safeguarding quality and validity [18]. Thematic synthesis is used to analyze the internal MinSZW source documents as received [19]. The internal validity is ensured by a comparison of literature findings with survey response results [20].

### 2.2. Researcher Bias

The researcher involvement in the subject matter introduces a potential research quality issue. To protect the study against bias by changes in the perspective of the authors, this was also taken up as a separate aspect in the template [21].

### 2.3. Selection of Time Period and Time Sections

**Figure 1:**
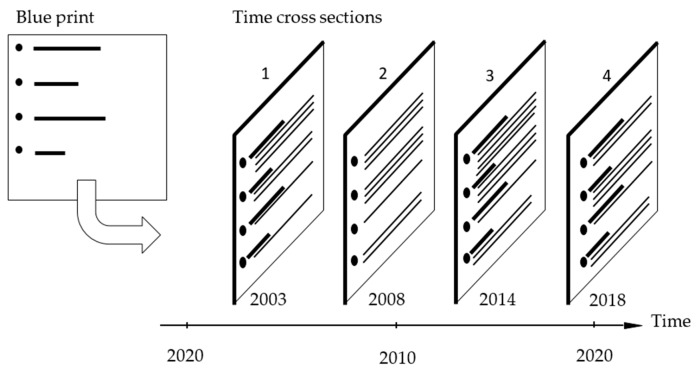
Frame for a longitudinal case study with 4 time cross sections.

The time period is chosen in such a way that a maximum time span in which the researchers can oversee from personal experience is included. In this case study, that leads to 2003–2018 on the basis of the first authors’ involvement with MHC quality and education activities. Practical considerations led to a selection of four time sections. A minor deviation from the theoretically ideal split of the 15 year time period in three equal five-year intervals was introduced, since in 2013 a major ETP change was being prepared, but took until early 2014 before it was completed and introduced. This resulted in time sections in the years 2003, 2008, 2014 and 2018 (See Figure 1).

### 2.4. Quality of Information Sources

Scientific literature was gathered from a variety of peer-reviewed journals, books and congress papers via Scopus, Google Scholar and Research Gate on the internet. This study could not have been done without the use of “grey” literature [22], in this case mostly internal documents and reports made available by ISZW.

### 2.5. Survey

Finally, a survey was held to verify, substantiate and interpret the findings. To this end a series of survey forms was prepared (in Dutch) by the authors with questions for two groups:

(a) senior management at the ISZW-MHC, and
(b) MHC inspectors, all being participant in the ETP in one of the time sections.

The methodical guidelines of Baarda and De Goede [23] were followed. The survey was announced via ISZW management and then distributed via e-mail. In this way the respondents could reply at a time and place they prefer. The response was on a voluntary basis. Prior to the survey an introduction and summary of the findings was made available to each respondent. Respondents were asked to look at these findings and think about any comments or additions they might have. The respondents were asked several “open” questions and were invited to freely express their thoughts on the matter at hand. Also, they were stimulated to talk about experiences they might recall from their own participation in the ETP. Finally, the respondents were asked to mention strengths, weaknesses, opportunities and threats as they felt necessary in relation to further development of the ETP. 

## 3. Theoretical Framework 

The quality of a safety inspection is of critical importance to both the company and the regulator, since the safety performance of a MAHE company is at stake here. Not only criticism by the general public influences the regulators’ choices in the MHC inspectors’ task definition, professionalism, education and training. Many more aspects and stakeholders affect ETP quality. So how do we go about evaluation of the quality of a changing ETP in support of MHC inspector’s performance in such a dynamic, complex, negotiated and politically sensitive field of operation? There are several useful perspectives from which quality appraisal attributes can be derived.

### 3.1. Professional Standards

The regulator inspectors’ task fits within auditing as a professional reference frame. There is an accepted view on how auditing in general should be done and on the professional conduct of an auditor. First of all, the MHC inspector qualifies as a safety professional, facing the challenges of institutional, relational and individual factors in practice [24] (p. 4). Safety professionals such as safety managers, safety consultants and safety inspectors can have different styles of operation, different education and different decision-making power. In practice, they also have different professional identities and express this in different personal styles [25]. Unlike managers and commercial auditors, inspectors must enforce the timely realization of required changes; they enforce compliance [24,26]. 

The inspector role also differs from that of an advisor because an inspector must be independent of any company dealings. There is yet another difference. Unlike specialist advisors, an inspector would normally not be a specialist in the technical field of the companies inspected. Gunningham [27] even calls this asymmetry of information, between regulator and regulate, “*a major issue*”. Nonetheless, relevant experience and knowledge pertaining to the safety of activities in a company, are needed to be able to operate in the void between highly specialized knowledge at the company and the more general knowledge and competencies of a regulator [28]. Where safety management [29,30], safety auditing [31] and risk-based inspection [32] are the subjects of agreed-to industrial standards, the regulator inspector role as such is not [33]. Furthermore, safety management systems can have different form [34] in spite of these standards. Also internal company safety audits can have several forms [35] which can be used at advisors and companies’ own discretion, e.g., to match a chosen safety level. Regulator safety inspection would—ideally—not be confined to any single form since—in principle—all ethical methods, techniques and approaches would be justified to be applied to find hidden unsafety and violations of the law. 

In practice however, MHC inspectors do make use of specific inspection methods and techniques as agreed between the BRZO inspection partners with uniformity and level playing field in mind [13].

### 3.2. Educational Objectives

Krathwohl [36] reviews the taxonomy of educational objectives, originally published by Bloom et al. in 1956 [37], and presents a revised structure in two dimensions: cognitive process and knowledge. The latter dimension is used in this study since it contains a new category: “*metacognitive knowledge*”, which fits rather well with the MHC inspector’s task and position. Inspectors need to assess whether their current skills and knowledge are adequate, given changing strategy, context and conditions. The resulting taxonomy is presented in Table 1.

### 3.3. Quality of Education

According to Van der Hilst [38] (p. 287) there are two general leading principles to be observed in education. Firstly, teachers are professionals, working individually, continuously improving their own performance via researching, training, reflecting and sharing acquired knowledge in support of innovation and development. Secondly, teaching is done best by a collective, several teachers working closely together and jointly guiding students through the complex and diverse curriculum at hand. Swuste and Van Dijk [39] and Van Wassenhove et al. [40] review methods to evaluate the quality of education in occupational health and safety and recommend four quality levels as originally established by Kirkpatrick in 1959 [41,42], see Table 2.

### 3.4. Evaluation Method

Abma & Widdershoven [43] underline the importance of the social relation with stakeholders and a normative framework. They build on the approach of Guba & Lincoln [44], describing fourth generation evaluation as beyond description and judgement oriented, moving towards a *negotiated reality* and truth. Stakeholders, social relations, setting and normative framework are key factors in evaluation.

### 3.5. Education Change Process

**Figure 2:**
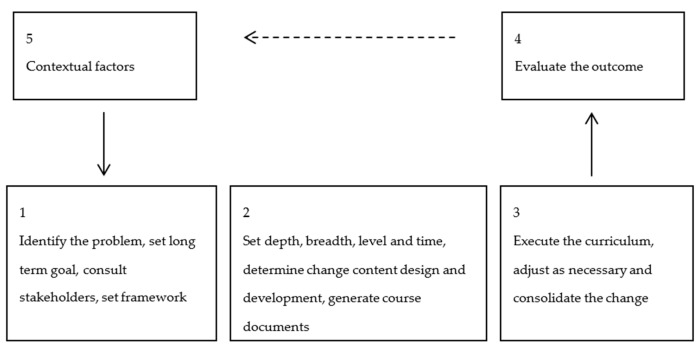
Change process model, simplified from Evans & Henrichsen [45] and Walkington [47].

A *higher education* level is a pre-requisite for MHC inspectors. A *process model* for implementing change in a higher education curriculum was proposed by Evans & Henrichsen [45]. They analyse several ways to go about change, define its four key *dimensions* and propose a strategy for *long term incremental change* after Cuban [46]. A large proportion of the MHC inspectors ETP concerns *engineering knowledge*. Walkington [47] examined curriculum change in higher engineering education and proposed a holistic process model. In Figure 2 the models of Evans & Henrichsen [45] and Walkington [47] are combined into a simplified change process model suitable for evaluation of implemented ETP changes.

### 3.6. Response to the Dynamic Operational Environment

Like in any other regulatory organization the need for change in the MinSZW - MHC approach might originate from a variety of reasons. In this case, triggers for change come from new legislation, ongoing organization, inspection method and cooperation platform development, changing operational context and a new internal vision on education, as well as from external criticism on the ETP and on the activities of regulatory bodies in the BRZO field. Together these situational changes and critical remarks indicate why the ETP should be changed. In this study we consider these triggers as the stimuli for ETP change.

The actual ETP changes, the response to these stimuli, might be reflected in emphasis changes to ETP aspects and their elements, in stopping certain ETP elements or starting new ones, in subtle changes to the contents per ETP element and in the way the course is being conducted. Emphasis, content and response to internal and external triggers are subject of analysis.

## 4. Results

ETP contents information over the time period was retrieved from ISZW internal program descriptions, made available to the authors. This resulted in an ETP contents main aspects structure and allocation of the subject matter to elements within these aspects. The data were organised in four time sections, spread out over the time period. Historical information was gathered from ISZW internal reports on both the ETP execution performance monitoring and on evaluation of ETP contents. Further data about the operational environment was obtained from scientific literature and other public government information. Finally, a survey was held among participants in the ETP over the time period and among ISZW management involved in education and training. The results and analysis sections below refer to detailed data compiled in supplementary files (^1^), (^2^), (^3^) and (^4^).

### 4.1. Program Descriptions

Currently the MinSZW organises the main part of the ETP needed for its own MHC inspectors via the joint learning & development team (LOP team) activity of three Ministries. This team is planning the ETP, contacting the participants about practical matters, coordinating its contents and contracting both internal- and external third party tutors. The program outline and main sections remained intact since 2003, the start of the time period for this case study. This fixed main structure allows, in principle, a series of comparisons over time. In practice however, such a common structure was not intentionally designed and adhered to over the years. A common main structure must therefore be derived from available documentation, allowing allocation of all ETP activities in a logical manner over the entire time period.

A detailed description of the ETP for each of the time cross sections in this study was obtained from internal ISZW information, allowing a detailed compilation of contents and course duration; see the inventory in data file (^1^). The documents as retrieved, listed in data file (^2^), vary in their description of ETP contents in sequence, wording, detail and duration per ETP subject. As expected, they were not directly suitable for a direct comparison over time.

### 4.2. Main ETP Aspects Structure

A common *ETP main program structure* was derived from these documents, valid for all four time cross sections. The program starts from the moment new inspectors are recruited.

This main structure has common *program phases* and deals with common *aspects* (See Table 1 “Aspect” column) in a *common time sequence* in its contents.

Candidates, admitted to MinSZW in order to become MHC inspectors, are selected on basis of a set of *personal qualification requirements*, concerning personality, education level, knowledge and relevant experience. Then they may be allowed, in individual cases, to complete their *safety pre-education* to technical college or master degree level, thus completing their (1) **job requirements**. At the same time their *internal education and training program for newcomers* starts. This deals with general aspects of their work in the MinSZW environment, administrative routines, law enforcement skills, occupational safety, competencies and getting acquainted with the available internal and external support facilities. It also addresses (2) **general legislation**. Then, with the candidates equipped for- and prepared to act as an inspector on behalf of MinSZW, the dedicated *BRZO regulator MHC task related education and training program,* further referred to as ETP, is next.

This program, the focal point of this study, addresses (3) **specific legislation**, prepares the inspector for the (4) **cooperation** setting with inspection partners. It also addresses (5) **how MAHE chemical companies work**. The range of requirements which MAHE companies must meet under BRZO 2015 legislation, the Dutch implementation of the 2012 Seveso III directive [2], fills the (6) **Safety Management System** (SMS) part of the program. A considerable part of the MHC-ETP is spent on (7) **process installation** design and safety issues associated with (8) **dangerous chemicals**. 

The MHC-ETP consists of a larger part being organized within the MinSZW and LOP team environment and two smaller parts which are currently being coordinated from respectively the inspection partners cooperation environment (BRZO+, joint office) and the Ministry of Justice environment. The former is the joint inspection and reporting operation behind each multidisciplinary BRZO inspection team visiting MAHE companies in The Netherlands. The latter allows the MHC inspectors to gather incriminating information and bring suspected criminal act cases to court as necessary. Finally, the MHC inspectors participate in regular meetings, where (9) **learning from accidents** and (10) **learning from practice** during inspection are the key issues. Over the years they also participate in an *ongoing update and repeat program* to maintain their knowledge and skills level and to keep up with new developments, relevant to their work in the BRZO field. In this way they receive (11) **coaching** in support of their work.

These steps (1) to (11) in the main program aspects structure, remained the same over the entire time period. This allows structuring the ETP content for each time section in a similar way, making it suitable for comparisons over time.

### 4.3. Unified Content Elements

Next, a sub-*structure* was created within this main structure to obtain a finer *grid* for allocation of all the ETP fragments found in any ETP history document retrieved. Within each aspect an aggregate was compiled of related content found over all four time sections. This aspect-content was divided into small *elements* on basis of similarity, in such a way that the contents of a single time section could be composed by a combination of several of such small elements.

Since element names, descriptions, terminology and contents per element were all changing over time, resolution of ambiguity was necessary in some cases. This led to *unified ETP element descriptions*, valid for the entire time period of this case study. These were sequenced according to the 2018 ETP version description for convenience.

While all the aspects are present in each time section, these unified elements can be either present or non-present in a specific time cross section. When comparing between time sections, their *presence* indicates e.g., which ETP element stayed over the entire time period, which was left out and which was newly introduced, and which was subject of alteration in content. In other words, this shows how the *ETP subject matter,* evolved over time. If an element is present, its *duration* is expressed in *course-hours* allocated to each unified ETP element in each of the ETP executions per time cross section (^1^). 

### 4.4. Time Cross Sections ‘Blue Print’ Template

With this aspects-, unified ETP elements- and course-hours structure in place it is possible to start building the *blue print template* [15] for this case study. Such a template must consist of three parts, dealing with: *subject matter*, *contextual factors* and *researcher bias* [17,21]. 

The ETP subject matter part of the template consists of the above described aspects 1–11 (see Table 3), each consisting of up to 6 related unified ETP content elements.

The contextual aspects part of the blue print was derived from a previous case study by the current authors [48]. These aspects: **Safety inspection approach, Reporting system, Appraisal and ranking, Public information, Negotiations, Law enforcement, Create safety knowledge** and **Regulator organisation**, were numbered 12–19 (see Table 3). 

Next, the **Researcher bias** aspect [21] was included as number 20 (see Table 3). 

Finally, some five theoretical reference frame related aspects: **Professional standards, Change process, Evaluation practice, Educational objectives** and **Quality of education**, were numbered 21–25 (see Table 3). These are included to represent the appraisal attributes derived from the theoretical framework. The resulting blue print template with all aspects is shown in Table 3. 

The template was then used to create a backbone structure for an *overview* of the ETP subject matter aspects and their contents, expressed as description, presence and duration per element, per time section. Similar overviews were added for the contextual aspects, the researcher bias aspect and the theory based appraisal attributes (^1^).

Rather than crafting four time section descriptions [49] for 25 aspects, a separate longitudinal overview description per aspect was generated from available governmental and scientific sources. These 25 longitudinal descriptions per aspect contain a detailed record of the change history over the four time sections (^2^).

### 4.5. Internal Monitoring and Evaluation of the ETP Execution Performance

The ETP execution performance was monitored and evaluated after program execution, using questionnaires to be completed by the program participants. This information was available for the 2003, 2014 and 2018 executions. Observations on execution performance, summarized from these sources (^2^) are:

2003 ETP execution:

- A general introduction about the course, what contents and why, was missing.
- An overlap with HVK pre-education content was noticed.
- There was a strong accent towards knowledge, participants prefer more practical training.
- Preferably interactive learning style and cases from inspection practice.
- Large differences between teachers.
- Some (theoretical) subjects are not useful in practice.

2014 ETP execution:

- Participants had very different pre-education, background and experience.
- Participants were hired up to some 2 years earlier meaning that the ETP content was for a part overlapping with knowledge and experience already acquired.
- Some of the content was found to be too remote from practice.
- Involvement of company experts had limitations: they were sometimes pursuing their own goals, dealt with a subject in a general or non safety-oriented way and could be the opponents of inspectors at some point in time.
- Some presentations were very mathematical and theoretical and far from practice.
- The course had too many short presentations on some days. This leaves no time for interactive learning. A full day concentrated and passive listening was exhausting.
- The course should ideally be held within 6 months after inspectors are hired.

2018 ETP execution:

- Several participants did not take part in 2 or 3 course days due to other obligations, making their individual run through all seven SMS elements incomplete.
- An examination at the end of the SMS oriented section of the course was considered to be a good addition to the ETP.
- The BRZO related legislation and the inspector toolbox took more time allocated in the ETP.

### 4.6. Internal Evaluation of the ETP Contents and Teaching Style

The executions of the ETP were internally evaluated several times, in 2013 and in 2017, both in support of the preparation of the next ETP execution a year later, in 2014 and 2018 respectively (^2^). 

Observations on contents and teaching style, derived from these sources are:

2013 ETP contents evaluation:

- The general introduction about BRZO can be shortened,
- learning from what can go wrong needs to be an explicit part of the ETP,
- time spent on reliability evaluation of installations can be significantly reduced,
- process safety needs to be extended with kpi, ageing, plant layout and software architecture,
- a new section on culture, procedures and language issues needs to be added.

2017 ETP contents and teaching style evaluation:

- A new active teaching style is needed: less presentation, more interaction, active learning, challenge, feedback, informal setting, participants know what is expected of them, participants lead and present,
- regroup and extend the ETP contents to ensure that each of the SMS elements are dealt with, both separately and jointly,
- increase the focus on risk management, both on risk inventory and risk analysis methods,
- explicitly deal with legal requirements and how these can be met by companies via compatibility and measures taken,
- use information and examples from inspection practice, and
- use homework, practice training and e-learning as necessary.

### 4.7. Scientific Literature and other External Government Information

Several external scientific studies and Government studies on regulator performance were found within the time period. The following observations on regulator performance were derived from these sources. 

Between 2003 and 2012 the Labour inspectorate MHC department published a series of status reports [50,51,52,53] describing the incidents investigated and their causality. Poor risk assessment, non-standard operating conditions such as start, stop or maintenance of installations, design flaws and poor supervision during safety critical work are persistent issues.

In an evaluation study consultants of KPMG [54] advise more uniform conduct by inspectors and a more auditor style approach. Also the development of guidance for appraisal of a company SMS is recommended.

The joint cooperation platform, BRZO+ (Previously called LAT-bureau) investigated the opinion of MAHE chemical companies in the BRZO field about regulator inspection performance. Several improvement possibilities were identified both in general and for each of the partners, i.e., also for MHC inspectors. Uniformity of the MHC inspectors’ style across the country is insufficient, in spite of ‘account management’. The readability and delivery time of inspection reports are flagged up as insufficient [55].

In 2008 Hulst-Ouwerkerk [56] investigated the results of MHC criminal law enforcement activities. The impact of such enforcement appeared to be rather small.

In 2012 Ale & Mertens [57] distinguish two main regulatory directions within an SMS: A) rules oriented inspection and associated law enforcement, and B) system oriented governance leading to risk management with help of an SMS and company made rules. The latter is more difficult to appraise by an inspector since expert knowledge is required but also because company’s financial interests may differ from investing in safety. The expert knowledge of regulator inspectors is critical here. 

In 2013 Lieshout [58] evaluates BRZO 1999 activities from inspection practice point of view and concludes that companies should—given their dynamic situation — continuously and critically evaluate their risk inventory. The regulator should at the same time focus on risk based inspection. The fragmented regulatory organisation should be condensed in fewer institutions and cooperation among them should be further improved. 

In 2015 In ‘t Veld [59] looks back over the preceding 15 years and finds that regulatory activity apparently has not reduced the major accident rate in the Netherlands. In ‘t Veld argues that fragmented government regulatory institutions and ineffective law enforcement have contributed to persistence of major accidents, as well as weak company self-regulation. Based on this failure of regulatory policy of trust and relying on company self regulation, a stronger independent national institution for law enforcement is proposed [59]. In 2017 Besserman and Mentzer [60] investigate major hazard chemical safety regulations in several countries and present a steady decrease of fatal accident rates per 100,000 workers in industry in the EU, which may serve as a background here. 

The need for the regulator to keep inspecting was confirmed in 2017 by Ale et al. [61]. 

In the same year Swuste & Reniers [62] evaluate the effectiveness of the Seveso directive inspections in Belgium and The Netherlands. They analyse nine reports issued in the period 2004 – 2014 about effectiveness issues and improvement possibilities in the Seveso II and III directive regulatory domain. The importance of a company SMS and the need for a better structured governmental approach of process safety as a common area of interest were underlined. Several cooperation- and organization issues between the three main inspection partners were mentioned. Partners’ inspectors use different approaches, depending on the inspection partner they belong to. Jointly developed training courses and a common inspection method were introduced. Although the needs for expert knowledge among inspectors, their higher education level and the existence of education and training programs, were mentioned, no further specific recommendation was made on their education and training program. 

On the efficacy of the Seveso III directive [2] implementation in the Netherlands, “*no clear and convincing findings*” could be reported by Swuste & Reniers in 2017 [62].

In 2018 Swuste & Sillem [63] examine the quality of the MoSHE (Management of Safety Health and Environment) academic course, one of the two preferred types of external safety pre-education for MHC inspectors. They conclude that ”*greater input of process safety and safety in high-tech -high-hazard sectors*” could improve the course. Several Moshe student-inspectors have contributed by means of their thesis to the development of MHC practice. And so did the students of the other pre-education type, Hogere Veiligheids Kunde (HVK), which has not been evaluated in the time period between 2003 and 2018.

In 2018 Pronk et al. [64] present an analysis of BRZO-policy in the Netherlands with the development of a future vision up to 2030 in mind. One of their observations is that environmental safety is governed via an acceptable risk level whereas worker safety is governed by a minimum risk level, achievable via continuous improvement, by the *state of the art* and by the pragmatic As Low As Reasonably Achievable (ALARA) principle. 

Also that year, Wingerde et al. [65] performed a case study on risk based administrative law enforcement activities of ISZW inspectors on worker safety. Their main observations are that:

- the role of inspectors became more confined to enforcement,
- spotting risks is done centrally at MinSZW rather than by the inspectors in the field,
- the inspectors’ work is standardized and prepared for optimum juridical uniformity, and
- the introduction of much higher fines has resulted in more juridical activities for inspectors.

## 5. Analysis 

### 5.1. Trainee and Management Appreciation Survey

A summary of the above findings was presented per e-mail to MHC management/staff and to MHC inspectors. Both groups (trainees and management) were asked to express themselves about their appreciation of their experiences with the ETP. 

All together some 63 questionnaires were sent to these groups, covering 100% of the people involved with the ETP development, management or participation in the SZW environment. The response consisted of 31 completed questionnaires, hence a 49% response. The survey data were then collected, analysed and interpreted (^3^), leading to the results below. The questionnaire contained open questions and closed questions.

*Closed questions*

Closed questions were used to allocate respondents to the 4 time sections and gather facts about their function, their involvement with the ETP, when they followed the ETP, whether their acquiring of knowledge was subject of testing, whether they used it in their work, and whether it improved their work. Main observations were:

- The response of ETP participants from time periods 1 and 2 was significantly less than from time periods 3 and 4.
- About 70% were inspectors, the remainder was management or staff.
- Respondents indicated that the ETP mainly aims at HBO education level (67%) and Academic level (25%).
- Out of the 4 types of knowledge to acquire (facts, concepts, procedures, meta-cognitive) the respondents rated facts the highest (30%) and meta-cognitive the lowest (19%).
- Some 50% stated they were not tested on how well they acquired knowledge or skills from the ETP.
- The vast majority (80%) confirmed they used these in practice and felt their work was improved (68%) and provided examples.

*Open questions*

Open questions were used to assess opinions about participants’ own experiences, ETP changes over time, quality, strength, weakness, opportunity and threats in relation to the ETP and about the way the ETP could be improved. 

The resulting survey response on open questions revealed no comments as to the correctness of the 25 aspect descriptions (^2^) presented in summary form in the explanatory introduction, sent together with the questionnaire to all respondents. Consequently, no need for change in the blue print template was identified from the survey response.

Answers to open questions were summarized in reactions per question. Then the reactions were split-up into text parts addressing separate subjects. These text parts were clustered on basis of similarity and reformulated into a range of 11 *issues* (see Table 4). 

Main observations are:

- The issues were then ranked on basis of the frequency count of respondents addressing them. This results in the list of 10 main issues as presented in Table 4.
- A comparison between issues mentioned by participants in time sections 1 and 2 only, and issues mentioned by all participants in all sections, shows that “keeping up with recent BRZO field developments” is perceived as more important by more experienced inspectors (Table 4, row J).
- Comparison between the issues raised by inspectors and those raised by management/staff, shows that the latter group more strongly feels the need to test the knowledge acquired through the ETP (Table 4, row F).
- Management/staff are less concerned about avoiding knowledge loss by faster application of the teachings in inspection practice (Table 4, row B).
- Inspectors which most recently went through the ETP underline the high workload and the limited exposure to learning from practice during field work (Table 4, row B).
- Inspectors generally note that abstract, theoretical or detailed knowledge is not applicable in their work (Table 4, row E).

Several specific subjects are frequently mentioned by participants, underlining their relevance for inspection practice. These are: Explosive atmospheres [66,67], Pressure equipment [68], Dangerous substances [69,70,71], Occupational safety [5], law enforcement [5,6,7], PGS standards [72], Audit method [31], Implementation guidance [73], and various safety management and analysis methods and techniques, mostly originated from practice (See Table 4 rows D and G).

### 5.2. ETP Emphasis and Content Changes in Response to Internal and External Triggers

A first analysis is conducted to determine whether more or less course-hours were allocated to content elements over time. The course-hours spent on any particular subject are used as a proxy for emphasis.

A second analysis is used to search the 25 aspect descriptions and establish *what actually changed* in the ETP contents, the context and the way the MHC education and training program is being conducted. 

The information gathered from the contextual aspects, the execution performance monitoring, the internal contents evaluation, scientific literature and governmental reports on BRZO developments and on regulator performance, are used for a third analysis. This identifies triggers for change of the ETP and allocates them to the aspects in the blue print template. The result indicates that there was a response to all of the triggers identified in this study (^4^).

The first and second analyses together show both the nature and the emphasis of the ETP changing in response to external triggers (^1^). This leads to five groups:

1 Some aspects and elements in the ETP were continued over the entire time period. These often showed only minor changes not affecting their demeanor, intent or purpose: In the ETP contents subtle changes were found both in duration and descriptive text. This might for example concern a change in legislation or the choice of a major accident case being presented and discussed. The number of accident cases varies and older cases are being gradually replaced by more recent cases as time goes on. Also many technical elements were kept up to date, not significantly affecting hours spent during the course.
2 Several aspects and elements were stopped: both general and special legislation changes resulted in ETP elements being abandoned during the time period: Law enforcement on occupational safety, details on environmental legislation, safety report contents, partly abandoned legislation, obsolete standards, BRZO law enforcement policy, company view on BRZO, site layout, Dangerous substances Advisory Board, obsolete inspection methods, theory on mass- and flow process balances. This is indicated by decreasing course-hours allocated to aspect 3. Aspect 8 even disappeared for a while before new dangerous substances- and labelling legislation was issued in Europe.
3 A number of aspects and elements did not exist before and were newly started: carcinogenic, mutative and reprotoxic substances, exposure health and safety, environmental law enforcement, a new standard for dangerous substances storage facilities, the joint inspection partners cooperation, the joint inspection partners digital platform, company practices in maintenance of process plants, ageing in process installations, safety management system elements, language related safety risks, key performance indicators on BRZO plant safety, improving work instructions, explosive atmospheres directives and standards, pressure vessels regulation, new regulation on dangerous substances- and labelling, law enforcement toolbox, fine system, inspection of safety measures, test site training, new inspection method, the role of an inspection leader, training inspection skills, regular coaching sessions. These new ETP elements appear mainly in aspects 2, 4, 6 and 10 as reflected by their hours increasing over time.
4 Some elements appeared as a “one-off”, they were part of the ETP only once. Certification of safety management systems, BRZO evaluation, Management standards on quality [74], environment [75] and occupational safety [76,77], law enforcement powers, document control, MHC course evaluation by participants, occupational accident investigation, when and how to stop process plant activities, interaction between plant design and safety measures.
5 Few elements were reduced in allocated course hours, thus receiving less emphasis. This concerns various theoretical subjects about process safety analysis, about probability and on failure data. This shows from aspect 7 course-hours which are steadily dropping from 2003 up to 2014. Exception here is a substantial increase in education effort on technical information about process installations. These course-hours rise to 68 in 2018, significantly above the 2003 level of 40 h.

### 5.3. Observations on Quality over Time

Regarding quality of the Dutch MHC inspector’s ETP over the 2003–2018 period, several observations can be derived from the results and analyses described in the above. 

2003

During the first time period, ending in the year 2003, the MHC inspection of major accident hazard establishments was subject of organizational improvements. Joint MHC meetings to keep inspectors informed were held. In time cross section 2003 a dedicated MHC directorate, the cooperation between inspection partners, uniformity, reporting methods, auditor style of operation and execution performance, were key issues requiring improvements. The ETP was theoretical, technical and static and had technology oriented content. Participants were passive listeners.

2008

During the second time period, starting from 2004 and ending in 2008, inspectors, management and third parties involved, were mainly focusing on inspection method development. A joint cooperation platform between inspection partners was established. Rules for joint inspection partners reporting were agreed. New European directives on pressure equipment and explosive atmospheres were introduced, thus placing new requirements on the ETP content. New standards for safety management appeared. Joint inspectors’ meetings were organized with inspection partners to keep up with developments. Education became a joint team activity between the 3 ministries. The contents of the ETP were made more practice oriented, were updated with technological developments and with recent accident history data. A joint inspection partners’ digital reporting system and appraisal platform was built. The ETP was kept largely the same, hence theoretical, technical and static and still had little practice oriented content. Minor additions and changes in content avoided that it was lagging behind with respect to technical developments. Safety culture, risk assessment, non standard conditions of operations and installation design- and ergonomical aspects became main concerns which were missing out in the ETP. Participants were passive listeners.

2014

A changeover from criminal law enforcement to administrative law enforcement increased the impact on MAHE companies by use of the inspectors’ law enforcement toolbox. A new version of the Seveso directive introduced several safety management requirements to be imposed on MAHE companies. The aging of installations became a main concern. Informing the general public about inspection results was initiated. The attention for human factors and safety culture was increased. The education program received a major overhaul from a contents point of view. Theoretical content was reduced and yet more practice oriented content was introduced. Human factors, a.o. safety culture and language issues, were introduced in the ETP. The contribution by technical company expert tutors was reduced and the involvement of internal and external safety professionals was increased. The skills of tutors/trainers and their teaching style became an issue of concern.

2018

In response to internal discussions, a.o. in the LOP team, about the effectiveness of the ETP, a new set of education originated requirements was established: (inter)active teaching style, content structured along the SMS elements in the Seveso III directive, increased attention for risk assessment, emerging technology and process safety aspects. New ways of learning were introduced such as e-learning and practical training sessions in a test factory. The law enforcement toolbox was included in the ETP. The education program became—for the main part—outsourced to a limited number of external contractors, specialized in education and in safety. A small part was kept in-house to be handled by internal ISZW knowledge centre experts and by individual senior inspectors. Ongoing developments in the inspection methodology could not be implemented in the ETP by 2018, since their finalization is expected after the 2019–2020 outsourcing contracts were settled. The survey shows that, for participants, the ETP content itself, i.e., the subject matter currently dealt with, is no longer a main issue of concern. Safeguarding the existing quality of the ETP and the tutor/trainers‘ skills is their first main issue. Their second main issue is loss of acquired but then unused knowledge. When knowledge is offered by a tutor/trainer during the ETP execution but it is not soon after, efficiently or effectively coupled to an experience in practice, respondents argue this is leading to loss of knowledge. Although an examination was introduced at the end of the safety management part of the ETP, participants indicate this should be further developed and introduced also elsewhere in the program (see Table 4).

### 5.4. Change Process

Change in the ETP over the entire 15 years time period was primarily driven by MinSZW management initiatives, responding to external triggers, internal monitoring- and performance evaluation results. Although the change process itself, most clearly in the first 10 years, did not concur with the change process model as derived from Evans & Henrichsen [45] and Walkington [47], the process model was rather closely followed during the last 5 years.

## 6. Discussion

### 6.1. Researcher Bias

The researcher bias, aspect 20 in the blueprint template, primarily concerns the researcher perspective changing over the time period. Although the authors did have changes in their perspectives, we contend that the effect this bias might have is compensated by involving MHC inspectors and ISZW management for a verification of the results.

This study describes the 15 years evolution process of the ETP from an auto-ethnographical insiders’ perspective [78]. The authors discuss the changes and their merits as seen from the limited perspectives of the inspectors and the regulator institution. Other stakeholders within the operational environment such as the HRO chemical companies, other regulator organizations within the group of joint inspection partners and the population living in the direct vicinity of MAHE company sites were not involved in this study.

### 6.2. Validity and Reliability

This descriptive case study is also sensitive to the method bias which is caused by too little separation between predictor- and criterion variables [79]. To ensure validity, the ETP description information and performance monitoring information were taken from internal MinSZW documents and evaluation data were for the most part obtained from several other, separate and independent, sources outside MinSZW.

Also the case study descriptions may be sensitive to ambiguity in the terms used to describe the unified ETP elements [79]. Vague and indeterminate words such as “many” or “sometimes” were avoided in the descriptions for this reason.

The currently available information, placed in an evaluation frame derived from Krathwohl [36] and Swuste & van Dijk [39], would result in an incomplete evaluation.

### 6.3. Thinking about the Paradigm

The focus in a company needs to be on *safe production*. This requires actively reducing known hazards which—if left unattended—may cause accidents, economical damage and poor safety performance among personnel. Working safe is good for the company *bottom line* [80]. 

A health & safety manager can best adopt a *supportive, inspirational leadership style* [81]. 

The *authoritarian leadership style* for improvement of behaviour, based on the power of position and use of confrontation, has been tried over and over again. The results are certainly not as strong and positive as people like to believe. As a concept it has been proven ineffective in addiction treatment [1], was found to be flawed in parenting [82] and turns out to be ineffective in rectifying unsafe behaviour among company personnel [81,83]. So, why would an authoritarian style be considered sensible, logical or even feasible as a tool to adjust poor safety performance at any MAHE chemical company? 

MHC inspectors, with their Government regulator position, equipped with a powerful administrative- and criminal law enforcement toolbox, having *considerable discretionary authority* and using *framing behaviour* attributing *moral qualities to businesses* [33] would fit the authoritarian role, at least at first glance. 

The authors contend nonetheless that the MHC inspectors’ professional expertise, supportive approach, reluctant attitude towards the use of discretionary competency and using the power invested in the law enforcement toolbox, *all* appear to be necessary. The way of weak regulatory action and self-regulation by companies is not effective [59]. The often observed *risk regulation reflex* with instructions about *zero tolerance* and being *tougher in enforcement and controls*, achieves little [84]. In terms of objectives and outcome, in the time period, the impression among stakeholders involved was that MAHE safety is not improving over time, in spite of the regulatory BRZO effort. The Dutch government policy was changed accordingly and the basis for the inspector’s law enforcement task changed from criminal law to administrative law. It is at this time too early to assess the effect this changeover might have. So, would teaching the already knowledgeable and skilled inspectors to exert *tough love* [1,85]—staying between *too soft* [59] and *too hard* [84]—actually be the right direction to proceed towards more professionalism when it comes to further development of the MHC inspector ETP?

## 7. Conclusions 

In hindsight, the progressive changes in the Dutch MHC inspector Education and Training

Program during the time period 2003–2018 closely followed the development of the European Seveso directive with its subsequent implementation in Dutch legislation. 

Professional standards could be used more to elicit the MHC inspectors’ role and support the appraisal of the inspectors’ performance in practice. The metacognitive objective level in the MHC inspectors’ knowledge receives little attention in the ETP.

Looking at the quality of education it appears that, both the process of changing the ETP with its educational objectives in response to the dynamics of the MHC field, and the evaluation of the ETP content are not structured in a systematic way according to current theoretical insights. 

The current ETP execution performance monitoring records are limited and do not facilitate internal ISZW evaluation of the ETP content and the teaching style on a regular basis.

External studies on regulator performance in the BRZO field do touch upon the content of the MHC inspectors’ ETP. However, even when taken together, the coverage by these studies is incomplete at best, and they cannot replace a systematic evaluation. The “blue print” as developed in this study may serve as a starting point here.

Nonetheless, the authors conclude that the ETP development adequately responded to the dynamic operational environment and the safety related problems in industry, since no triggers were found to remain unanswered. Both the effects of the recent changeover to administrative law enforcement strategy and the ongoing search for improvements of the inspection style, pose a challenge for future research. The authors recommend investigation of the possibilities for setting up an evaluation reference frame for Seveso inspector’s education in the European Community and for the introduction of some form of methodical preparation for Seveso III related ETP evaluation. 

## Figures and Tables

**Table 1:** Knowledge dimension taxonomy for evaluation of education objectives, simplified from Krathwohl [36].

Objective Level	Knowledge	Attributes
A	Factual	Terminology, specific details
B	Conceptual	Cooperation, setting, safety systems
C	Procedural	Methods, skills, criteria, tools
D	Metacognitive	Strategy, context, conditions, self-knowledge

**Table 2:** Quality levels in Health and Safety education, derived from Swuste & Van Dijk [39].

Quality Level	Primary concerns	Attributes
1 Reaction	Trainees’ appreciation	Course organization, teacher style
2 Learning	The participants are learning	Variety of in-course test techniques
3 Behaviour	Participants are using and applying it	Post-course poll among participants
4 Impact	There is a beneficial impact on their work	Stakeholder survey

**Table 3:** Blue-Print template for time cross-sections for the Education and Training Program.

Nr	Aspect	Content Elements
*ETP Subject matter*		
1	Job Requirements	Assessment, Pre-Education, Experience, Personality, Behavior, Safety pre-education
2	General legislation	Occupational Safety, Environment, Legal Action
3	Special Legislation	Seveso Directive, Dutch legislation, Competencies
4	Cooperation	Inspection partners introduction, Joint GIR-platform
5	How companies work	Presentations by MAHE chemical companies, Company practices.
6	Safety Management System	Safety Policy, SMS-Elements, Appraisal, Risk management
7	Process installations	Design, Technical safety, Explosion, Pressure, Life cycle phase related activities
8	Dangerous chemicals	Hazards, Labels, Legislation
9	Learning from accidents	Accident History, Stakeholders, Investigation Methods, Records &Systems
10	Learning from practice	Test-site, Company visits, Joint inspections, Inspection skills
11	Coaching	Team-meeting, 3rd Party Training Courses, Specialist knowledge, Support facilities
*Context*		
12	Safety Inspection Approach	Scope, Attitude, Focus, Inspection Method, Frequency, Access to information
13	Reporting System	Writers, Content, Uniformity, Public/Proprietary
14	Appraisal/Ranking	Parameters, Appraisal criteria, Ranking method
15	Public Information	Inspection results, Ranking results, Law enforcement results, Transparency
16	Negotiations	Standards, Public-Private network
17	Law Enforcement	Tools, Activity Stops, License to Operate, Legislation
18	Create Safety Knowledge	Evaluation Studies, Safety Information, Safety Research, Regulator Experience
19	Regulator organization	Paradigm, Economy, Public debate, Political situation, Manpower, Edu & Training
*Researcher*		
20	Researcher bias	Authors involvement, change of perspective, limited view, method bias
*Theoretical framework*		
21	Professional standards	Auditing, Professional style, inspection methods & techniques
22	ETP Change process	Set goal, determine content, execute, evaluate the outcome, implement change
23	Evaluation practice	Stakeholders, Social relations, Setting, Normative framework
24	Educational objectives	Factual, Conceptual, Procedural, Metacognitive
25	Quality of education	Trainee appreciation, Learning, Usage in practice, Societal impact

**Table 4:** Ranking of issues raised by the ETP participants and management/staff; Comparisons between earlier sections only and all sections and between management/staff only issues and all issues for all sections; in percent.

Row	Issue Description	% All Issues 2003 2008 2014 2018	% All Issues 2003 2008	% Mgmt./Staff Issues 2003 2008 2014 2018
A	Safeguard the ETP quality with its didactically skilled and experienced tutors, and its form with discussion-, (social) interaction-, group activities- and active knowledge sharing techniques.	13	12	11
B	Busy education program while attempting to gather experience from practice to avoid loss of new knowledge due to not using it.	13	6	8
C	Achieve knowledgeable, skilled and self-confident inspectors, by uniformity in their training and inspection approach and mutual interchangeability.	11	12	13
D	Sufficient attention for mandatory specific subjects: Legislation, BRZO, PED, ATEX, Aging, Safety Report appraisal, law enforcement, occupational safety, scenario’s, emerging technology, human factors, uniformity of regulation. (^1^)	10	12	9
E	Better balance between theoretical, detailed and abstract education content and usability in inspection practice.	9	6	4
F	Increase testing in several ways whether the content is well understood and the knowledge as presented in the ETP is acquired.	9	15	13
G	Implement more connection between the moment theoretical knowledge is offered in the ETP and the first moment of using the acquired knowledge in practice to improve learning. Opportunities are: ATEX, PED, Occupational safety, Law enforcement, Work Permit, LMRA, TRA, SIL/Lopa, Audit trail, HAZOP, PGS-6, Risk evaluation, process safety, KPI’s, Language. (^1^)	5	6	8
H	Increase activity on regular evaluation of the ETP on: form, content, quality, actuality, efficiency, efficacy, innovativeness and on the improvement process.	5	6	8
I	Organize more repeat- and updates training for inspectors to safeguard expertise and skills and avoid differences in inspector’s knowledge and skills.	3	0	4
J	Continuously develop the ETP to keep up with changes in legislation, law enforcement, the BRZO field, inspection procedures, the process industry, society, didactic view, emerging technology, human factors. Make more use of senior inspectors and ISZW knowledge centre and implement more e-learning.	3	9	8
K	Other issues	18	15	15
	Total	100	100	100

(^1^) Abbreviations: PED: Pressure Equipment Directive; ATEX: ATmospheres Explosives; LMRA: Last Minute Risk Analysis; TRA: Task Risk Analysis; SIL: Safety Integrity Level; Lopa: Layers Of Protection Analysis; HAZOP: Hazards And Operability Study; PGS-6: Standard nr 6 in PGS series; KPI: Key Performance Indicator.

## Supplementary Materials

The following are available online at https://www.mdpi.com/1660-4601/17/6/1959/s1: (^1^) Data file 1—ETP contents, context and duration per time cross section and Blue print aspects, including a compilation of ETP response to external triggers for change. (^2^) Data file 2—Blue print 25 Aspect history case descriptions for all sections with references. (^3^) Data file 3—Survey data on participants and management appreciation. (^4^) Data file 4—External and internal triggers for change.
