# Supplementary material for: Improving Education and Training of Dutch Major Hazard Control Inspectors: A 15 Years Longitudinal Case Study
Source: Int J Environ Res Public Health. 2020 Mar 17;17(6):1959. doi: 10.3390/ijerph17061959 (PMC7143160; doi:10.3390/ijerph17061959)

Time section specific

LINE NR	BLUE PRINT NR	BLUE PRINT ASPECT<br>ETP SUBJECT MATTER<br>ASPECT GROUP	BLUE PRINT CONTENT ELEMENT<br>ETP ELEMENT	ETP Main programme structure Nr	Sub nr	ETP Sub-sections structure	ETP 2018 Element Code	ETP Unified Element description	2003 Presence & Duration	2003 Duration hours	2008 Presence & Duration	2008 Duration hours	2013 DIWI opl codes MHC . . . En tijdsduur	2013 Duration hours	2014 Presence & Duration	2014 Duration hours	2018 BOP Presence & Duration	2018 Duration hours	2019 Tender OMI Presence & Duration	2019 Duration hours	A Continued, minor changes<br>B Stopped<br>C Newly started<br>D One off<br>E Reduced	A	B	C	D	E
				personal qualification requirements																						
1	1	Job Requirement	pre-education, experience	1	1	Recruitment	Qualifications	pre-education, education level, knowledge, relevant experience	yes		yes		yes		yes		yes				A	1	0	0	0	0
2	1	Job Requirement	personality	1	2	Recruitment	Job interview	personality	yes		yes		yes		yes		yes				A	1	0	0	0	0
3	1	Job Requirement	Assessment, behaviour	1	3	Recruitment	Assessment	background, drives, behaviour, skills	yes		yes		yes		yes		yes				A	1	0	0	0	0
4	1	Job Requirement	Safety education	2	1	Safety education	HVK	technical college level (HVK)	yes		yes		yes		yes		yes				A	1	0	0	0	0
5	1	Job Requirement	Safety education	2	2	Safety education	MoSHE	master degree level (MoSHE)	yes		yes		yes		yes		yes				A	1	0	0	0	0
										0		0		0		0		0		0		5	0	0	0	0
6	2	General legislation	Legal action	3	3	legislation	SZW WA	Wetgeving alg. AWB, WED, Bevoegdheden, Rechtspersonen, Boete	WA	18	WA	18	B01, 3 dagen	18	yes	18	yes	18			A	1	0	0	0	0
7	2	General legislation	Legal action	3	3	legislation	ARBO WESP	Arbowet, handhaving Strafr / Bestuursr.	WESP	24	WESP	24	B02, 4 dagen	24	yes	24	yes	24			A	1	0	0	0	0
8	2	General legislation	Legal action	3	3	legislation	ARBO HH-ARBO	Handhaving ARBO wet, warenwet	ARBO	36	ARBO	36					yes	3			B	0	1	0	0	0
9	2	General legislation	Dag1.Introductie BRZO	3	6	occupational safety	ARBO CMR	Kankerverwekkende stoffen					AOG04, 0.5 dag	3	yes	3					C	0	0	1	0	0
10	2	General legislation	Dag1.Introductie BRZO	3	6	occupational safety	ARBO BLST	Blootstelling					AOG02, 1.5 dag	9	yes	9	yes	9			C	0	0	1	0	0
11	2	General legislation	Environment	4	5	Dag1.Introductie BRZO	Wabo, VR	Milieu regelgeving en beoordeling QRA in VR	blok6, 3 uur	3	blok6, 3 uur	3									B	0	1	0	0	0
12	2	General legislation	Legal action	6	1	Inspecteursopleiding BOA	BOA basis	boa basis	10 dagen	60	10 dagen	60	10 dagen	60	10 dagen	60	10 dagen	60			A	1	0	0	0	0
13	2	General legislation	Legal action	6	2	Inspecteursopleiding BOA	MILBOA	milieu boa	-		-		12 dagen	72	12 dagen	72	12 dagen	72			C	0	0	1	0	0
14	2	General legislation	Legal action	6	3	Inspecteursopleiding BOA	BOA PHB	permanente her- en bijscholing	-		-		4 dagen / jaar	24	4 dagen / jaar	24	4 dagen / jaar	24			C	0	0	1	0	0
										141		141		210		210		210		0		3	2	4	0	0
15	3	Special legislation	Competencies	3	3	legislation	MHC WA BEV	Bevoegdheden MHC					B05, 1 dag	6							D	0	0	0	1	0
16	3	Special legislation	Dutch legislation	3	3	legislation	ARBO ARIE	ARIE (MHC ARIE taak is nog steeds onduidelijk)	blok2, 6 uur	6	blok3,5 uur	5									B	0	1	0	0	0
17	3	Special legislation	Seveso Directive	4	17	Dag1.Introductie BRZO	VR	VR Globaal overzicht vd inhoud	blok1, 1uur	1	blok3, 1uur	1	A06, 1/3 dag	2							B	0	1	0	0	0
18	3	Special legislation	Dutch legislation	4	12	Dag1.Introductie BRZO	Normen BRZO	BRZO Normen (RIB, CPR20, HUBO, NTA8620)	blok1, 1uur	1											B	0	1	0	0	0
19	3	Special legislation	Dutch legislation	4	13	Dag1.Introductie BRZO	Normen 9-14-18	Management normen (9000, 14000, 18000)							C06B, 1uur	1					D	0	0	0	1	0
20	3	Special legislation	Seveso Directive	4	2	Dag1.Introductie BRZO	LAT BRZO+	met wie wordt samengewerkt bij BRZO inspecties									Dag1,1uur	1	Mod1,Dag1	1	C	0	0	1	0	0
21	3	Special legislation	Dutch legislation	4	11	Dag1.Introductie BRZO	HH BRZO	Handhavingsbeleid BRZO	blok1, 2uur	2	blok1,3 uur	3									B	0	1	0	0	0
22	3	Special legislation	Seveso Directive	4	15	Dag1.Introductie BRZO	Evaluatie BRZO	Evaluatie BRZO					A05, 1/3 dag	2							D	0	0	0	1	0
23	3	Special legislation	Dutch legislation	4	16	Dag1.Introductie BRZO	Certificatie VBS	Certificatie VBS			blok3, 1uur	1	A06, 1/3 dag	2							D	0	0	0	1	0
24	3	Special legislation	Dutch legislation	4	6	Dag1.Introductie BRZO	BRZO	Regelgeving in het MHC werkveld							B06, 2 uur	2					A	1	0	0	0	0
25	3	Special legislation	Dutch legislation	4	61	Dag8.2B Voldoen aan verplichtingen	BRZO verplichtingen	Wat een BRZO bedrijf concreet moet doen									Dag8,1/2uur	0,5	Mod5,Dag8	0,5	A	1	0	0	0	0
26	3	Special legislation	Dutch legislation	4	118	Ontwerp filosofie en procesinstallaties	PGS15	PGS 15 Opslagbedrijven									Dag17, 2 uur	2			C	0	0	1	0	0
										10		10		12		3		3,5		1,5		2	4	2	4	0
27	4	Cooperation	Inspection partners intro	4	9	Dag1.Introductie BRZO	OVV, enz	rol van andere externe organisaties zoals OVV			blok2,1uur	1	A05, 1/3 dag	2	C06A, 1 uur	1	Dag1,1uur	1	Mod1,Dag1	1	A	1	0	0	0	0
28	4	Cooperation	Inspection partners intro	4	3	Dag1.Introductie BRZO	Milieu, BRW	rol van inspectiepartners BRW, Milieu					A04, 1 dag	6			Dag1,2uur	2	Mod1,Dag1	2	A	1	0	0	0	0
29	4	Cooperation	Inspection partners intro	4	4	Dag1.Introductie BRZO	Milieu	Externe veiligheid VROM-Wm, I&M-Wabo	blok2, 3uur	3	blok2,3uur	3			B06, 1 uur	1					A	1	0	0	0	0
30	4	Cooperation	Inspection partners intro	4	1	Dag1.Introductie BRZO	MHC taak	hoe is BRZO georganiseerd binnen SZW			blok1, 6 uur	6	A03, 1 dag	6			Dag1,2uur	2	Mod1,Dag1	2	A	1	0	0	0	0
31	4	Cooperation	Inspection partner intro	4	7	Dag1.Introductie BRZO	BRW	Brandweer (BRW-regio, Veiligheidsregio)	blok2, 3uur	3	blok2,3uur	3									A	1	0	0	0	0
32	4	Cooperation	Inspection partner intro	4	8	Dag1.Introductie BRZO	BRW	Veiligheidsregios							B06, 1 uur	1					A	1	0	0	0	0
33	4	Cooperation	Inspection partner intro	4	18	Dag2.Wetgeving BRZO	BRZO	Seveso directive, BRZO, RRZO	blok1, 1uur	1			B03, 2 dagen	12			Dag2, 6 uur	6	Mod2, Dag2	6	A	1	0	0	0	0
34	4	Cooperation	Joint GIR platform	5	5	Inspecteursopleiding LAT/BRZO+	GIR	Gezamenlijk ICT platform gebruik					A07, 2 dagen	12	yes	12	yes	12			C	0	0	1	0	0
										7		13		38		15		23		11		7	0	1	0	0
35	5	How companies work	Presentations by HRO companies	4	14	Dag1.Introductie BRZO	Company view	Visie bedrijfsleven ivm BRZO			blok2,3uur	3	A05, 1/3 dag	2	B06, 2 uur	2					B	0	1	0	0	0
36	5	How companies work	Company practices	4	19	Dag3.Onderhoud BRZO bedrijven	OH praktijk	Onderhoud bij BRZO bedrijven in praktijk					C04 (in ontw)				Dag3,6 uur	6	Mod3, Dag3	6	C	0	0	1	0	0
37	5	How companies work	Company practices	4	20	Dag3.Onderhoud BRZO bedrijven	Installatie beheer	Installatiebeheer op hoofdlijnen							C06B, 2uur	2					A	1	0	0	0	0
38	5	How companies work	Company practices	4	46	Dag6.1C VBS en organisatie	Indicators	Safety Performance Indicators							C10,2uur	2					A	1	0	0	0	0
39	5	How companies work	Company practices	4	27	Dag4.1A VBS en installatie	Documentatie	Bedrijfsdocumenten en Document control							C06B, 1uur	1					D	0	0	0	1	0
40	5	How companies work	Company practices	4	21	Dag4.1A VBS en installatie	BRZO bedrijven	Wat gebeurt er bij BRZO bedrijven	blok4, 1uur	1	blok4, 1 uur	1					Dag4,1uur	1	Mod4,Dag4	1	A	1	0	0	0	0
41	5	How companies work	Company practices	4	96	Ontwerp filosofie en procesinstallaties	PID PFD	Process Flow Diagrams en Process Instrumention Diagrams leren lezen.	blok4, 1uur	1	blok4, 1uur	1					Dag12, 2 uur	2	Mod7, Dag12	2	A	1	0	0	0	0
										2		5		2		7		9		9		4	1	1	1	0
42	6	Safety Management system	Appraisal	4	32	Dag5.1B VBS en onderhoud	Veilig werk	6-Beoordelen of werkzaamheden veilig gebeuren									Dag5,1/2uur	0,5	Mod4,Dag5	0,5	C	0	0	1	0	0
43	6	Safety Management system	SMS elements, Appraisal	4	25	Dag4.1A VBS en installatie	VBS7	Inspectie VBS7									Dag4,1uur	1	Mod4,Dag4	1	C	0	0	1	0	0
44	6	Safety Management system	SMS elements, Appraisal	4	47	Dag6.1C VBS en organisatie	VBS6	Inspectie VBS6									Dag6,1uur	1	Mod4,Dag6	1	C	0	0	1	0	0
45	6	Safety Management system	SMS elements, Appraisal	4	65	Dag8.2B Voldoen aan verplichtingen	VBS5	Inspectie VBS5									Dag8,1 uur	1	Mod4,Dag8	1	C	0	0	1	0	0
46	6	Safety Management system	SMS elements, Appraisal	4	44	Dag6.1C VBS en organisatie	VBS4	Inspectie VBS4									Dag6,1uur	1	Mod4,Dag6	1	C	0	0	1	0	0
47	6	Safety Management system	SMS elements, Appraisal	4	34	Dag5.1B VBS en onderhoud	VBS3	Inspectie VBS3									Dag5,1uur	1	Mod4,Dag5	1	C	0	0	1	0	0
48	6	Safety Management system	SMS elements, Appraisal	4	77	Dag9.3A Risico's inventariseren	VBS2	Inspectie VBS2									Dag9, 1 uur	1	Mod4,Dag9	1	C	0	0	1	0	0
49	6	Safety Management system	SMS elements, Appraisal	4	56	Dag7.1D VBS en werknemers	VBS1	Inspectie VBS1									Dag7,1 uur	1	Mod4,Dag7	1	C	0	0	1	0	0
50	6	Safety Management system	SMS elements	4	22	Dag4.1A VBS en installatie	VBS werking	1-Uitleggen hoe VBS elementen moeten werken									Dag4,1uur	1	Mod4,Dag4	1	C	0	0	1	0	0
51	6	Safety Management system	SMS elements, Appraisal	4	38	Dag5.1B VBS en onderhoud	Storingsanalyse	Storingsanalyse, methode en workshop	blok 6, 3 uur	3	blok 6, 3 uur	3									B	0	1	0	0	0
52	6	Safety Management system	SMS elements, Appraisal	4	41	Dag5.1B VBS en onderhoud	Safety mgmt	Huiswerk: taak SHE manager, veiligheidsmanagement									Dag5,1/2uur	0,5	Mod4,Dag5	0,5	A	1	0	0	0	0
53	6	Safety Management system	SMS elements, Appraisal	4	54	Dag7.1D VBS en werknemers	Safety communicatie	14-Beoordelen of de communicatie over veiligheid in een bedrijf goed verloopt							C11,3uur	3	Dag7,1/2uur	0,5	Mod4,Dag7	0,5	A	1	0	0	0	0
54	6	Safety Management system	SMS elements	4	24	Dag4.1A VBS en installatie	Procs	3-Beoordelen of procedures en werkinstructies voldoen aan BRZO									Dag4,1uur	1	Mod4,Dag4	1	A	1	0	0	0	0
55	6	Safety Management system	SMS elements	4	31	Dag5.1B VBS en onderhoud	PID PFD	P&ID's en PFD's									Dag5,1uur	1	Mod4,Dag5	1	A	1	0	0	0	0
56	6	Safety Management system	Safety Policy	4	23	Dag4.1A VBS en installatie	PBZO	2-Beoordelen of een PBZO document geschikt is.			blok3,1uur	1					Dag4,1uur	1	Mod4,Dag4	1	A	1	0	0	0	0
57	6	Safety Management system	SMS elements	4	30	Dag5.1B VBS en onderhoud	OH mgmt	5-Weten wat kritische factoren bij onderhoudsmanagement zijn (integriteitsmana	blok4, 1uur	1	blok4, 1uur	1					Dag5,1/2uur	0,5	Mod4,Dag5	0,5	A	1	0	0	0	0
58	6	Safety Management system	SMS elements	4	33	Dag5.1B VBS en onderhoud	OH meth & techn	7-Beoordelen van methoden en technieken bij onderhoudswerk									Dag5,1/2uur	0,5	Mod4,Dag5	0,5	A	1	0	0	0	0
59	6	Safety Management system	SMS elements	4	35	Dag5.1B VBS en onderhoud	OH meth & techn	V&G,WVG, TRA,LMRA									Dag5,1uur	1	Mod4,Dag5	1	A	1	0	0	0	0
60	6	Safety Management system	SMS elements	4	36	Dag5.1B VBS en onderhoud	OH meth & techn	WVG en toezicht							C12,2uur	2					A	1	0	0	0	0
61	6	Safety Management system	SMS elements	4	37	Dag5.1B VBS en onderhoud	OH meth & techn	TRA veiligheid studie bij hoog risico werk			blok8,1 uur	1	C09, 0.5 dag	3	C12,1 uur	1					A	1	0	0	0	0
62	6	Safety Management system	SMS elements	4	40	Dag5.1B VBS en onderhoud	OH meth & techn	Onderhoud en Risk Based Inspection			blok7, 3uur	3									A	1	0	0	0	0
63	6	Safety Management system	SMS elements	4	55	Dag7.1D VBS en werknemers	Instructies	instructies geven en opschrijven									Dag7,1/2uur	0,5	Mod4,Dag7	0,5	C	0	0	1	0	0
64	6	Safety Management system	SMS elements	4	58	Dag7.1D VBS en werknemers	Instructies	Huiswerk: taal&veiligheid e-learning, inlezen BRZO regelgeving									Dag7,1/2uur	0,5	Mod4,Dag7	0,5	C	0	0	1	0	0
65	6	Safety Management system	Appraisal	4	45	Dag6.1C VBS en organisatie	Indicators	10-Beoordelen of bedrijfs-KPI's voldoende over veiligheid zeggen									Dag6,1uur	1	Mod4,Dag6	1	C	0	0	1	0	0
66	6	Safety Management system	SMS elements	4	48	Dag6.1C VBS en organisatie	Human factors	Huiswerk: orientatie op human factors, zorgplicht, 2 artikelen over jurisprudentie									Dag6,1uur	1	Mod4,Dag6	1	A	1	0	0	0	0
67	6	Safety Management system	Appraisal	4	49	Dag7.1D VBS en werknemers	Human factors	11-Beoordelen van de rol van human factors in een bedrijf	blok7, 2uur	2	blok8,3uur	3			C11,1 uur	1					A	1	0	0	0	0
68	6	Safety Management system	SMS elements	4	50	Dag7.1D VBS en werknemers	Human factors	Functieomschrijving en opleidingsmatrix									Dag7,1/2uur	0,5	Mod4,Dag7	0,5	A	1	0	0	0	0
69	6	Safety Management system	SMS elements	4	51	Dag7.1D VBS en werknemers	Human error	12-Uitleggen wat menselijk falen is en wat de oorzaken daarvan zijn							C08B, 1uur	1	Dag7,1/2uur	0,5	Mod4,Dag7	0,5	A	1	0	0	0	0
70	6	Safety Management system	SMS elements	4	52	Dag7.1D VBS en werknemers	Cultuur	13-Inschatten (globaal) van de veiligheidscultuur van een organisatie					C10, 1 dag	6	C11,2uur	2	Dag7,1/2uur	0,5	Mod4,Dag7	0,5	A	1	0	0	0	0
71	6	Safety Management system	SMS elements	4	53	Dag7.1D VBS en werknemers	Cultuur	Nudges, gedragsturing	blok7, 1uur	1	blok8,2uur	2					Dag7,1 uur	1	Mod4,Dag7	1	A	1	0	0	0	0
72	6	Safety Management system	SMS elements	4	39	Dag5.1B VBS en onderhoud	Contractors	Contractorveiligheid	blok7, 1uur	1	blok8, 3 uur	3	C09, 0.5 dag	3	C12,1 uur	1					A	1	0	0	0	0
73	6	Safety Management system	SMS elements	4	43	Dag6.1C VBS en organisatie	Change mgmt	9-Uitleggen hoe change management werkt									Dag6,1uur	1	Mod4,Dag6	1	A	1	0	0	0	0
74	6	Safety Management system	Safety policy	4	64	Dag8.2B Voldoen aan verplichtingen	Trends	Trends in veiligheid									Dag8,1/2uur	0,5	Mod5,Dag8	0,5	C	0	0	1	0	0
75	6	Safety Management system	Appraisal	4	63	Dag8.2B Voldoen aan verplichtingen	Instructies	Beoordelen van een procedure of werkinstructie									Dag8,1/2uur	0,5	Mod5,Dag8	0,5	C	0	0	1	0	0
76	6	Safety Management system	Appraisal	4	62	Dag8.2B Voldoen aan verplichtingen	Arbo aspecten	16-Beoordelen of de bepalingen en uitgangspunten van de Arbowetgeving zijn to									Dag8,1/2uur	0,5	Mod5,Dag8	0,5	A	1	0	0	0	0
77	6	Safety Management system	SMS elements, risk mgmt	4	79	Dag10.3B Op risico beheersing inspecteren	Risk management	22-Uitleggen wat risicobeheersing met een VBS inhoudt	blok7, 3 uur	3							Dag10,1/2 uur	0,5	Mod6,Dag10	0,5	A	1	0	0	0	0
78	6	Safety Management system	SMS elements, risk mgmt	4	75	Dag9.3A Risico's inventariseren	Risk assessment systema	20-Beoordelen of risico's voldoende systematisch geinventariseerd zijn									Dag9, 1/2 uur	0,5	Mod6,Dag9	0,5	A	1	0	0	0	0
79	6	Safety Management system	SMS elements, risk mgmt	4	69	Dag9.3A Risico's inventariseren	Risk assessment swot	18-Uitleggen welke sterktes en zwaktes diverse risico analyse methoden hebben									Dag9, 1/2 uur	0,5	Mod6,Dag9	0,5	A	1	0	0	0	0
80	6	Safety Management system	SMS elements, risk mgmt	4	70	Dag9.3A Risico's inventariseren	Risk assessment method	Veel gebruikte risico analyse methoden									Dag9, 1/2 uur	0,5	Mod6,Dag9	0,5	A	1	0	0	0	0
81	6	Safety Management system	SMS elements, risk mgmt	4	71	Dag9.3A Risico's inventariseren	Risk assessment invento	Risico inventarisatie									Dag9, 1 uur	1	Mod6,Dag9	1	A	1	0	0	0	0
82	6	Safety Management system	SMS elements, risk mgmt	4	72	Dag9.3A Risico's inventariseren	Risk assessment beoorde	19-Beoordelen welke risico analyse methoden voor een bedrijf geschikt zijn									Dag9, 1/2 uur	0,5	Mod6,Dag9	0,5	A	1	0	0	0	0
83	6	Safety Management system	SMS elements, risk mgmt	4	76	Dag9.3A Risico's inventariseren	Risk assessment beoorde	21-Beoordelen of de risicoanalyse aan BRZO voldoet	blok7, 3 uur	3							Dag9, 1/2 uur	0,5	Mod6,Dag9	0,5	A	1	0	0	0	0
84	6	Safety Management system	SMS elements, risk mgmt	4	68	Dag8.2B Voldoen aan verplichtingen	Risk assessment	Huiswerk: risico inventarisatie- en analyse technieken									Dag8,1/2uur	0,5	Mod6,Dag8	0,5	A	1	0	0	0	0
85	6	Safety Management system	SMS elements, risk mgmt	4	73	Dag9.3A Risico's inventariseren	Risico matrix en scenario	Risico matrix en scenario									Dag9, 1 uur	1	Mod6,Dag9	1	A	1	0	0	0	0
86	6	Safety Management system	SMS elements, risk mgmt	4	84	Dag10.3B Op risico beheersing inspecteren	Risico matrix beoordelen	Beoordelen risicomatrix, wat kan een bedrijf zoal fout doen									Dag10,1/2 uur	0,5	Mod6,Dag10	0,5	A	1	0	0	0	0
87	6	Safety Management system	SMS elements, risk mgmt	4	83	Dag10.3B Op risico beheersing inspecteren	Risico matrix	23-Uitleggen hoe een risicomatrix werkt									Dag10,1/2 uur	0,5	Mod6,Dag10	0,5	A	1	0	0	0	0
88	6	Safety Management system	SMS elements, risk mgmt	4	74	Dag9.3A Risico's inventariseren	Risico criteria	Risicobepaling en criteria							C09, 1uur	1					A	1	0	0	0	0
89	6	Safety Management system	SMS elements, risk mgmt	4	85	Dag10.3B Op risico beheersing inspecteren	Risico beheersing	24-Beoordelen of risico's voldoende zijn beheerst volgens BRZO									Dag10,1/2 uur	0,5	Mod6,Dag10	0,5	A	1	0	0	0	0
90	6	Safety Management system	SMS elements, risk mgmt	4	78	Dag9.3A Risico's inventariseren	Restrisico	Huiswerk: restrisico, scenario schrijven, artikel unknown risk									Dag9, 1/2 uur	0,5	Mod6,Dag9	0,5	A	1	0	0	0	0
91	6	Safety Management system	SMS elements, risk mgmt	4	81	Dag10.3B Op risico beheersing inspecteren	PDCA	PDCA en het VBS									Dag10,1/2 uur	0,5	Mod6,Dag10	0,5	A	1	0	0	0	0
92	6	Safety Management system	SMS elements	4	151	Evaluatie en afsluiting	Evaluatie en Afsluiting	Evaluatie en Afsluiting							C12,2 uur	2					D	0	0	0	1	0
93	6	Safety Management system	SMS elements, risk mgmt	4	131	Procesveiligheid	Risk assessment	Risico analyse + casus (Hazop)	blok5, 1 uur	1	blok5, 1 uur	1							Mod8, Dag18	3	A	1	0	0	0	0
94	6	Safety Management system	SMS elements, risk mgmt	4	135	Risico herkennen in de praktijk	Risico	risicos in praktijk (Hazop beoordelen)									Dag18, 6uur	6	Mod8, Dag18	3	A	1	0	0	0	0
95	6	Safety Management system	SMS elements, Appraisal	4	42	Dag6.1C VBS en organisatie	Safety mgmt	8-Uitleggen hoe veiligheidsmanagement in een organisatie werkt	blok7, 3uur	3							Dag6,1uur	1	Mod4,Dag6	1	A	1	0	0	0	0
96	6	Safety Management system	SMS elements	4	57	Dag7.1D VBS en werknemers	Agenda opstellen	Opstellen van gezamenlijke inspectie agenda									Dag7,1 uur	1	Mod4,Dag7	1	C	0	0	1	0	0
97	6	Safety Management system	SMS elements	4	26	Dag4.1A VBS en installatie	Degradatie	Huiswerk: Artikel badkuipkromme, Artikel over losses oil & gas									Dag4,1uur	1	Mod4,Dag4	1	C	0	0	1	0	0
98	6	Safety Management system	SMS elements	4	28	Dag5.1B VBS en onderhoud	Degradatie	4-Uitleggen welke verouderingsmechanismen in installatie en organisatie een rc									Dag5,1/2uur	0,5	Mod4,Dag5	0,5	C	0	0	1	0	0
99	6	Safety Management system	SMS elements	4	29	Dag5.1B VBS en onderhoud	Degradatie	Ageing, Badkuipkromme									Dag5,1uur	1	Mod4,Dag5	1	C	0	0	1	0	0
										18		18		12		14		41		41		38	1	18	1	0
100	7	Process installations	Design	4	90	Ontwerp filosofie en procesinstallaties	Theorie reacties	Theorie Reactiekinetiek							C07A,2uur	2					A	1	0	0	0	0
101	7	Process installations	Design	4	111	Ontwerp filosofie en procesinstallaties	Theorie reacties	Reactoren + chemische reacties, runaways, fysische processen.	blok4, 1uur	1	blok4, 2uur	2					Dag15, 2 uur	2	Mod7, Dag15	2	A	1	0	0	0	0
102	7	Process installations	Technical safety	4	134	Procesveiligheid	Theorie PVA SIL	SIL Safety Integrity Level, eenvoudige berekeningen	blok7, 5 uur	5	blok7, 3 uur	3	C01, 2 dagen	12					Mod7, Dag16	2	A	1	0	0	0	0
103	7	Process installations	Technical safety	4	126	Procesveiligheid	Theorie PVA Markov	Markov processen	blok5, 2 uur	2	blok5, 2 uur	2									E	0	0	0	0	1
104	7	Process installations	Technical safety	4	121	Procesveiligheid	Theorie PVA Kansen	Berekenen PFD foutkans							C08B,5uur	5					E	0	0	0	0	1
105	7	Process installations	Technical safety	4	122	Procesveiligheid	Theorie PVA Kansen	Kansrekening, Risico analyse, gevarenbronnen, Scenario + casus							C08A,5uur	5			Mod7, Dag16	2	E	0	0	0	0	1
106	7	Process installations	Technical safety	4	129	Procesveiligheid	Theorie PVA Kansen	Kansaspecten							C09, 1 uur	1					E	0	0	0	0	1
107	7	Process installations	Technical safety	4	127	Procesveiligheid	Theorie PVA Human erro	Analyse aan menselijk falen	blok5, 1 uur	1	blok5, 1 uur	1			C09, 1 uur	1					E	0	0	0	0	1
108	7	Process installations	Technical safety	4	124	Procesveiligheid	Theorie PVA Fault tree	Foutenbomen	blok5, 2 uur	2	blok5, 2 uur	2									E	0	0	0	0	1
109	7	Process installations	Technical safety	4	125	Procesveiligheid	Theorie PVA Fault tree	Kwantificeren foutenbomen	blok5, 6 uur	6	blok5, 6 uur	6									E	0	0	0	0	1
110	7	Process installations	Technical safety	4	123	Procesveiligheid	Theorie PVA Faaldata	Kansen, reliability, faaldata, RAMS	blok5, 4 uur	4	blok5, 4 uur	4							Mod7, Dag16	2	E	0	0	0	0	1
111	7	Process installations	Technical safety	4	128	Procesveiligheid	Theorie PVA Event tree	Gebeurtenissenboom analyse	blok5, 2 uur	2	blok5, 2 uur	2									E	0	0	0	0	1
112	7	Process installations	Technical safety	4	119	Procesveiligheid	Theorie PVA	Procesv.anal., HAZOP, FMECA,FTA, Plant lay-out,QRA					C07, 3 dagen	18					Mod7, Dag17	2	E	0	0	0	0	1
113	7	Process installations	Technical safety	4	120	Procesveiligheid	Theorie PVA	Procesveiligheidsanalyse, robuustheid	blok6, 3 uur	3	blok6, 3 uur	3	C08, 3 dagen	18					Mod7, Dag17	2	E	0	0	0	0	1
114	7	Process installations	Design	4	88	Ontwerp filosofie en procesinstallaties	Theorie processen	Theorie Enkele processen, oa kolenvergassing	blok4, 1uur	1	blok4, 1uur	1									A	1	0	0	0	0
115	7	Process installations	Design	4	93	Ontwerp filosofie en procesinstallaties	Theorie processen	Procesoptimalisatie							C07B,2uur	2					A	1	0	0	0	0
116	7	Process installations	Design	4	94	Ontwerp filosofie en procesinstallaties	Theorie processen	Proceswijziging Methanolsynthese casus							C07B,1uur	1					A	1	0	0	0	0
117	7	Process installations	Design	4	106	Ontwerp filosofie en procesinstallaties	Theorie processen	Inleiding processen							C07A,2uur	2					A	1	0	0	0	0
118	7	Process installations	Design	4	107	Ontwerp filosofie en procesinstallaties	Theorie processen	Bioprocestechnologie							C07A,1uur	1					A	1	0	0	0	0
119	7	Process installations	Design	4	112	Ontwerp filosofie en procesinstallaties	Theorie processen	Principes van destilleren, extraheren, verdampen, centrifugeren.	blok4, 1uur	1							Dag15, 2 uur	2	Mod7, Dag15	4	A	1	0	0	0	0
120	7	Process installations	Design	4	91	Ontwerp filosofie en procesinstallaties	Theorie ontwerp	Ontwerp filosofieën									Dag11, 2 uur	2	Mod7, Dag11	2	A	1	0	0	0	0
121	7	Process installations	Design, Technical safety	4	92	Ontwerp filosofie en procesinstallaties	Theorie ontwerp	Introductie in ontwerpfilosofieën (wat is; inherent veilig, fail safe, safety layer).	blok6, 6 uur	6	blok6, 6 uur	6					Dag11, 2 uur	2	Mod7, Dag11	2	A	1	0	0	0	0
122	7	Process installations	Design	4	89	Ontwerp filosofie en procesinstallaties	Theorie installaties	Theorie Inleiding Procesindustrie, start, stop, samenwerken					C06, 2 dagen	12	C07A,1uur	1			Mod7, Dag17	2	A	1	0	0	0	0
123	7	Process installations	Design, Technical safety	4	95	Ontwerp filosofie en procesinstallaties	Theorie installaties	Storingsgevoeligheid van installaties/componenten.									Dag11, 2 uur	2	Mod7, Dag11	2	A	1	0	0	0	0
124	7	Process installations	Design	4	98	Ontwerp filosofie en procesinstallaties	Theorie installaties	Basic process control system (proces regelingen).									Dag12, 2 uur	2	Mod7,Dag12	2	A	1	0	0	0	0
125	7	Process installations	Design	4	100	Ontwerp filosofie en procesinstallaties	Theorie installaties	Regelsystemen (PLC, Software, bus, communicatie, internet, MES, ERP)							C10, 1uur	1	Dag13, 2 uur	2	Mod7, Dag13	2	A	1	0	0	0	0
126	7	Process installations	Design	4	108	Ontwerp filosofie en procesinstallaties	Theorie installaties	Reactoren							C07B,1uur	1					A	1	0	0	0	0
127	7	Process installations	Design	4	109	Ontwerp filosofie en procesinstallaties	Theorie installaties	Unit operations							C07B,2uur	2					A	1	0	0	0	0
128	7	Process installations	Design	4	110	Ontwerp filosofie en procesinstallaties	Theorie installaties	Batchreactoren + chemische reacties.	blok4, 1uur	1			D01, + 2 dagen	12			Dag15, 2 uur	2			A	1	0	0	0	0
129	7	Process installations	Design	4	113	Ontwerp filosofie en procesinstallaties	Theorie installaties	Dampverwerkingsinstallaties.									Dag16, 2 uur	2			A	1	0	0	0	0
130	7	Process installations	Design	4	114	Ontwerp filosofie en procesinstallaties	Theorie installaties	Destillatietorens.									Dag16, 2 uur	2			A	1	0	0	0	0
131	7	Process installations	Design	4	115	Ontwerp filosofie en procesinstallaties	Theorie installaties	Warmtewisselaars.									Dag16, 2 uur	2			A	1	0	0	0	0
132	7	Process installations	Design	4	116	Ontwerp filosofie en procesinstallaties	Theorie installaties	Flares.									Dag17, 2 uur	2			A	1	0	0	0	0
133	7	Process installations	Design	4	117	Ontwerp filosofie en procesinstallaties	Theorie installaties	Laad- en losplaatsen van tankauto's.									Dag17, 2 uur	2			A	1	0	0	0	0
134	7	Process installations	Design, Technical safety	4	99	Ontwerp filosofie en procesinstallaties	Theorie beveiligingen	Operator monitoring, controlling and acting (normaal, nood)									Dag13, 2 uur	2	Mod7, Dag13	2	A	1	0	0	0	0
135	7	Process installations	Design, Technical safety	4	101	Ontwerp filosofie en procesinstallaties	Theorie beveiligingen	Instrumentele beveiligingen.									Dag13, 2 uur	2	Mod7, Dag13	2	A	1	0	0	0	0
136	7	Process installations	Design, Technical safety	4	102	Ontwerp filosofie en procesinstallaties	Theorie beveiligingen	Mechanische beveiligingen, valkuilen in praktijk									Dag14, 2 uur	2	Mod7, Dag14	2	A	1	0	0	0	0
137	7	Process installations	Design, Technical safety	4	103	Ontwerp filosofie en procesinstallaties	Theorie beveiligingen	Instrumentele beveiligingssystemen (DCS & PLC).									Dag14, 2 uur	2	Mod7, Dag14	2	A	1	0	0	0	0
138	7	Process installations	Design	4	87	Ontwerp filosofie en procesinstallaties	Theorie balansen	Theorie Massa, energie en economische balansen	blok4, 1uur	1	blok4, 1uur	1									B	0	1	0	0	0
139	7	Process installations	Design, Technical safety	4	104	Ontwerp filosofie en procesinstallaties	Tanks	Tank oplag (EEMUA 159/183, PGS 29 en PGS 30), ontwerpcodes, normen					C05, 1 dag	6			Dag14, 2 uur	2	Mod7, Dag14	2	A	1	0	0	0	0
140	7	Process installations	Design, Technical safety	4	105	Ontwerp filosofie en procesinstallaties	Tanks	Tankopslag					D02, + 2 dagen	12							A	1	0	0	0	0
141	7	Process installations	Design	4	132	Procesveiligheid	Layout	Layout, areaclassificatie	blok6, 3 uur	3	blok6, 3 uur	3									B	0	1	0	0	0
142	7	Process installations	Design	4	133	Procesveiligheid	Layout	Atex en Verblijfsgebouwen					D03, + 1 dag	6							B	0	1	0	0	0
143	7	Process installations	Design, Technical safety	4	97	Ontwerp filosofie en procesinstallaties	Degradatie	Materiaalkunde en corrosie/erosie, monitoring, theorie en praktijk	blok4, 1uur	1	blok4, 1uur	1			C10,2uur	2	Dag12, 2 uur	2	Mod7, Dag12	2	C	0	0	1	0	0
144	7	General legislation	ATEX cursus	3	6	occupational safety	ARBO ATEX	Theorie, zonering, maatregelen, EVD, normen					C02,3 dagen	18			12 uur	12	Mod10, 12 uur	12	C	0	0	1	0	0
145	7	General legislation	ATEX proefinspectie	3	6	occupational safety	ARBO ATEX	Atex inspectie in praktijk met begeleiding (ICT)									6 uur	6	Mod10, 6 uur	6	C	0	0	1	0	0
146	7	General legislation	Pressure	3	6	occupational safety	ARBO DA-PED	Drukapparatuur/PED					C03, 2dagen	12			12 uur	12	Mod11, 12 uur	12	C	0	0	1	0	0
										40		37		126		27		68		70		29	3	4	0	11
147	8	Dangerous chemicals	Labels, legislation	3	6	occupational safety	ARBO REACH - CLP	REACH - CLP					AOG11, 1 dag	6			Reach-CLP	3			C	0	0	1	0	0
148	8	Dangerous chemicals	Hazards	3	6	occupational safety	ARBO GS	Gevaarlijke stoffen									GS	3			C	0	0	1	0	0
149	8	Dangerous chemicals	Legislation/Standards	4	10	Dag1.Introductie BRZO	AGS	Adviesraad Gevaarlijke Stoffen	blok7, 1 uur	1											B	0	1	0	0	0
										1		0		6		0		6		0		0	1	2	0	0
150	9	Learning from accidents	Investigation methods	3	4	law enforcement skills	ARBO OOB	Ongeval Onderzoek Basis					A06, 1/3 dag	2							D	0	0	0	1	0
151	9	Learning from accidents	Investigation methods	4	143	Leren van ongevallen	Stilleggen	Stilleggen : hoe en wanneer ?; weer vrijgeven : hoe en wanneer ?									Dag20, 2uur	2			D	0	0	0	1	0
152	9	Learning from accidents	Accident history	4	130	Procesveiligheid	Ongevallen	Effecten van ongevallen							C09, 1 uur	1					A	1	0	0	0	0
153	9	Learning from accidents	Accident history	4	136	Leren van ongevallen	Ongevallen	Ongevallen (Arbo, Zware ongevallen) in Nederland en internationaal	blok7, 1 uur	1							Dag19, 2uur	2	Mod9, Dag19	2	A	1	0	0	0	0
154	9	Learning from accidents	Accident history	4	137	Leren van ongevallen	Ongevallen	Ongevallen in de industrie en hun oorzaken							C06A, 1 uur	1			Mod9, Dag20	2	A	1	0	0	0	0
155	9	Learning from accidents	Records & Systems	4	138	Leren van ongevallen	Ongevallen	MARS ongevallen	blok7, 1 uur	1											A	1	0	0	0	0
156	9	Learning from accidents	Investigation methods	4	139	Leren van ongevallen	Ongevallen	Ongeval onderzoek MHC			blok3, 2uur	2	C011, (pm)				Dag19, 2uur	2	Mod9, Dag19	2	A	1	0	0	0	0
157	9	Learning from accidents	Records & Systems	4	140	Leren van ongevallen	Ongevallen	Story builder Analyse MHC ongevallen							C06A, 1 uur	1					A	1	0	0	0	0
158	9	Learning from accidents	Investigation methods	4	141	Leren van ongevallen	Ongevallen	Ongeval onderzoek methodiek							C06A, 1 uur	1			Mod9, Dag19	2	A	1	0	0	0	0
159	9	Learning from accidents	Records & Systems	4	142	Leren van ongevallen	Ongevallen	Hoe is ongeval onderzoek in Nederland geregeld (Arbo, MHC, OM, OVV, RIVM, M									Dag19, 2uur	2	Mod9, Dag20	2	A	1	0	0	0	0
160	9	Learning from accidents	Accident history	4	144	Leren van ongevallen	Casuistiek	Casuistiek: diverse historische cases	blok1, 2uur	2							Dag20, 2uur	2	Mod9, Dag20	2	A	1	0	0	0	0
161	9	Learning from accidents	Accident history	4	145	Leren van ongevallen	Casuistiek	Casuistiek: Kemira case	blok4, 6 uur	6	blok4, 6 uur	6									A	1	0	0	0	0
162	9	Learning from accidents	Accident history	4	146	Leren van ongevallen	Casuistiek	Casuistiek: Enschede case	blok7, 1 uur	1											A	1	0	0	0	0
163	9	Learning from accidents	Accident history	4	147	Leren van ongevallen	Casuistiek	Casuistiek: ChemiePak case							C06B, 2 uur	2					A	1	0	0	0	0
164	9	Learning from accidents	Accident history	4	148	Leren van ongevallen	Casuistiek	Casuistiek: Explosie NAK Geleen							C10,1uur	1					A	1	0	0	0	0
165	9	Learning from accidents	Accident history	4	149	Leren van ongevallen	Casuistiek	Bespreken CSB video's.									Dag20, 2uur	2			A	1	0	0	0	0
										11		8		2		7		12		12		14	0	0	2	0
166	10	Learning from practice	Inspection skills	3	5	General skills	SZW RijV	Rijvaardigheidstraining									[1dag]	6			C	0	0	1	0	0
167	10	Learning from practice	Inspection skills	3	4	law enforcement skills	MHC VHT	Verhoortechnieken																		

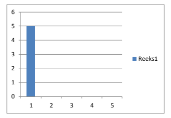

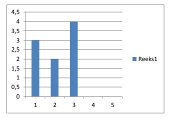

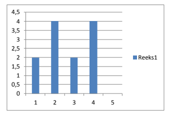

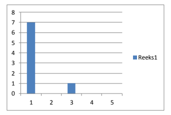

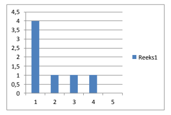

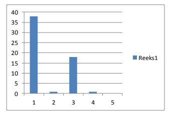

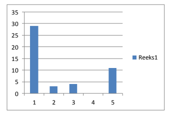

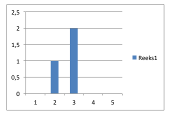

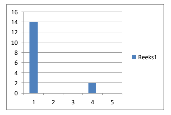

SUMMARY OF HOURS PER ETP BLUE PRINT NR

Nr	Aspect	Hours spent on education and training per inspector per aspect per time section 2003	2008	2014	2018
ETP Subject matter					
1	Job Requirements	0	0	0	0
2	General legislation	141	141	210	210
3	Special Legislation	10	10	2	3,5
4	Cooperation	7	13	15	23
5	How companies work	2	5	7	9
6	Safety Management System	18	18	14	41
7	Process installations	40	37	27	68
8	Dangerous chemicals	1	0	0	6
9	Learning from accidents	11	6	7	12
10	Learning from practice	79	69	110	126,5
11	Coaching	10	6	9	15

168	10	Learning from practice	Inspection skills	3	5	General skills	MHC TIV	Training interventie vaardigheden	TIV	36	TIV	36	TIV-MHC, 6 dagen	36	TIV	36	TIV	36			A	1	0	0	0	0
169	10	Learning from practice	Inspection skills	3	4	law enforcement skills	MHC PPV	Practicum proces-verbaal MHC	PPV	18	PPV	18	B06, 3 dagen	18	PPV	18	PPV	18			A	1	0	0	0	0
170	10	Learning from practice	Inspection skills	3	4	law enforcement skills	MHC PBR	Practicum boeterapport							PBR (?)	18	PBR (?)	18			C	0	0	1	0	0
171	10	Learning from practice	Inspection skills	3	4	law enforcement skills	MHC HAND	Handhaving BRZO 2015					B04, 2 dagen	12	HAND	12	HAND	12			C	0	0	1	0	0
172	10	Learning from practice	Inspection skills	4	60	Dag8.2B Voldoen aan verplichtingen	Visuele inspectie	Algemene indrukken, waar let je op in een bedrijf?									Dag8,1/2uur	0,5	Mod5,Dag8	0,5	C	0	0	1	0	0
173	10	Learning from practice	Inspection skills	4	59	Dag8.2B Voldoen aan verplichtingen	Maatregelen	15-Analyseren en beoordelen van de (genomen) maatregelen van een bedrijf									Dag8,1/2uur	0,5	Mod5,Dag8	0,5	C	0	0	1	0	0
174	10	Learning from practice	Inspection skills	4	66	Dag8.2B Voldoen aan verplichtingen	Instrumenten vd inspecte	Instrumenten voor de inspecteur en daar uit kiezen									Dag8,1 uur	1	Mod5,Dag8	1	C	0	0	1	0	0
175	10	Learning from practice	Inspection skills	4	67	Dag8.2B Voldoen aan verplichtingen	Instrumenten vd inspecte	17-Bepalen of handhaving of discretionaire bevoegdheid nodig is									Dag8,1/2uur	0,5	Mod5,Dag8	0,5	C	0	0	1	0	0
176	10	Learning from practice	Inspection skills	4	86	Dag10.3B Op risico beheersing inspecteren	Toetsing	BOP Toetsing met vragen behandelen voor een panel									Dag10, 2 uur	2	Mod6,Dag10	2	C	0	0	1	0	0
177	10	Learning from practice	Inspection skills	4	80	Dag10.3B Op risico beheersing inspecteren	Maatregelen	Maatregelgerichte inspectie									Dag10,1 uur	1	Mod6,Dag10	1	C	0	0	1	0	0
178	10	Learning from practice	Inspection skills	4	82	Dag10.3B Op risico beheersing inspecteren	Maatregelen	Maatregelen en interactie met het ontwerp							C09, 1uur	1					D	0	0	0	1	0
179	10	Learning from practice	Test-site	4	150	Proeffabriek	Proeffabriek	Oefening te Brielle									Dag21, 6 uur	6	Mod12, 12 uur	12	C	0	0	1	0	0
180	10	Learning from practice	Inspection skills	5	1	Inspecteursopleiding LAT/BRZO+	NIVRIM	Inspectiemethodiek	blok3, 6 uur	6											B	0	1	0	0	0
181	10	Learning from practice	Inspection skills	5	2	Inspecteursopleiding LAT/BRZO+	AVRIM2	Inspectiemethodiek	blok3, 6 uur	6											B	0	1	0	0	0
182	10	Learning from practice	Inspection skills	5	3	Inspecteursopleiding LAT/BRZO+	NIM	Inspectiemethodiek			blok3, 2 uur	2	A07, 2 dagen	12	NIM	12	NIM	12			C	0	0	1	0	0
183	10	Learning from practice	Inspection skills	5	4	Inspecteursopleiding LAT/BRZO+	NIM Inspectieleider	Taak en rol inspectieleider					3 dagen	18							C	0	0	1	0	0
184	10	Learning from practice	Joint inspections	7	3	Permanente her- en bijscholing	Maatjessysteem	Meelopen met ervaren collega in praktijk	1 year maatjes systeem	0	-	0	yes	0	joint yearly cross-team inspection	0	-	0			A	1	0	0	0	0
185	10	Learning from practice	Inspection skills	7	6	Permanente her- en bijscholing	PVI Herhalingsmodules	Procesveiligheid en inspectiepraktijk	yes	10	yes	10	PVI,+ 6 dagen / 4 jaar	10	yes	10	yes	10			A	1	0	0	0	0
186	10	Learning from practice	Inspection skills	7	7	Permanente her- en bijscholing	TIV-P Herhaling	TIV pitstop bijscholing interventievaardigheden	yes	3	yes	3	TIV-P, 2 dagen/5 jaar	3	yes	3	yes	3			A	1	0	0	0	0
187	10	Learning from practice	Joint inspections	7	8	Permanente her- en bijscholing	Mutual Joint EU visits	Uitwisseling Seveso ervaringen tussen EU landen	1p/jaar	0	1p/jaar	0		0	1p/jaar	0	1p/jaar	0			A	1	0	0	0	0
										79		69		109		110		126,5		17,5		6	2	12	1	0
188	11	Coaching	Support facilities	3	1	Centrale introductie I-SZW	SZW INTRO	Introductie Inspectie SZW	yes	6	yes	6	A01, 1 dag	6							A	1	0	0	0	0
189	11	Coaching	Support facilities	3	2	support facilities	SZW I-net	basistraining (GISAI, I-net, . . .)					A02, 1 dag	6	i-net	6	i-net	6			A	1	0	0	0	0
190	11	Coaching	Support facilities	3	2	support facilities	SZW FIMO	SZW administrative routines							C08,3 uur	3	FIMO	3			A	1	0	0	0	0
191	11	Coaching	Support facilities	3	2	support facilities	SZW DIWI	Database BRZO, DIWA, DIWI	blok1, 1 uur	1											A	1	0	0	0	0
192	11	Coaching	KMS Work instructions	3	2	support facilities	MHC KMS	kwaliteitsmgmt systeem MHC processen	blok1, 3 uur	3	blok1, 3 uur						KMS	6	Mod13, 6 uur	6	A	1	0	0	0	0
193	11	Coaching	3rd party training courses	7	1	Permanente her- en bijscholing	Diverse ad-hoc cursusser	maintain knowledge	yes		yes		yes		yes		yes				A	1	0	0	0	0
194	11	Coaching	Specialist knowledge	7	2	Permanente her- en bijscholing	MHC dagen landelijk	keep up with new developments	yes		yes		yes		yes		yes				A	1	0	0	0	0
195	11	Coaching	Team-meeting	7	4	Permanente her- en bijscholing	ICT - Intercollegiale toets	Team overleg m.b.t. concrete gevallen	Elke 14 dagen		Elke 14 dagen				Elke 14 dagen		Elke 14 dagen				A	1	0	0	0	0
196	11	Coaching	Specialist knowledge	7	5	Permanente her- en bijscholing	Coaching	6 wekelijks overleg	-		-		-		-		yes				C	0	0	1	0	0
										10		6		12		9		15		6		8	0	1	0	0
																						116				
		CONTEXT																								
197	12	Safety inspection approach	Scope, frequency			Safety Inspection Approach		Scope	discussion		GIR record & planning				GIR record & planning		GIR record & planning									
198	12	Safety inspection approach	Focus, attitude			Safety Inspection Approach		Focus	risk based		risk based				risk based		risk based				total	224	28	89	18	22
199	12	Safety inspection approach	Access to information			Safety Inspection Approach		Access to information	SZW Library		SZW Library				SZW Library, DIWA		SZW Library, DIWI									
200	12	Safety inspection approach	Inspection method			Background info		Inspection method	AVRIM-2		NIVRIM				NIM 2.0		NIM 2.0 [LBR]									
										0		0		0		0		0		0						
201	13	Reporting system	Uniformity			Cooperation with Partners		inspection info exchange	e-mail traffic		GIR ICT platform				GIR ICT platform		GIR ICT platform									
202	13	Reporting system	Content			Cooperation with Partners		Inspection reporting	Nietjesvariant		waarnemingen, bevindingen, conclu				waarnemingen, bevindingen, conclusies, b		waarnemingen, bevindingen, conclusies									
203	13	Reporting system	Writers			Reporting System		Writers	SZW officer		SZW officer				SZW officer		SZW officer									
204	13	Reporting system	Content			Reporting System		Content	EU level MARS report		EU level MARS report				EU level MARS report		EU level MARS report									
205	13	Reporting system	Uniformity			Reporting System		Uniformity	Occupational accidents system		Occupational accidents system				Occupational accidents system		Occupational accidents system									
206	13	Reporting system	Public/proprietary			Reporting System		Public/Proprietary	RIVM, TNO, OVV		RIVM, TNO, OVV				RIVM, TNO, OVV		RIVM, TNO, OVV									
207	13	Reporting system	Uniformity			Reporting System		Uniformity/joint inspection administration	-		GIR system				GIR system		GIR system									
208	13	Reporting system	Public/proprietary			Public Information		Inspection results	-		-				-		Summary reports									
209	13	Reporting system	Public/proprietary			Public Information		ranking results	-		-				ranking lijst 2e kamer		-									
210	13	Reporting system	Public/proprietary			Public Information		law enforcement results	-		-				-		-									
										0		0		0		0		0		0						
211	14	Appraisal/ranking	Parameters			Appraisal/Ranking		Parameters	-		-				-		-									
212	14	Appraisal/ranking	Appraisal criteria			Appraisal/Ranking		criteria VBS status	-		NIM levels				NIM levels		NIM levels									
213	14	Appraisal/ranking	Ranking method			Appraisal/Ranking		criteria Company status	-		-				-		NIM, levels, LBR status									
										0		0		0		0		0		0						
214	15	Cooperation	Joint GIR platform	5	6	Inspecteursopleiding LAT/BRZO+	GIR	Openbare samenvatting					A07, 1 dag	6	yes		yes									
215	15	Public information	Inspection summary			-		-	-		-				-		public summary format in use									
216	15	Public information	Inspection results			-		-	-		-				-		-									
217	15	Public information	Ranking results			-		-	-		-				-		-									
218	15	Public information	Law enforcement results			-		-	-		-				-		-									
219	15	Public information	Transparency			-		-	-		-				-		-									
										0		0		6		0		0		0						
220	16	Negotiations	Standards			Standards		Safety Management Standards	ISO 9000, ISO 14000, OHSAS 18000, (		ISO 9000, ISO 14000, OHSAS 18000,				ISO 9000, ISO 14000, OHSAS 18000, NTA 8		ISO 9000, ISO 14000, OHSAS 18000, NT									
221	16	Negotiations	Public-Private Network			Learning from practice		Company visits	-		-				-		-									
222	16	Negotiations	Public-Private Network			Learning from practice		International meetings	yearly Joint visits in EU		yearly Joint visits in EU				yearly Joint visits in EU		yearly Joint visits in EU									
223	16	Negociations	Standards			Negotiations with industry		Technical Standards	CPR Working groups		PGS Working groups				PGS Working groups		PGS Working groups									
										0		0		0		0		0		0						
224	17	Law enforcement	Legislation			General legislation		Legal Action	Wed, Arbowet 1998, BRZO99		Wed, Arbowet, BRZO99				Wed, Arbowet, BRZO99, WAHSS		Wed, Arbowet, WAHSS, BRZO2015									
225	17	Law enforcement	Legislation			Special Legislation		Dutch legislation	BRZO1999, RRZO 1999, AVR		BRZO1999, RRZO 1999, ARIE				BRZO1999, RRZO 1999		BRZO 2015, RRZO 2015									
226	17	Law enforcement	Tools			Law Enforcement		Tools	Stil, Waarsch, Eis, PV		Stil, Waarsch, Eis, PV				Stil, Eis, PV, Boete		Stil, Eis, PV, Boete									
227	17	Law enforcement	Activity stops			Law Enforcement		Activity Stops	Stillegging (gevaar)		Stillegging (gevaar)				Stillegging (gevaar/straf)		Stillegging (gevaar/straf)									
228	17	Law enforcement	Licence to operate			Law Enforcement		Licence to Operate	[bij Wm]		[bij Wm]				[bij Wabo]		[bij Wabo]									
229	17	Law enforcement	Legislation			Law Enforcement		Paradigm	Strafrecht / OM		Strafrecht / OM				Bestuursrecht / Boete		Bestuursrecht / Boete									
230	17	Law enforcement	Tools			Handhaving		Primary strategy	Per partner		Per partner				Per partner		Per partner									
231	17	Law enforcement	Tools			Handhaving		Strength	OM zaak aanbrengen		OM zaak aanbrengen				Boete opleggen		Boete opleggen									
232	17	Law enforcement	Tools			Handhaving		Bevoegdheid	BOA		BOA				MilieuBOA		MilieuBOA									
										0		0		0		0		0		0						
233	18	Create Safety knowledge	Evaluation Studies			Create Safety Knowledge		Evaluation Studies																		
234	18	Create Safety knowledge	Safety Information			Create Safety Knowledge		Safety Information	MHC ongevallen rapportage		MHC ongevallen rapportage															
235	18	Create Safety knowledge	Safety Research			Create Safety Knowledge		Safety Research	TU Delft, RIVM, TNO, COT		TU Delft, RIVM, TNO, COT				TU Delft, RIVM, TNO, COT		TU Delft, RIVM, TNO, COT									
236	18	Create Safety knowledge	Regulator Experience			Create Safety Knowledge		Regulator Experience	TU Delft		TU Delft				TU Delft		TU Delft									
										0		0		0		0		0		0						
237	19	Regulator organisation	Paradigm			Negotiations with industry on standards		Inspection SZW representation	SZW represented in LAT		SZW represented in LAT				-		-									
238	19	Regulator organisation	Paradigm			Negotiations with insp partners		Inspection Methods	Working groups with partners		Working groups, LAT				Working groups, LAT RB		Working groups, BRZO+									
239	19	Regulator organisation	Education & Training			Background info		Education & training organisation	Academie SZW		LAT BRZO Academie				Leer&Ontwikkelplein		Leer&Ontwikkelplein									
240	19	Regulator organisation	Manpower			Background info		Inspection organisation	Ministerie SZW, Arbeidsinspectie, 6 t		Ministerie SZW, Arbeidsinspectie, di				Ministerie SZW, InspectieSZW, directie MH		Ministerie SZW, Inspectie SZW, BRZO+,									
241	19	Regulator organisation	Manpower			General legislation		Environment	Wm		Wm				Wabo		Wabo									
242	19	Regulator organisation	Manpower			General legislation		Veiligheidsregios	Brw.wet		Brw.wet				Brw.wet		Brw.wet									
243	19	Regulator organisation	Manpower			Cooperation with Partners		Inspection partners	AI, WM, BRW		AI, WM, BRWregio				AI, Wabo/RUD, VeilRegio, Waterbeh, x,x,x.		AI, Wabo/RUD, VeilRegio, Waterbeh, x,									
244	19	Regulator organisation	Economy			Society		Economy	Up		down				down		up									
245	19	Regulator organisation	Public debate			Society		Public debate																		
246	19	Regulator organisation	Political situation			Society		Political situation	vvd-pvda		vvd-pvda				vvd		vvd									
247	19	Regulator organisation	Manpower			Inspection SZW organisation		Manpower	40 fte, 4 teams		40 fte, 4 teams				40 fte, 4 teams, shortage		50 fte 3 teams, shortage									
248	19	Regulator organisation	Manpower			Cooperation with partners		Inspection frequency	not considered critical		not considered critical				choices due to lack of manpower		choices due to lack of manpower									
249	19	Regulator organisation	Education & Training			Inspection SZW organisation		Budget	-		-				Drop		Increase									
250	19	Regulator organisation	Education & Training			Title		Education & Training programme	MHC basis opleiding		MHC opleiding				ATO		BOP		OMI							
										0		0		0		0		0		0						
		RESEARCHER																								
251	20	Researcher bias	Author involvement			Background info		Information sources	Beck 2003; Ervaring 1e auteur 2002						Beck 2014 Programma ATO 1402 najaar 20		Lindhout 2017 Offerte BOP-1 V5 dd 21 r									
252	20	Researcher bias	Change of perspective			Researcher Bias		1st Author involvement	just hired as MHC inspector (2002), p		involved in NIM development, starte				Updated contents of ATO programme, intro		Updated BOP programme changes, beca		Became tutot via B&D for							
										0		0		0		0		0		0						
		THEORETICAL FRAMEWORK																								
253	21	Professional standards	Auditing										NIM Inspection leader				ISO-19011, 2018									
254			Professional style						uniformity		BTO 2007															
255			inspection methods&techniques								NIM, GIR															
256	22	ETP Change process	Set goal						keep the same		keep the same															
257			determine content										less theory, more practice		revised contents											
258			execute														LOP team									
259			evaluate the outcome																							
260			implement change																							
261	23	Evaluation practice	Stakeholders								Hulst-Ouwerkerk 2008 report						Wingerde et al 2018									
262			Social relations																							
263			Setting																							
264			Normative framework																							
265	24	Educational objectives	Factual						MOOIS report, 2003				Hearts&Minds culture check development		KPI requirements (HSE 654)											
266			Conceptual																							
267			Procedural																							
268			Metacognitive																							
269	25	Quality of education	Trainee appreciation																							
270			Learning														Interactive style (UBR-HIS, 2019)									
271			Usage																							
272			Societal impact																							

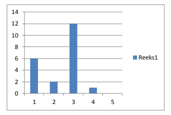

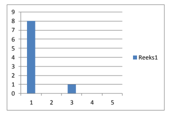

Supplement: Supplementary file 1 [file ijerph-17-01959-s001.zip › supplementary/Data file 1.pdf]
