# Supplementary material for: Improving Education and Training of Dutch Major Hazard Control Inspectors: A 15 Years Longitudinal Case Study
Source: Int J Environ Res Public Health. 2020 Mar 17;17(6):1959. doi: 10.3390/ijerph17061959 (PMC7143160; doi:10.3390/ijerph17061959)
Supplement: Supplementary file 1 [file ijerph-17-01959-s001.zip › supplementary/Data file 2 V1 - 25 Aspect descriptions text.pdf]

Data file 2 – 25 Aspect history descriptions

Files retrieved

For each of the time cross sections in this study, a detailed description of the ETP was obtained from internal MinSZW documents, via the 1st and 2nd author, compiling contents and course durations per element [See data file 1] (Beck, 2003; 2009; Beck, Lindhout, 2014; Lindhout, Werff, 2018; Nouwens Werff, 2018) and the spring 2019 tender information package (UBR-HIS, 2019). Per sub-section and per time section, the duration was expressed in course-hours of each unified ETP element in each of the ETP executions per time cross section was derived from calculations using both the duration data in the above mentioned documents and additional duration data derived from Beck (2013).
The execution performance was monitored and evaluated after programme execution, using questionnaires to be completed by the programme participants. This information was available for the 2003 (Beck, 2004), 2014 (Beck, 2014) and 2018 (Lindhout, 2018) executions.
The contents of the ETP were internally evaluated several times, in 2013 (Lindhout, 2013) and in 2017 (Lindhout, Werff, Dort, 2017).

ETP subject matter aspects 1-11

Aspect 1 Job requirements

An MHC inspector needs an engineering- or academic education (HBO/Acad), experience in industry or in an adjacent regulatory government organisation and preferably has process-, chemical- and safety knowledge. Candidates lacking safety education need to be prepared to go through formal safety education to technical college or master level. Their personality and behaviour is subject of an assessment to find out whether their background, drives and skills match with the profile requirements: ability to acquire new knowledge, broad experience, analytical skills, result oriented, societal awareness, team player, perseverance and meticulousness (SZW, 2002; SZW, 2003; ISZW, 2019). No significant differences were found between time cross sections.

Aspect 2 General legislation

The MHC inspector operates from a background consisting of three main parts:
1) occupational safety as laid down in the working conditions law (Arbowet, 2015),
2) law enforcement action based on either criminal laws (WED; Wet BOA) or administrative laws (AWB), and
3) special legislation (BRZO, 2015; RRZO, 2016), the Dutch implementation of the Seveso III directive (EC, 2012). In practice the MHC inspector is also involved in several issues governed by the environmental regulator (Wabo) and the safety regions (Brandweerwet).
From 2014 onwards the occupational safety part in the ETP increased significantly due to new or revised European directives, a.o. PED; ATEX 114, previously ATEX95; ATEX 153, previously ATEX137, and to a policy change at MinSZW regarding long term exposure to hazardous chemicals.

Aspect 3 Special legislation

The MHC inspector has competencies aimed at inspecting HRO chemical companies and enforcing the Dutch implementation (BRZO, 2015; RRZO, 2016) of the Seveso III directive (EC, 2012).
Several changes were observed in special legislation during the time period.
The first one was the implementation of the ARIE indication system which led to a steep increase in the number of companies to be inspected. Although similar to the BRZO (1999) legislation, there were considerable differences to take into account when inspecting this category of companies working with quantities of hazardous chemicals just under the BRZO

threshold. Just prior to the 2014 time section this work had been reallocated to the Labour Inspection and therefore disappeared from the MHC inspector ETP.
The second one was a gradual development of industrial standards on safety management systems (CPR20/RIB; NTA-8620; PGS-6) that aim at compliance with, and are kept up to date with, the successive changes in BRZO requirements. These standards were an explicit ETP subject in the 2003 time section only.
These BRZO releases are the third change, relevant to the MHC inspectors' task. In 2003 the BRZO (1999) version was in force. The safety management system requirements were laid out in seven main sections: 1-organisation and personnel, 2-risk assessment, 3-safe work, 4-change management, 5-emergency preparedness, 6-safety performance and 7-management review. These main sections 1 . . . 7 remained throughout the entire time period although they had been renamed and renumbered in the 2008 to b . . . h with a-safety policy as an added section. Keeping the added section under a, the seven sections were again renamed and renumbered to i . . . vii in the 2018 time cross section, due to BRZO (2015) getting into force after the new release of the European Seveso III directive in 2012 (EC, 2012).
The requirements of BRZO have been an explicit subject of the ETP in the 2003, 2008 and 2014 time cross sections in much the same way. In the 2018 time cross section, BRZO requirements have almost completely been integrated into safety management system ETP elements.
The fourth change in special legislation was the implementation of several laws in the environment control and emergency preparedness areas within the BRZO field. In practice the MHC inspector notices these changes in the cooperation with inspection partners but does not directly deal with it. These were therefore taken up as contextual factors. The fifth change came from new EC directives and Dutch standards being issued, concerning the practical implementation of the Seveso Directive and BRZO, affecting whether companies are implicated or not and what measures must be taken for specific hazardous substances.

Aspect 4 Cooperation

The MHC inspector is part of an inspection team when executing BRZO inspections at a company. If it comes to law enforcement all inspectors in the team act from the background of their own governmental institution, in the case of an MHC inspector that is the Ministry of Social Affairs and Employment. A typical BRZO inspection team in 2003 consisted of a representative of the local or regional Environmental government institution, a representative of the local or regional Fire Brigade and a MHC inspector.
In the 2003 time cross section the cooperation within an inspection team was based on verbal agreement between its member inspectors on the observations made during inspection. This could result in different views in the three reports issued under a cover letter on behalf of the environmental government institution, put in charge of the inspection. In successive steps this elementary form of cooperation was developed into a national platform with common appraisal criteria, a common planning system and a joint digital reporting platform, appearing in the 2014 and 2018 time cross sections. In the 2018 time section also a public summary report appears.
The MHC inspector is acquainted with the role of the other team members in a progressively larger portion of the ETP from 2003 to 2008 and 2014. In 2018 the time allocated to this aspect was approximately equal to the 2014 allocated duration for familiarizing with the roles of Environmental institutions, currently operating from regional RUD offices and Fire brigades, currently organised in Safety regions.
Two other parties were part of the ETP throughout the entire time period in this case study: the OVV and the HRO chemical companies. These were invited to express their own views on safety and their role in the BRZO field. This allows the MHC inspector to feel the independent investigative role the OVV has when it comes to the activities of the governmental

institutions in the BRZO field. Also the MHC inspector is confronted with the company paradigms around profitability and safety.

Aspect 5 How companies work

In the introduction phase of the ETP execution in each of the time sections, representative speakers of the chemical companies were invited to present their philosophies on company activities, especially the maintenance aspect, their approach of safety management and their view on the regulator role. From 2014 onwards this included 'safety performance indicators', in response to the Seveso III Directive (EC, 2012) introduction of new requirements on this point.

Aspect 6 Safety Management System

Safety Management can be done in different ways (Li & Guldenmund, 2018). The Seveso III Directive (EC, 2012) prescribes the main elements of a Safety Management System to be implemented by companies working with hazardous chemicals as indicated by the directive. Although there are minor changes in names and wording to be found when comparing BRZO (2015) contents with the BRZO (1999) original Safety Management System (SMS) description, its main structure remained largely the same over the time period. SMS requirements in ISO 31000:2009 were published between 2008 and 2014.

What MHC inspectors need to know about the SMS which BRZO companies must implement is mostly covered by this aspect, nr 6. Some of it is included in aspects nr 7 (part of SMS element ii) and nr 9 (part of SMS element vi).

The SMS elements I, ii, iii and vi get the most attention in the ETP of 2018. The SMS elements iv, v and vii get little attention by comparison in all four time cross sections. Only in 2018 all SMS elements are explicitly mentioned as a subject in the ETP. This is because the 2018 ETP focuses on how to inspect each SMS element in practice, whereas the 2003-2014 ETP's focused on why specific parts of the SMS elements are important and how they should be designed in theory.

A shift towards a more practical level in contents for mainly SMS element ii shows between the 2003-2008 and the 2014-2018 time sections, as virtually all reliability and probability theory was abandoned in the later versions of the ETP.

Several peaks were noticed in hours spent within the ETP: a slow but steady rise of SMS element i as safety culture emerges, a peak in iii in 2008 as awareness of poor maintenance related causality was brought to the attention of the BRZO inspection partners (MOOIS, 2003), and an increase in SMS vi, as safety performance indicators were included in the Seveso III directive (EC, 2012) for the first time.

In the 2018 ETP, theoretical knowledge transfer was partly replaced by learning from practice and a new focus on each SMS element, on inspection training, on risk assessment quality and on the law enforcement task.

Only in 2014 there is a significant dip in the total number of hours spent on the overall SMS content in the ETP.

Aspect 7 Process installations

The technical design of processes and various types of process installations have been a near to constant factor in the ETP throughout the entire time period. The European directives for working with explosive atmospheres (ATEX regulation) and high pressure (PED regulation) are addressed in the more recent ETP versions. The updated versions of the NPR standards are not explicitly mentioned in the ETP but are part of the 'ATEX-zone' subject. Bio-process technology and software architecture were introduced in the 2014 ETP but were discarded again before the 2018 time section.

Concerning the technical safety aspects of installations and its components however, the ETP shows a major shift from theory and calculus oriented towards practice oriented application of safety control systems. This transition takes place between 2014 and 2018.
The degradation mechanisms in process plants were present in the ETP on corrosion phenomena and material strength level throughout the time period. In addition to this explicit attention was given to ageing process plants in 2018 as an emerging factor in causality.

Aspect 8 Dangerous chemicals

MHC inspectors join in with existing occupational safety inspectors' training programmes throughout the entire time period. This underlines the common concern between the two domains 'occupational' and 'major hazards' when it comes to hazardous chemicals. The introduction of new European legislation on registration of chemicals and their dangers and about labelling requirements (GHS Regulation; REACH Regulation; CLP Regulation) since 2009 in transport and in process installations and storage facilities, resulted in new and adapted elements in the ETP from 2014 onwards.

Aspect 9 Learning from accidents

Accident history, and how to learn from it, was present as a subject in all time cross sections in this study. In a set of 15 to 20 accident cases being studied, a few more recent accident cases were replacing older cases in the ETP throughout the time period. An increased use of video material available from the internet was observed. This did not change the general objective and approach of this aspect in the ETP though. Continuous discussion about accident cases and learning how to deal with them and prevent any repeats was also part of aspect 11, coaching. Many "learning from accidents" meetings were held either within the confines of MinSZW, or together with Governmental BRZO stakeholders in the country.

Aspect 10 Learning from practice

New inexperienced MHC inspectors go on inspection with an experienced collegue belonging to the same team, at least for the first year. This practice exists since 2003 and continues until 2018. Incidental joint inspections with members of other teams have occurred in the entire time period too. Also incidental international exchange opportunities (EC Joint visits) were used throughout the case study period. In the 2018 time section several MHC inspectors had become involved in safety inspections held at the Dutch Caribbean BES islands together with local authorities.
Once every 4 to 5 years each MHC inspector participates in several 'repeat' training courses about process safety and on interpersonal skills. These have been present in all time cross sections.
Several changes in legislation and in standards have altered the contents of the ETP.
The subject of practical law enforcement skills became suddenly more important at the moment of getting in to force of the WAHHS legislation in 2014. Rather than bringing a company violation of the law to the juridical system, the MHC inspector could now get MinSZW to charge a substantial fine to a company without the intervention of the court. The ETP in 2014 and even more in 2018, contains explicit training courses, where unstructured learning by doing had been normal practice in the preceding cross sections.
Two minor new elements were added to the ETP in 2018: practice training at a newly built test site and – on voluntary basis – personal driving skills.

Aspect 11 Coaching

This aspect covers the remainder of ETP activities. The permanent part of that consists of regular national meetings to keep up to date with developments and to allow internal consultation of expertise needed for particular cases in inspection practice. In a similar manner, team meetings

ensure information exchange, training and feed-back on a personal level between colleagues. The other ETP aspect nr 11 part is a collection of ad-hoc events, training on available internal MinSZW resources and facilities, incidental events around introduction of new legislation, methods, accidents and cooperation, methods and systems updates.

Context aspects 12-19

Aspect 12 Safety inspection approach

The inspection approach changed due to the introduction of new decision making about the inspection subject, inspection frequencies and the evolution of the inspection method.

In 2003, inspection subjects were chosen by the inspection teams, with representatives from the three inspection partners, on basis of prior inspection results. By 2008 this was constrained for a part by an inspection team obligation to cover all SMS elements at a company within 5 years. By 2014 the inspection results per SMS element were monitored and shared via a joint online working platform.

Risk based inspections were done yearly at high BRZO level sites and with a 2 or 3 years interval at low BRZO level plants.

MHC inspectors were present as far as manpower was available. In the 2014 this had become a noticeable problem and in the 2018 time section manpower had grown to a major problem and was being addressed by hiring additional MHC inspectors.

The inspection method changed from AVRIM2 (2003) to NIVRIM (2008), to NIM (2014), NIM2 (2018) and in 2018 a new joint inspection method was being designed, and was provisionally named LBR.

Decision making and the paradigm, a.o. beliefs, values and assumptions at MinSZW with regard to the ETP did not change significantly until 2018 when a previous long period of rather small changes in content was ended and a step towards SMS practice and educational efficacy improvement was made.

Aspect 13 Reporting system

The development of a common online reporting platform between the inspection partners was a major change. In an evaluation of ‘customer satisfaction’ (BTO, 2007) reports were identified as slow and having poor readability. Starting from separate and not fully harmonized reports with a cover letter from each of the inspection partners in 2003, this development ended up at an integrated common online working environment for reporting, appraising, ranking, keeping records and planning of inspections and their results in 2014.

Aspect 14 Appraisal and ranking

Appraisal got more structured by introducing the “bad, weak, sufficient, good, excellent” NIM appraisal scale for appraisal of SMS elements during inspections.

For a while ranking of companies was done on this basis but this was gradually abandoned due to the sensitivity of the ranking for the SMS ‘sample’ taken during inspection and fluctuating data. A ranking list based on inspectors’ scores gathered in team meetings at each of the inspection partners had been introduced by 2014.

Aspect 15 Public information

The Seveso III directive (EC, 2012) requires transparency as a new requirement. Complying with this started in 2014 in the Netherlands with the first issue of a summary report about each inspection for the general public. Up to that moment yearly MHC accident review reports were issued by MinSZW in the first half of the time period in this case study. In the second half, specific annual major accident reports were no longer published. Instead, from 2013 onwards, extracted data from accident records in the Story-builder system (Bellamy et al, 2013), kept up to date by the RIVM, can be made available on request.

Aspect 16 Negotiations

Standards for use by companies when building their SMS evolved from CPR-20/RIB to NTA-8620 in 2008. Companies increasingly adopt ISO 9000, ISO 14000 and OHSAS 18000 standards.

Dutch technical standards changed from CPR series to PGS series. The single most important technical change originated from the Buncefield fire (Buncefield report) : the introduction of an independent overflow prevention valve, now required by PGS-29, first issued in 2008.

Aspect 17 Law enforcement.

The legislation was subject to several important changes throughout the time period of this study. The single most important one being the change over from criminal law based enforcement towards predominantly administrative law based enforcement. Where the former is subject to Ministry of Justice priorities, policy and the proceedings in the court system, the latter is fully under control of MinSZW.

From 2014 onwards the education and training effort for law enforcement via criminal law (Wet BOA) increased significantly due to a policy change at the Ministry of Justice.

In the same year MinSZW fines, assigned via administrative law, increased significantly (Wahhs), making legal action via criminal law the less effective path. The increased fines have strengthened the position of the MHC inspector.

Aspect 18 Create safety knowledge

Several former MinSZW employees with MHC experience laid down their knowledge in scientific journals and in articles in professional magazines, mostly in the 2014 to 2018 time period. Also several Moshe and HVK student inspectors contributed to MHC knowledge on Court case results, Maintenance and safety, Language issues, Unsafe alarm response, Uniformity, etc.

Availability of expertise to execute the ETP has not been a critical issue over the entire time period. A varying mix of internal MinSZW specialists, external company experts and specialists from scientific institutes was found for each time the ETP was executed. This mix was used to become more responsive to the BRZO field both on technical and managerial levels. A gradual changeover from external lecturers towards internal trainers occurs from 2014 onwards. In 2019 financial rules lead to external contracting.

Aspect 19 Regulator organisation

MHC inspectors were working in 4 regional teams in 2003. The team leaders took care of the coaching aspect and the general aspects of working in MinSZW. The team leaders reported to a manager in charge of the MHC inspection task at MinSZW. Not much has changed there over the time period although the organisation underwent several reorganisations. In 2003 the MHC task was part of the labour inspection section of MinSZW. By 2008 a separate MHC section with its own managing director had been established, including a separate strategic department. By 2014 this strategic department was merged with labour inspection strategic department. By 2018 the MHC task was no longer a separate section but had become part of the “hazardous chemicals programme” within (labour) “Inspection SZW”. The number of MHC teams was reduced to 3. In the 2018 time section a significant manpower increase had commenced after several years of problems with fulfilling the legal obligations to inspect BRZO companies sufficiently often. Many retirements lead to an influx of even more often young new inspectors.

Aspect 20 Researcher bias

The changing author roles over the time period are described in the method section.

The first author is familiar with the details of the ETP and its evolution from 2002 onwards from a position within the Dutch Labour Inspectorate, part of MinSZW.

The first author went from apprentice in 2003 to skilled inspector in 2008. By 2013 he became involved in updating the MHC-ETP with new technological developments in the field. These were included in the 2014 MHC-ETP. From then on, and also after retiring in 2016 as MHC inspector, he remained involved in the MHC-ETP development and joined the 2nd author in a full educational innovation. He currently is the tutor for the safety management system and risk assessment related content parts.
The second author is labour inspector at MinSZW since 2006. He is currently involved both in labour inspection and in the interdepartmental *Leer & Ontwikkel Plein* (LOP) team at the Ministry of Social Affairs and Employment. He currently is actively involved in ETP development, planning and improvement of the educational process.
This study was conducted under the supervision of the third author, professor at the Delft Technical University, Technology, Policy and Management (TPM) faculty, Safety & Security Science group. Noticing a lack of insight in the relationship between the regulator inspectors ETP and the changing society, he initiated this study.

Theoretical framework aspects 21-25

<u>Aspect 21 Professional standards</u>

The elements: Auditing, Professional style, inspection methods & techniques
As a consequence of the successive introduction of new inspection methods (see aspect 12) and the development of a joint inspection partners platform (see aspect 4) the ETP incorporated several new training courses affecting the MHC inspectors' professional style. Starting from the mind-set to do 'uniform' inspections using common appraisal guidelines in 2003 not much had changed in 2008. A poll among the HRO Chemical companies identified uniformity of MHC inspectors' style across the country as a problem to be solved (BTO, 2007). After the introduction of NIM, starting in 2007, more changes started to follow.
It took until 2008 for a unified appraisal system to become part of the BRZO+/GIR joint inspection partners common ICT and knowledge sharing platform.
By 2014 a NIM inspection leader training had been introduced.
Cooperation between inspection partners was intensified resulting in a new inspection style, alternating between System- and Measures oriented inspections and application of a 5 years cycle to cover a company SMS in its entirety. The focus was kept at risk based inspection.
Stronger law enforcement tools (WAHHS) were introduced (see aspect 23) leading to more emphasis on administrative law enforcement in the ETP.
A long ongoing debate on the viability of a certification SMS for HRO chemical companies was ended in 2014 by agreement on the notion that regulator "inspection must continue".
In 2018 the ISO-19011, 2018 Guidelines for auditing management systems were published.

Aspect 22 ETP Change process

The elements: Set goal, determine content, execute, evaluate the outcome, implement Change.
The ETP was kept the same as much as practical over the first two time cross-sections 2003 and 2008. Not many participants were available for a proper execution of the education and training programme and consequently not much effort was invested in improvement of the ETP. This changed significantly from 2013, triggered by increasing influx in the MHC department of new inspectors. Establishing a new approach with a less theoretical and more practice oriented ETP became an important guideline. The contribution of companies to the ETP execution was reduced and internal MinSZW experience was more used. Several new subjects were introduced and the emphasis on several existing subjects was reduced. By 2017 a new interdepartmental education management team (LOP team) started to raise the education standard level.

Aspect 23 Evaluation practice

The elements: Stakeholders, Social relations, Setting, Normative framework
Two distinct evaluation subjects were addressed: 1) in 2008 Hulst-Ouwerkerk (2008) investigated the effectiveness of criminal law enforcement in the BRZO field and concluded this was poor, and 2) after the introduction of administrative law enforcement the burden on the inspectors increased (Wingerde et al., 2018).
Several times since 2007 an external customer satisfaction investigation (BTO, 2007) was done, triggered by the KMS, the ISO-9000 based MHC department quality system.

Aspect 24 Educational objectives

The elements: Factual, Conceptual, Procedural, Metacognitive
Between the 2003 and 2008 time sections the ETP stayed much the same. By 2008 the notion that abnormal situations in process plants, such as maintenance stops, are more dangerous (MOOIS, 2003) had become a priority. In the 2014 ETP and more so in the 2018 ETP both a reduction of emphasis on several theoretical subjects and an increased emphasis on inspection and practice were implemented.
Safety culture as a concept sparked a separate training course from 2014 onwards. A method to assess company safety culture based on the Hearts&Minds method is under development since 2011 at the BRZO Academy. (Since then it is evaluated with a.o. TNO in 2015 , tested in practice in 2016 and finalized into "Inschattingsinstrument 2.0" by 2017. In 2018 it was planned to be introduced into the LBR inspection method which became delayed until further notice.)
After the issue of the Seveso III directive (EC, 2012) the introduction of key performance indicators in a company SMS became mandatory. HSE 254 KPI guidance is available here.
In 2014 the subject of language related safety issues was introduced into the ETP.

Aspect 25 Quality of education

The elements: Trainees appreciation, Learning, Usage in practice, Societal impact
The explicit use of the "audit trail" approach was introduced in the NIM part of the ETP.
In 2018 the safety part in the ETP was completely revised and restructured around the safety management system elements. The emphasis was increased on risk management.
The technical part of the ETP was kept largely the same with exception of an updated selection of major accident cases, the introduction of software- and ict related subjects and the introduction of a test site training.
Van der Hilst (2019, p287) describes the teachers' professionalism within a collective. The teachers in the MHC-ETP were mainly external specialists from companies upto 2014. Their efforts were weakly coordinated via the coordination at MinSZW. After 2014 the teachers were increasingly working from a collective and their mutual coordination was ensured in 2019 by sourcing the ETP almost completely from a single supplier (D&F).
By 2018 an interactive teaching style was adopted and an e-learning module for language related safety measures was developed. The inspectors' toolbox - and how to use it - became a separate subject (UBR-HIS, 2019).
Usage in practice and Societal impact are researched via a survey in this study among MHC inspectors having completed the MHC ETP in the time period.
The Societal impact was investigated via several stakeholder satisfaction surveys. Also external evaluation provides insight into societal impact. (See article text).

References

Arbowet, 2015 [see reference listing in manuscript]

ARIE regeling https://brzoplus.nl/publish/pages/140970/wijziging_7_februari_2004arieregeling.pdf [Accessed December 9, 2019]

ATEX 95,137 regulation [see reference listing in manuscript]

ATEX 114,153 regulation [see reference listing in manuscript]

AVRIM2 (2003) SZW-MHC Interne beschrijving Avrim 2 inspectie methodiek

AWB (2020) [see reference listing in manuscript]

Beck, 2003 Beck H (2003) Programma MHC- Basis opleiding 2003

Beck, 2004 Beck H (2004) Samenvatting cursisten evaluatie MHC 03-1

Beck, 2009 Beck H (2008) Programma 2004-2005 MHC 04-01

Beck, 2013 Beck H (2013) Codering namen dagen opleidings profiel MHC 2 sept

Beck, 2014 Totaaloverzicht evaluatie ATO 1401

Beck, Lindhout, 2014 Beck H, Lindhout P (2014) Programma ATO 1402 najaar 2014

(Bellamy et al, 2013) Linda J. Bellamy, Martijn Mud, Henk Jan Manuel, Joy I.H. Oh (2013) Analysis of underlying causes of investigated loss of containment incidents in Dutch Seveso plants using the Storybuilder method. Journal of Loss Prevention in the Process Industries (2013) 1-21.

Brandweerwet 1985 [now obsolete per 1-10-2010], is in 2010 vervangen door Wvr (2010). Wet veiligheidsregio's, Geldend van 01-01-2020 t/m heden https://wetten.overheid.nl/BWBR0027466/2020-01-01

BRZO (1999) Besluit Risico's Zware Ongevallen 1999 [now obsolete] is vervangen door:
BRZO, 2015 [see reference listing in manuscript]

BRZO+ [see reference listing in manuscript]

BTO, 2007 [see reference listing in manuscript]

Buncefield report COMAH (2011) Buncefield: Why did it happen? The underlying causes of the explosion and fire at the Buncefield oil storage depot, Hemel Hempstead, Hertfordshire on 11 December 2005. COMAH, UK, 2011. https://www.hse.gov.uk/comah/buncefield/buncefield-report.pdf

CLP Regulation [see reference listing in manuscript]

CPR series : [now obsolete]
CPR20/RIB is vervangen door PGS reeks, PGS-15, PGS-6

(EC, 2012) [see reference listing in manuscript]

EC Joint visits Mutual visits between Major Hazard Control institutions in EC Countries via JRC, Ispra Italy. https://ec.europa.eu/jrc/en/about/visitors-centre

GHS Regulation: [see reference listing in manuscript]

GIR Gemeenschappelijke Inspectie Ruimte. Digitaal online platform voor inspectiewerkzaamheden binnen BRZO+ [Voorheen LAT]

Hearts&Minds Safety Culture appraisal tool (BRZO academie, 2011) oorspronkelijk afkomstig van Shell.

HSE 254 (2006) Developing process safety indicators A step-by-step guide for chemical and major hazard Industries ISBN 978 0 7176 6180 0. https://www.hse.gov.uk/pUbns/priced/hsg254.pdf

Hulst-Ouwerkerk (2008) [see reference listing in manuscript]

ISO 19011, 2018 [see reference listing in manuscript]

ISO 31000:2009 [see reference listing in manuscript]

ISO 9000 ISO 9000:2015 Quality management systems – Fundamentals and vocabulary https://www.iso.org/standard/45481.html

ISO 14000 [see reference listing in manuscript]

(ISZW, 2019) Inspectie SZW (2019) Wervingstekst in advertentie MHC inspecteurs

(KMS) KMS SZW-MHC Kwaliteits Management Systeem, beschrijving MHC proces en procedures.

LBR Landelijke Benadering Risicobedrijven, inspectie methodiek doorontwikkeling vanuit NIM II. https://brzoplus.nl/actueel/nieuwsberichten/nieuwsberichten/nieuwsberichten-2017/november-2017/

(Li & Guldenmund, 2018) [see reference listing in manuscript]

(Lindhout, 2013) Lindhout P (2013) Voorstel voor aanpassing MHC opleiding

(Lindhout, Werff, Dort, 2017) Lindhout P, Werff K vd, Dort Rv, (2017) Discussie notities MHC Basis opleiding Procesveiligheid

(Lindhout, 2018) Lindhout P (2018) Resultaat Registratie BOP-1 cursusdeelnemers

(Lindhout, Werff, 2018) Lindhout P, Werff K vd (2018) Ontwerp MHC opleiding met BOP-2

(MOOIS, 2003) MOOIS Onderzoeksrapport Onderhouds- en inspectiesystemen bij BRZO-bedrijven, A. De Bokx-Verlinde, A. Dees, W. van Gils-van Brink, Arbeidsinspectie directie MHC, Oktober 2003.

NIM Nieuwe Inspectie Methodiek
NIM (2014) [now obsolete] is vervangen door:
NIM2 (2018)
https://brzoplus.nl/werkwijzer/werkwijzer-brzo-ii-aangepast-brzo15/inspectie-aangepast-brzo15/nim-detail/

NIVRIM (2008) [now obsolete] is vervangen door: NIM

Nouwens Werff (2018) Nouwens, B., v.d. Werff, K. (2018) Ontwikkeling OMI opleiding. Ministerie SZW.

NPR standards [see reference listing in manuscript]

NTA-8620:2016 nl [see reference listing in manuscript]

OHSAS 18000/18001 [see reference listing in manuscript]

PED regulation [see reference listing in manuscript]

PGS series Standards: http://publicatiereeksgevaarlijkestoffen.nl/ a.o.:
PGS-6 http://publicatiereeksgevaarlijkestoffen.nl/publicaties/PGS6.html
PGS-29 http://publicatiereeksgevaarlijkestoffen.nl/publicaties/PGS29.html

REACH Regulation: [see reference listing in manuscript]

RRZO, 2016 [see reference listing in manuscript]

(SZW, 2002) SZW (2002) Specialisten major Hazard control v/m Regio Oost. Arbeidsinspectie Arnhem. Werkenbijhetrijk.nl, sector_rijk/vacaturebank [Accessed 04-02-2002]

(SZW, 2003) SZW (2003) Inspecteur Major Hazard Control (v/m). SZW Vacatures 13e jaargang. 12-02-2003.

(UBR-HIS 2019) UBR-HIS (2019) Beschrijvend Document - Doorontwikkelen opleiding MHC. Versie 1.0, definitief, Kenmerk 201800273-102, 30-01-2019. Annex C - Nadere beschrijving modules van opleiding MHC inspecteur. Available from TenderNed.nl [Accessed Feb 11, 2019]

Van der Hilst (2019) [see reference listing in manuscript]

Wabo (2018) [see reference listing in manuscript]

Wahhs [now obsolete] is vervangen door directe werking van Arbeidsomstandigheden wet via:Beleidsregel handhaving- en sanctioneringkader Besluit risico's zware ongevallen 2015 arbeidsomstandighedenwetgeving. https://zoek.officielebekendmakingen.nl/stcrt-2015-22450.html

WED (2020) [see reference listing in manuscript]

Wet BOA (2019) [see reference listing in manuscript]

(Wingerde et al., 2018) [see reference listing in manuscript]
