# Supplementary material for: Improving Education and Training of Dutch Major Hazard Control Inspectors: A 15 Years Longitudinal Case Study
Source: Int J Environ Res Public Health. 2020 Mar 17;17(6):1959. doi: 10.3390/ijerph17061959 (PMC7143160; doi:10.3390/ijerph17061959)
Supplement: Supplementary file 1 [file ijerph-17-01959-s001.zip › supplementary/Data file 3 V1 - Survey MHC ETP study.pdf]

[1. Automatisch aangemaakt analyse rapport] [IN DUTCH]

Survey MHC ETP study		aantal	procent
Aantal verzonden formulieren		63	
Aantal terug ontvangen formulieren		32	
Waarvan ongeldig		1	
totale enquete	respons	31	49,2
totale enquete	non-respons	32	50,8

ALGEMENE VRAGEN

1-In welk jaar kwam u in dienst bij de MHC groep binnen de Inspectie SZW ?

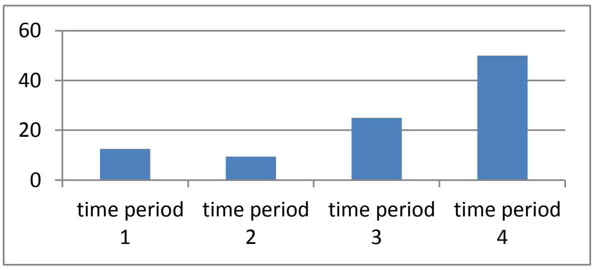

	MHC jaar in	aantal	procent
voor 1999	< 1999	1	3
1999-2003	time period 1	4	12
2004-2007	time period 2	3	9
2008-2014	time period 3	8	25
2015-2020	time period 4	16	50
	all time periods	32	100

vraag non-respons -3,2258065

2-In welke functie was dit ?

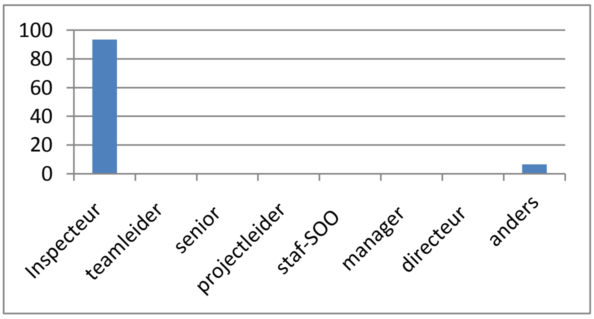

Functie in	aantal	procent
Inspecteur	29	94
teamleider	0	0
senior	0	0
projectleider	0	0
staf-SOO	0	0
manager	0	0
directeur	0	0
anders	2	6
	31	100

[Anders: opmerkingen niet relevant]

vraag non-respons 0

3-Welke functie vervult u op dit moment ?

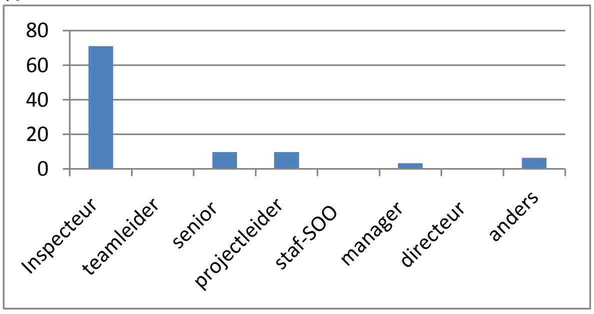

Functie nu	aantal	procent
Inspecteur	22	71
teamleider	0	0
senior	3	10
projectleider	3	10
staf-SOO	0	0
manager	1	3
directeur	0	0
anders	2	6
	31	100

[Anders: opmerkingen niet relevant]

vraag non-respons 0

4-Op welke manier(en) heeft u met de ontwikkeling van de MHC opleiding te maken gehad ?

%

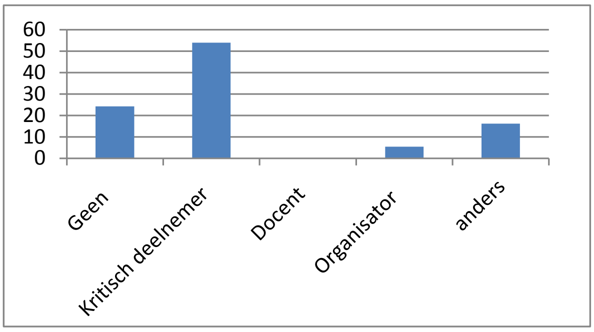

Rol bij ETP	aantal	procent
Geen	9	24
Kritisch deelnemer	20	54
Docent	0	0
Organisator	2	5
anders	6	16
	37	100

[Anders: opmerkingen niet relevant]

et
el)
--

vraag non-respons -19

Vragen aan management (a)

5-Wat ziet u als de belangrijkste doelen van de MHC opleiding ?

Doel idee	aantal	procent
	10	

Een gedegen basis te leggen in termen van theoretische kennis over procesveiligheid in combinatie met het creëren van een begrip van algemeen geaccepteerde concepten binnen het domein van procesveiligheid.---------Doel is om een deskundige inspecteur het inspectiewerk uit te laten voeren.
De MHC opleiding dient ervoor te zorgen dat de inspecteur zijn werk goed kan uitvoeren, weet wanneer hij/zij niet voldoende weet van een onderwerp, situaties goed kan beoordelen etc.
---- ----vakkundigheid en vaardigheid vergroten--Zorgen voor voldoende basiskennis bij nieuwe inspecteurs, bijhouden van relevante kennis bij zittende inspecteurs en doorontwikkelen van het vak van inspecteur--Het voorzien in benodigde basiskennis en m.b.t. bepaalde (tijdsgebonden) onderwerpen, voorzien in gedetailleerde kennis- -Procesveiligheid, post HVK. Goede counterpart bij inspecties.---Dat de MHC inspecteurs de kennis en kunde hebben om hun taken m.b.t. het inspecteren van BRZO bedrijven, op een professionele uitvoeren volgens de vastgestelde normen binnen het handhavingsbeleid.-Zorgen dat inspecteurs allemaal hetzelfde minimale kennisniveau hebben en wanneer je weet dat je hulp moet inschakelen van mede inspecteurs--------------------------------

Analyse vraag 5: Deskundigheid in process safety en vaardigheden als inspecteur.

vraag non-respons 68

6-Op welk opleidingsniveau richt de MHC opleiding zich volgens u ?

%

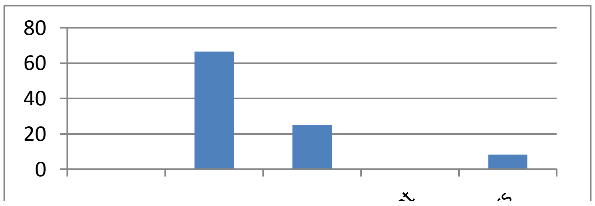

Richtniveau	aantal	procent
MBO	0	0
HBO	8	67
academisch	3	25
weet niet	0	0
anders	1	8

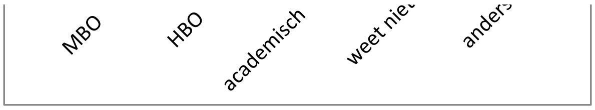

12	100

[Anders: opmerkingen niet relevant]

vraag non-respons 61

7-Op welk kennis type richt de MHC opleiding zich volgens u ?

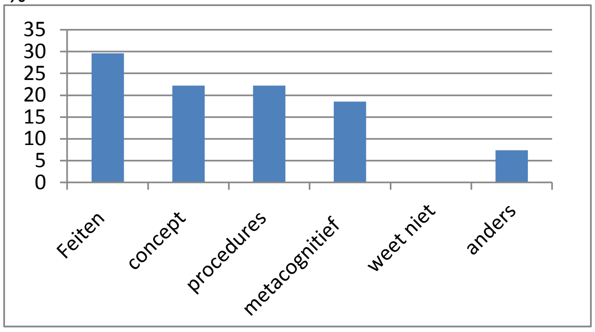

Type kennis	aantal scores	procent
Feiten	8	30
concept	6	22
procedures	6	22
metacognitief	5	19
weet niet	0	0
anders	2	7
	27	100

[Anders: opmerkingen niet relevant]

aantal respondenten: 8

vraag non-respons 74

8-Hoe zou u vast stellen dat deelnemers dit type ook bereikt hebben ?

Hoevaststel	aantal	procent
	9	

Door middel van het toepassen van opgedane kennis in de inspectiepraktijk zou moeten blijken dat cursisten de theorie beheersen en concepten begrijpen.---------afhankelijk van de opleiding (bijvoorbeeld NIM kent een examen). Bij verschillende opleidingen kiezen voor de professionaliteit van de medewerker, als deze inschat dat hij de lesstof niet in voldoende mate beheerst dan moet hij dat aangeven. Dus als organisatie zitten we dan meer op de aanwezigheid van een inspecteur bij een opleiding dan op het vaststellen of hij de lesstof voldoende beheerst--------in de praktijk meelopen, je ziet kennisniveau ook terug in inspectierapportage, intervisie--Door een soort afsluitende “toets”. Nu is deelname voldoende zonder dat nagegaan wordt of de stof ook is begrepen--Registratie deelname- - Interactief. Memotrainer. Meer dan alleen ‘heeft training gevolgd’---Eigenlijk wat nu niet zo zeer gebeurd en evalueren van inspectiebezoeken in samenwerking met het bezochte bedrijf.-Kennis kan op verschillende manieren opdrachten, examen, etc.-------------------------------

Analyse vraag 8:
Blijkt uit de praktijk o.a. via rapportages en intervisie, examen of toets. Alleen aanwezigheid registreren is niet genoeg.

vraag non-respons 71

9-Om welke redenen werd en wordt er volgens u aan de MHC opleiding gesleuteld ?

Sleutelreden	aantal	procent
	9	

Veranderende wetgeving en vernieuwende inzichten op het gebied van procesveiligheid. ---------Om de opleidingen actueel te houden. Als er wetgeving veranderd, als er ontwikkelingen zijn in het vakgebeid etc. Ook als senior inspecteurs aangeven dat er behoefte is aan een verdieping op een bepaald onderwerp. Opleidingen zijn ook ontwikkeld als er bepaalde projecten werden gestart (denk bijvoorbeeld aan de module taal)--------enerzijds mbt inhoud obv behoefte, voortschrijdend inzicht en benodigde vaardigheden, anderzijds om didactische redenen; welke vorm/stijl past het beste vwb kennisoverdracht
--Vanwege nieuwe inzichten, nieuwe taakvelden, diepgang van de inspecties--Het Brzo werkveld is aan verandering onderhevig, dus continue evaluatie en bijstelling is noodzakelijk. Een pdca cyclus voor opleidingen dus.- - Verbetering. Maar er is wel erg veel aan gewijzigd telkens. Als is het alleen al de naam. Hierdoor zijn nieuwe inspecteurs die de gehele opleiding moeten volgen telkens net weer anders getraind. En er vindt geen her- en bijscholing plaats van zittende inspecteurs die de nieuwe onderdelen van de MHC opleiding (waarvan men kennelijk vond dat dit belangrijk is in de opleiding en training) nog niet hebben ontvangen. Dit is raar en levert verschillend getrainde inspecteurs op.---Omdat grotendeels de modules gegeven werden gebaseerd op het passieve leren en niet op het actieve leren m.a.w. andere leervormen en niet alleen maar PowerPoint presentaties kijken-Wijziging ten aanzien van minimale kennisniveau, andere inzichten over wijze van opleiden, kosten van de opleiding, etc --------------------------------

Analyse vraag 9: Bijblijven met veranderingen in wetgeving , vakgebied en inzichten. Her- en bijscholing ontbreekt en zorgt voor opleidingsverschil tussen inspecteurs

vraag non-respons 71

Vragen aan inspecteurs (b)

10-In welk jaar (of jaren) volgde u de MHC opleiding ?

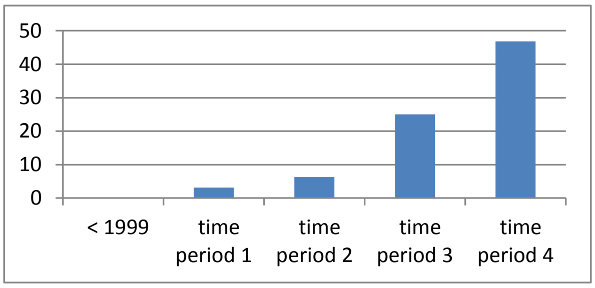

	ETP startjaar<br>ETP eindjaar	aantal	procent
voor 1999	< 1999	0	0
1999-2003	time period 1	1	3
2004-2007	time period 2	2	6
2008-2014	time period 3	8	25
2015-2020	time period 4	15	47
	all time periods	26	81

Gemiddelde ETPlooptijd 1,1 jaar

vraag non-respons 16

11-Wat vond u van de aangedragen kennis in uw MHC opleiding ?

Kennis mening	aantal	procent
	25	

Van het juiste niveau voor een MHC inspecteur.-Prima evenals de interactie met docenten en andere inspecteurs. Die combinatie maakte het heel waardevol al was er soms sprake van een overload aan cursusdagen in een te korte periode. Een betere spreiding had bijgedragen in de verhoging van het kennisniveau -Destijds vooral de basiskennis over wetgeving en BRZO-Goed, al was het soms zoeken hoe dit specifiek toegepast kan worden. Het is soms ook lastig om in 3 uurtjes tijd een onderwerp te behandelen en daar info en inspiratie in op te doen om het te gebruiken bij een inspectie. Wel fijn dat er een lesgegeven werd door jou (Paul) omdat jij ook uit het werkveld kwam. Daarentegen was de opleiding gegeven door D&F ook zeer goed, ondanks dat Roger geen inspectie ervaring had, had hij wel heel veel proces (safety) kennis. -nuttig en goed -Veel en relevante informatie en kennis -Was soms op een veel te gedetailleerd, abstract, theoretisch en academisch niveau. Bijvoorbeeld over reactiekinetiek.-Inhoudelijke vakken goed.

Juridische achtergronden en hoe op juridische juiste wijze gehandeld moet worden in de praktijk en om juridisch 'houdbare' rapporten en brieven te schrijven matig.

Ten aanzien van de inhoudelijke vakken Atex en drukapparatuur merk ik op dat deze met te weinig diepgang zijn behandeld. Voor Atex geldt dat er binnen MHC m.i. nog steeds een kennisachterstand is over het gedrag van (brandbare) dampen in de praktijk en voor ATEX en drukapparatuur/PED er nog een kennisachterstand is over de structuur van de productwetgeving.

-Over het algemeen nuttig en voldoende diepgang, behalve het onderwerp 'ontwerp procesinstallaties': de aangereikte kennis bij deze module was te uitgebreid om in de beschikbare tijd te behandelen (mensen haken af), ook was de aangereikte kennis te diepgaand/specialistisch (details zullen in de inspectiepraktijk nauwelijks gebruikt worden). --Goed-De aangedragen kennis was vooral gericht op technisch gebied. Op het gebied van toezicht & handhaving is er geen opleiding gegeven.-Goed-Goed en uitgebreid-Gezien mijn achtergrond deels nieuw en overgrote deel een refreshment-Breed opleidingsprogramma variërend van techniek tot wetgeving. De inhoud daarvan was erg goed maar de hoeveelheid en intensiteit van de opleiding maakte dat de kennis deels inbedde. Pas later tijdens de inspecties heb je de kennis daadwerkelijk nodig en is de kennis niet paraat. Wel merk ik dat na opnieuw te verdiepen kennis weer op komt borrelen en dat uiteindelijk toch van leerrendement sprake is. Het zou mooi zijn dat je thematisch studeert en dit projectmatig direct, al dan niet met stagebegeleider/mentor, in de inspectiepraktijk kunt toepassen.-Voor zover ik nu kan beoordelen zeer uitgebreid--Over het algemeen voldoende--te veel detail en t e veel cursusdagen in een korte periode, waardoor ik onvoldoende de stof kon verwerken/opnemen-Nuttig, maar soms ook teveel en op het verkeerde moment- Doordat ik bij de omgevingsdienst al de nodige ervaring had met Brzo waren niet alle onderwerpen even interessant om te volgen . Het meest leerzaam waren voor mij opleidingen waar ik voorheen niet mee in aanraking ben gekomen (vnl. ATEX en PED). Het bespreken van de VBS elementen blijft altijd interessant. Het is nl niet zwart/wit. Discussie levert ook vernieuwde inzichten. - Goed. Sloot goed aan.-De aangedragen kennis door interne en externe docenten was enorm. Daar heb ik veel aan gehad. Alle docenten kwamen uit het werkveld waardoor veel inhoudelijke kennis aanwezig is met veelal interessante praktijkvoorbeelden.-Nuttig en waardevol.---De kennis die werd aangedragen was over het algemeen van een goed niveau.-Prima, werd gegeven door ervaren collega's-Verfrissend. Ik was hiervoor reeds werkzaam binnen het werkveld als Brzo inspecteur WABO maar heb de opleiding enerzijds als verfrissend (vooral een andere kijk op het VBS) anderzijds als meer verdiepend (procesveiligheid, ATEX) ervaren.-----------------------------

Analyse vraag 11: Ruim de helft van de respondenten noemt de MHC opleiding goed, nuttig en uitgebreid. Samen optrekken is prettig.

De andere helft geeft enkele kritiekpunten aan:

1-Het opnemen van al de kennis wordt bemoeilijkt door o.a. de veelheid, hoog tempo, abstractieniveau, details.

2-Enkele noemen aanreiken van kennis op een verkeerd moment zodat kennis wegzakt voor die toegepast kan worden

3-Op gebied van HH, AREX en PED noemen sommigen dat ze meer kennis aangereikt hadden willen krijgen.

vraag	non-respons	19

12-Wat vond u van de opzet en organisatie van uw MHC opleiding ?

Opzet mening	aantal	procent
	26	

De opleiding is goed georganiseerd. Omdat de inspecteurs deze aan het begin van hun carrière als MHC inspecteur volgen in een relatief kort tijdsbestek, is er weinig ruimte om gedurende de opleiding het geleerde in praktijk te brengen en hierop gedurende de opleiding te reflecteren.-Zie boven. Ik vond het ook heel waardevol om gedurende een langere periode met 'jaargenoten' op te trekken zodat een begin van intern netwerk kon worden opgebouwd. Dat is nu nog steeds waardevol en zinvol -De basis was feitenkennis in de PVI-opleiding werd er veel meer ervaringen uitgewisseld.-We hebben een rommelige start gehad, zoals eerder gezegd in 2017 begonnen maar toen was er geen MHC opleiding. Daardoor een jaar voornamelijk praktijk opleiding gehad. Ook in feedback wel aangegeven dat sommige dagen erg lang waren en het daardoor moeilijk is om aandacht te houden bij de les.-prima-Goed, enige blokken waren erg druk door meerdere gelijktijdige opleidingen, bv OMI en HVK opleiding-Wel goed-Goed. -prima--Wat laat gestart in relatie tot indienstreding-De opzet en organisatie waren in zijn algemeen goed. Ik ben echter wel van mening dat de opleiding bij sommige modules langer had mogen duren. Door de te korte opleiding zijn een aantal onderwerpen niet of slechts zijdelings aan de orde geweest.-Te snel, weinig mogelijkheden om het geleerde in de praktijk te gebruiken-Goed -De organisatie was goed. Echter door een te krappe tijdsplanning was er onvoldoende tijd en gelegenheid om de opgedane kennis te laten bezinken-Zoals bij vraag 11 beantwoord goed maar veel. Sommige opleidingsdagen waren minder effectief doordat slechts een deel van de dag werd benut voor opleiding (een vroegertje)-Het is een langdurig opleidingstraject, waar ook wel overlap in zit-- Als nieuwe inspecteur kreeg ik de BOP-opleiding. Ik heb deze ervaren als leerzaam en intensief. Intensief omdat we ernaast veel wilden meelopen met zittende MHC-inspecteurs, was het zo nu en dan onmogelijk om samen opdrachten uit te voeren. Het bespreken van de opdrachten door fysiek bij elkaar komen vind ik waardevoller dan de taken verdelen en ieder zijn eigen deel individueel uitvoeren. Leerzaam om ik voorheen geen inspectie ervaring had, maar wel ervaring bij Brzo-bedrijven.

De MHC-opleiding voor zittende inspecteurs heb ik ook gevolgd voor drukapparatuur n.a.v. het ageing-project en themagerichte inspecties. De drukapparatuuropleiding heb ik eerder gevolgd dan dit deel van de BOP-opleiding. Ik kon het goed volgen, maar hoorde ook van andere nieuwe inspecteurs dat ze hier meer moeite mee hadden. Die voor de themagerichte inspecties was goed te volgen. De opzet en organisatie van beide opleidingen vind ik prima. Zou misschien wat meer interactief gemogen.

--zie 11-Wel goed en behoorlijk professioneel t.o.v. collega organisaties (inspectiepartners).- De opzet en organisatie was prima. De combinatie met veel andere verplichtingen (uitvoeren inspecties, teamoverleggen, andere cursussen zoals HVK) en ook reistijd (in mijn geval per keer in totaal 4,5) maakt dat het wel bepaalde druk legt op het privé leven- Voor zover ik nog weet: Geen opmerkingen. Ik hoor wel dat de opleiding nu veel uitgebreider is. Met bijvoorbeeld ook de proeffabriek. Dit is voor zittende inspecteurs niet gegeven.-De opzet was goed. De opbouw van de modules was logisch. Wat mij betreft had er meer tijd mogen zitten tussen de lessen, zodat er meer ruimte is voor meelopen met ervaren collega's. Nu zaten de lessen dicht op elkaar waardoor het geleerde niet in de praktijk gebracht kon worden.

De organisatie zat goed in elkaar. Karel heeft ervoor gezorgd dat er altijd een ruimte en een lunch beschikbaar was. Er was voldoende leer- en oefenmateriaal.

-Veel informatie in korte tijd (naast alle andere werkzaamheden)---Over het algemeen goed-Goed, dagen waren in overleg gepland en er zat voldoende tijd tussen de blokken-Goed, vanwege het stimulerende aspect aan de groep (presentaties geven over onderwerpen) maar dit kan verbeterd worden door meer praktijkgericht te werken.-----------------------------

Analyse vraag 12:

Ruim de helft van de respondenten noemt de opzet en organisatie goed . Het niveau is goed en het samen optrekken en ervaringen uitwisselen wordt als nuttig ervaren. Ook hier een aantal kritiekpunten:

1-Het opnemen van al de kennis wordt bemoeilijkt door o.a. de veelheid en hoog tempo, lange dagen of halve dagen, meer opleidingen naast elkaar.

2-Enkele respondenten noemen de geringe mogelijkheden om de kennis in praktijk te brengen en willen ruimte voor meelopen in praktijk.

3-Enkelen zien overlaps en noemen dat het themagerichte deel meer interactief zou kunnen.

vraag	non-respons	16

13-Heeft u opmerkingen bij de keuze en stijl van de docenten ?

Docentstijl	aantal	procent
	26	

Een goede mix van docenten, maakt een mooi evenwicht in de opleiding. Ervaren inspecteurs met veel praktijkervaring (Peter & Anton) in combinatie met een sterk technisch onderlegd docent (Roger, D&F) en een meer beschouwend docent (Paul).-Ach ja, de één was natuurlijk beter dan de ander, zoals overal. Een enthousiaste docent inspireert uiteraard meer dan een wat mattere -In de PVI was er ruimte voor discussie en heb dit als zeer prettig maar vooral leerzaam ervaren-Nee was prima, goede afwisseling theorie en praktijk (net als in het echte werkveld).-nee-interactieve opdrachten zijn leerzaam, met groepje vragen uitwerken-Soms veel te gedetailleerd. Anderen (vooral uit het bedrijfsleven en adviesbureaus) lieten heel graag merken dat zij het geweldig vonden dat zij inspecteurs van de overheid mochten opleiden en lieten dat desgewenst ook graag merken.-Goed, mede omdat er goede mix van interne- en externe docenten was. -Geen opmerkingen, positief in de inzet van docenten met veel praktijkervaring met inspecties--Nee-Ik vond dat sommige docenten didactische vaardigheden misten waardoor er snel werd verondersteld dat de stof door de leerlingen werd begrepen.-Nee-Docenten waren deskundig en betrokken bij het overbrengen van hun kennis. Een uitzonderling daargelaten-Geen opmerkingen-Docenten vanuit eigen gelederen heeft de voorkeur, hierbij wel gelet op de didactische vaardigheden. Lekker veel voorbeelden uit de inspectiepraktijk.-Er is wel verschil tussen de mensen die van een opleidingsinstituut les geven en hoe de interne collega's trainingen verzorgen-[telcon Leo: Offering all knowledge as a part of the ETP may result in loss of knowledge before it is actually used by an inspector in practice. Some parts could be done e.g. prior to a project or an inspection where the knowledge is needed.]-Van de BOP-opleiding had ik alleen moeite met de manier waarop de docent van ATEX les gaf. Teveel details en foto's waardoor er minder overzicht was en dat in 2 dagen. Einde van de 2de dag liepen collega's weg toen de docent bleef doorgaan--Ja, laat ik het zo zeggen..... de kwaliteit van de docenten (met name didactiek) verschilde enorm. Kennis overbrengen is ook een vak-Nee, wel goed dat opleidingen vooral verzorgd worden door (senior) collega's, ervaringsdeskundigen dus.- Veel docenten zijn feitelijk ook collega's. Dat hoeft net fout te zijn maar praktijk leert dat zij weinig tijd hebben/krijgen om de opleiding beter op de doelgroep af te stemmen. Ook de externe docent van ATEX was deskundig maar kon het niet goed overbrengen. - Meeste waren goed. Zou wel meer aansluiting met onze praktijk en ervaring willen: Meer trainingen laten verzorgen tezamen met of door senior insdpecteurs.-Alle docenten zijn vakspecialisten, dat bleek uit hun passie en kennis. Alleen niet alle docenten hebben didactische vaardigheden. Daar zat nogal eens verschil tussen. -Werd op een prettige manier lesgegeven---Zoals al opgemerkt waren de docenten over het algemeen kundig en was hun stijl passend-Ervaren collega's spreekt mij altijd wel aan, als dat mogelijk is-----------------------------

Analyse vraag 13:
Ruim de helft antwoordt hier met "nee" of met de qualificatie "goed".
Diverse respondenten voegen positieve opmerkingen toe over mix van docenten, inbreng van praktijkervaring afwisselend met theorie, de waarde van discussies, interactieve aanpak en in groepjes werken.
Enkele respondenten noemen verschillen tussen docenten en gedrag van docenten uit het bedrijfsleven als minpunten.

vraag	non-respons	16

14-Werd er gecheckt of u de lesstof begrepen heeft ?

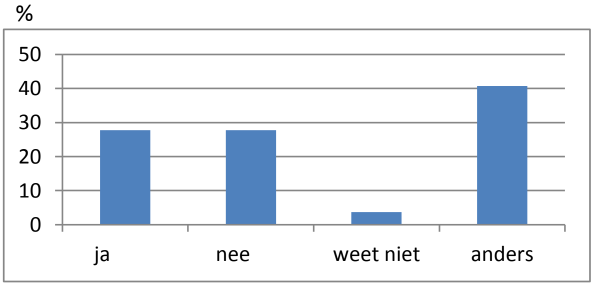

Begrijpcheck	aantal	procent
ja	7,5	28
nee	7,5	28
weet niet	1	4
anders	11	41
	27	100

Opmerkingen bij Anders:
-Daar was je vooral zelf verantwoordelijk voor: naar mijn mening was er voldoende ruimte voor het stellen van vragen zodat je dat in eigen hand had. Bij volwassen inspecteurs mag je dat verwachten lijkt me-Niet met een examen maar wel iedereen in de het uitwisselen van ervaringen en de discussie actief betrekken.-----Geen examens, maar die zouden niet veel hebben toegevoegd.-door sommige docenten wel, door anderen niet --Bij de meeste onderdelen wel-In de meeste gevallen wel d.m.v. opdrachten.----Ja en nee, er is minimaal toetsing van lesstof die intern wordt gevolgd. De externe opleidingen zoals NIM en BOA worden daarentegen wel getoetst.---ja voor BOP-opleiding en nee voor opleiding voor zittende inspecteurs---- deels ... De VBS elementen aan de hand van presentatie casus en ATEX (aan de hand van inspectie)- -Dat hing van de module af. Sommige modules werden afgesloten met presentatie of een terugkom dag, andere modules stopte gewoon.----Deels wel, deels niet--Enerzijds wel door klassikale presentaties. Dit geeft echter maar een marginaal beeld. Een controle hierop door meer met praktijkopdrachten te gaan werken zal mijns meer rendement geven.----------------------------

vraag	non-respons	13

15-Heeft u in praktijk bij uw werk gebruik gemaakt van wat u leerde in uw MHC opleiding?

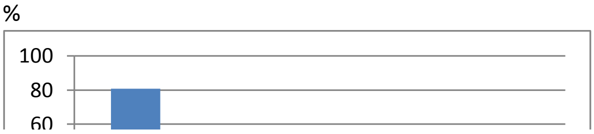

Praktijk gebruik	aantal	procent
ja	21	81
nee	0	0

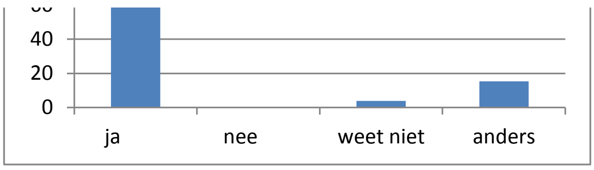

weet niet	1	4
anders	4	15
	26	100

[Anders: opmerkingen niet relevant]

vraag non-respons 16

16-Kunt u daar één of meer voorbeelden van geven ? (Gebruik in het werk)

Gebruiksvoorbeeld	aantal	procent
	24	

Als adviseur ondersteun ik MHC inspecteur bij vraagstukken over procesveiligheid, zoals bijvoorbeeld bijzondere situaties ten aanzien van explosieveiligheid, bezwaar- en beroepzaken waarbij risicobeoordeling en/of beveiligingsfilosofieën vaak centraal staan.-te veel om op te noemen denk ik -O.a. het speuren naar Ex-zone 0 -Ik heb een aantal malen mijn aantekeningen erbij gepakt om te kijken wat ik over een onderwerp had opgeschreven, onder andere KPI's, werkvergunningen/LMRA/TRA, veiligheidsstudies .-Inspecteren op ATEX, beoordeling van veiligheidsstudies en risicobeoordeling tijdens inspecties-Parate kennis tijdens inspecties bv ATEX, SIL, trails schrijven voor VBS elementen-Reactiekinetiek, faalkansberekeningen- Je maakte kennis met veel documenten met inhoudelijke 'Brzo-info'. Het Brzo- boekwerkje van Pieter was zeer praktisch, maar ook diverse normen die bedrijven gebruiken om aan het Brzo te voldoen, zoals de toenmalige CPR 20. (Nu PGS 6)
Hierbij merk ik op dat ik in die periode ook de KVK-opleiding deed en nu niet meer goed weet welke kennis ik gebruikte van de MHC- of HVK opleiding, of uit de praktijk (inspecties en ongevalsonderzoeken), terwijl ik ook al redelijk wat kennis had op veiligheids- en inspectiegebied.
-Bewust omgaan met welke instrumenten we in kunnen zetten om het gewenste effect te bereiken---Procesveiligheid, drukapparatuur en ATEX.-Beoordelen van Hazop's; voorbereiden inspecties-Er was nagenoeg geen relatie met de gedoceerde stof en het (moeten) toepassen daarvan. Het was aan de inspecteur of het geleerde toepaste-Inspectie van het Wbda 2016 (PED) en de ATEX richtlijn voor wat betreft nieuwe kennis- Neem de ATEX opleidingen, daar hebben de meeste wel (wat) ervaring mee uit de werkpraktijk. Ook hier blijkt dat uniform handelen zonder opleiding niet mogelijk is.
Maar ook de TIV (training interventievaardigheden) is een welkome opleiding, zeker vanuit de rol van toezichthouder. Het is toch anders wanneer je in je eigen bedrijf een boodschap hebt om iets voor elkaar te krijgen als wanneer je bij een "wildvreemd" bedrijf je interventietoolbox benut.

Sil/Lopa is een onderwerp van de HVK opleiding, feitelijk alleen maar snuffelen aan het onderwerp. Bij de interne opleidingen ervaarde ik meer praktische ervaring en meer diepgang.
--- In 2019 inspecteren we op drukapparatuur en hiervoor heb ik veel aan de opleiding gehad. Voor wat ik niet in de opleiding heb meegekregen kon ik in overleg met de senior inspecteur bespreken.
Bij inspectie over afwijkend gebied (ATEX) heb ik de opleidingsmap weer eens bekeken.
Voor inspectie op een VBS-element wat ik voor mij nieuw was heb ik de uitwerking van opdrachten in de BOP-opleiding nog eens bekeken ter inspiratie. Dit was voordat ik NIM/GIR-opleiding van BRZO+ had gevolgd.
--Natuurlijk heb ik de cursusmap nog weleens erbij gepakt. Dit n.a.v. een (vergelijkbare) situatie bij een bedrijf-PED, ATEX, Risicobeoordeling- ATEX inspectie, PED (voormalige zorgplicht na wijziging Wbda) , toetsen juistheid SIL verificatie bij inspectie- Niet specifiek. Te lang geleden.-Ja.
• Bij een bedrijf waren de werkinstructies voor de werkvloer alleen voorzien van tekst, moeilijke woorden en afkortingen. Ik heb daar een bevinding van gemaakt aan de hand van hetgeen ik geleerd heb in de module over taal.
• Het controleren van Hazops heb ik regelmatig mijn aantekeningen en de map teruggebladerd.
• Dat geldt ook voor ATEX. De map en de aantekeningen heb ik gebruikt om een generieke audittrail te maken die ik voor alle inspecties kan gebruiken.
-Op gebied van Atex (in kader opleiding een inspectie uit moeten voeren)-

--Inspectiemethodiek: Nim en GIR
Inspecteren van veiligheidsstudies
Het schrijven van boeterapporten
Kennis van explosieveiligheid omgezet in inspectietrails
Kennis van drukapparatuur omgezet in inspectietrails
Interventievaardigheden die ik in de praktijk heb gebruikt.
-Teveel om op te noemen en niet exact aanwijsbaar. Met name de specialistische onderwerpen zoals ATEX en drukapparatuur pas je direct toe in inspecties.-Toepassen aangedragen kennis inspecteren vbs element ii, ATEX, beoordelen instrumentele veiligheid (SIL) zijn een de voornaamste voorbeelden.-----------------------------

Analyse vraag 16:
Hier antwoordt circa 80% dat ze twee of meer dingen uit de MHC opleiding gebruiken.
De belangrijkste zijn: ATEX, PED, Risico studies en beoordeling. Minder vaak worden procesveiligheid, SIL/Lopa, KPI's, WVG, LMRA,TRA en HH instrumenten genoemd.
Enkele respondenten zien weinig relatie tussen aanbod en gebruik van kennis.

vraag non-respons 23

17-Zijn uw werk en de resultaten beter geworden dankzij uw MHC opleiding ?

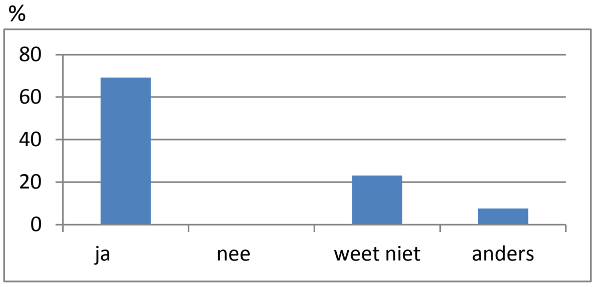

Werk verbetering	aantal	procent
ja	18	69
nee	0	0
weet niet	6	23
anders	2	8
	26	100

[Anders: opmerkingen niet relevant]

vraag non-respons 16

18-Kunt u daar één of meer voorbeelden van geven ? (Beter resultaat in het werk)

Verbetervoorbeeld	aantal	procent
	20	

Ik kom tot een beter onderbouwd advies, waarbij ik me meer zelfkritisch opstel.-zonder opleiding had ik het in de praktijk van de brzo partners moeten leren en uit mijn vorige opleidingen en ervaringen moeten putten. De opleiding heeft dus een meerwaarde-Door de opgedane kennis en ervaring uit de MHC (PVI)opleiding sta je sterker in je schoenen, heb je meer overredingskracht en hoef je niet telkens terug te vallen op het handhavingsregelement (of hoe dit ook mag heten) -Zonder MHC opleiding heb je weinig inspectie kennis denk ik-zie 16--Niet direct-Zie mijn opmerking bij vraag 16. Ik weet nu niet meer precies waar ik toen welke kennis heb opgedaan. Mijn ervaring is dat een opleiding, ook de MHC opleiding, je een duw in de goede richting geeft, maar dat je de echte diepgang leert in de praktijk. ----Mijn achtergrond is vanuit het bedrijfsleven. De technische kennis was al redelijk aanwezig maar ik heb deze met de opleiding aangevuld. Met name op het gebied van drukapparatuur en ATEX. Hierdoor hebben mijn inspecties meer inhoud gekregen.---De tijd zal het leren of mijn werk en resultaten beter zijn geworden dankzij de opleiding. Het betreft vaak een kwestie een inspectieonderwerp doen om het te laten beklijven en ervaring mee op te doen. Hetgeen uiteindelijk zal leiden tot een kwaliteitsverbetering-Zie ook vraag 16. Ik kan me eigenlijk niet voorstellen dat het aan opleiding zou ontbreken om op een uniforme en correcte wijze het inspectiewerk uit te voeren. Wanneer je vanuit het bedrijfsleven overstapt naar MHC geeft dit ook een andere kijk en werkmethode. ---Ik ben relatief nieuw bij MHC, dus bij elke inspectie heb ik voorbeelden hiervan--Ja, natuurlijk steek je altijd wel iets op. Voor mij geldt dat 'learning on the job' mij verder heeft gebracht. Als je tegen een specifiek probleem aanloopt ben je ook meer (intrinsiek) gemotiveerd om het op te lossen-Ik had al veel Brzo inspectie-ervaring toen ik bij MHC in dienst kwam, maar nog weinig kennis op het gebied van arbeidsveiligheid en specifieke ISZW onderwerpen zoals ATEX en PED. Veel van mijn huidige kennis op dat gebied heb ik tijdens de MHC opleiding opgedaan.- Op dit moment heb ik te weinig inspecties uitgevoerd voor MHC om dit concreet te constateren. Ik heb wel inzichten gekregen die mijn denken over de invulling van het VBS ietwat hebben gewijzigd ten opzichte van mijn verleden bij de omgevingsdienst- Niet specifiek, te lang geleden -Ik gebruik regelmatig mijn aantekeningen als geheugensteun. De lessen van de OMI hebben mij geholpen bij het opstellen van generieke audittrails per VBS element of inspectieonderwerp (ATEX, Scenario's).
Ik heb gelukkig al mijn aantekeningen digitaal gemaakt (Office Onenote) waardoor ik eenvoudig kan zoeken en knippen/plakken.
-In algemene zin, maar zeker op gebied van Atex (zie hierboven)---Meer kennis gekregen, zie mijn antwoorden bij vraag 16-Ook dit is lastig aan te geven, maar ik heb sterk het gevoel dat dit inderdaad het geval is geweest. Je weet collega's ook sneller te vinden na een dergelijke opleiding. Dit is ook zeker een meerwaarde!-Meer verdiepend en praktijk-/maatregelgericht, minder proceduregericht inspecteren-----------------------------

Analyse vraag 18:
De overgrote meerderheid van de respondenten antwoordt hier met "Ja" .
Als voorbeelden noemen ze dat ze sterker staan door meer kennis en dat de waarde daarvan in de praktijk moet blijken. De noodzaak voor een aantal van hen bestond er in dat typische SZW domein kennis (Arbo, ATEX,PED) bij hen ontbrak.

vraag non-respons 35

ALGEMENE VRAGEN

19-Heeft u opmerkingen over de kwaliteit van de MHC opleiding?

ETP kwaliteit	aantal	procent
	31	

De opleiding is van een hoog niveau en is divers. Onder Brzo toezichthouders steekt de MHC opleiding met kop en schouders uit in termen van kwaliteit en diepgang.-In het algemeen uitstekend -Van de laatste opleidingen weet ik te weinig-Nee, in die zin, je merkt dat het goed in elkaar zit, compliment -nee-die vond ik wel goed.-Het meeste was van een dermate abstract en academisch niveau wat in de inspectiepraktijk nauwelijks bruikbaar is-Ik heb geen tot zeer weinig informatie over de inhoud van de huidige MHC opleiding.-Mijn voorstel is om de BOA-opleiding niet direct bij aantreden van nieuwe inspecteurs te geven, maar dit later te doen, waardoor inspecteurs meer tijd hebben voor meeloopinspecties, waarbij ze de opgedane kennis in de praktijk toegepast kunnen zien worden- De kwaliteit van de opleiding is o.a. onderdeel geweest van KMS (en in dat kader regelmatig getoetst, in ieder geval de deelname aan de opleidingen).
We hebben altijd ingezet op zo'n 7 % van de beschikbare capaciteit voor opleidingen (in tijd). De opleidingen werden onderverdeeld in wetgeving / techniek / communicatie. Dus ook aandacht voor de manier waarop inspecteurs de boodschap brengen, hun houding (Training interventievaardigheden)
Ik denk dat we een kwalitatief goed stelsel hebben van opleidingen. Deze, aangevuld met de praktijktrainingen o.b.v. de praktijkbegeleiders, zorgen ervoor dat de MHC inspecteurs goed worden opgeleid.
-Vond het wel goed-Kwaliteit goed maar door de snelle opleiding geen of weinig mogelijkheden om de kennis in de praktijk te brengen door middel van het meelopen van inspecties. -Goede kwaliteit maar in 2019 te snel -Over het algemeen van grote kwaliteit, door materiedeskundigen-Deze zijn bij de voorafgaande vragen aan de orde gekomen- De kwaliteit kan worden vergroot door meer maatwerk te geven waarbij het theoretische deel wordt gekoppeld aan een thematische inspectiepraktijk. Zoals nu bijvoorbeeld gebeurd met drukapparatuur en ATEX. Een jaar lang met deze onderwerpen aan de slag met een goede bereikbare achterban brengt een inspecteur veel kennis.

Ondanks dat ik het niet prettig vind om regelmatig getoetst te worden zou de stof nog beter worden bestudeerd, mogelijk in voorbereiding op één of meerdere cursusdag(en), en collegiaal met elkaar besproken wanneer deze wordt getoetst. Deze toets zou bijvoorbeeld ook thuis kunnen worden afgelegd, het gaat om het verdiepingsmoment.

In voorbereiding van één of meerdere cursusdag(en) altijd goede informatie toesturen zodat inspecteurs vooraf zichzelf kunnen verdiepen in de geboden stof.

Het lesmateriaal meer in verhaalvorm beschikbaar stellen in de kennisbank. Daarbij kunenn verwijzingen zijn naar de IKC-kenniskaarten, BIM's etc.
-Ik zou denk ik meer mensen sneller in gaan zetten op de werkvloer door ze te laten meelopen met een ervaren inspecteur zodat ze sneller een beeld van het werkveld krijgen. Ik zou ook proberen het opleidingstraject wat compacter te krijgen, of meer te laten doen door zelfstudie of een ELO waar je vanuit huis aan kunt werken-

Ja: 1) het veranderende werkveld / ontwikkelingen in de procesindustrie (zoals bijv risico's die samenhangen met energietransitie en seculaire economie) meer verweven in de opleidingen, ook vaardigheden uitbreiden naar het crimineel/opsporingsgericht denken, maar ook aandacht besteden aan risico's die niet meteen op ons netvlies staan (de risico's van taal bijvoorbeeld ☐). . . . . .
.2) termen/eisen vaststellen didactische vaardigheden van docenten (seniors gaan nu naar een opleiding didactische vaardigheden)
.3) meer toetsen of de inhoud begrepen is
.4) meer efficiency; meer gebruik maken van andere lesvormen, bijvoorbeeld leren op afstand
-Het kennisniveau bij de MHC-opleiding is prima in orde. De wijze van lesgeven kan nog verbeterd worden om beter leereffect te bereiken. Ook na toepassing in de praktijk (b.v. na een half jaar of een jaar) zou per opleiding een evaluatie uitgevoerd kunnen worden over het effect van de opleiding. Wat weet men nog van de opleiding na een (half) jaar.-Naar mijn mening is de kwaliteit van de opleidingen redelijk tot goed-met name over de didactische vaardigheden van sommige docenten-I.h.a. wel redelijk goed.- Ik zou voor de nieuwe inspecteurs toch wat toets-elementen gaan inbouwen. Niet zo zeer toetsen op goed of fout maar meer in vorm van presentatie of opstellen audittrail waarbij het geleerde uit bijv. module 7 "procesinstallatie" verenigd worden module 4 (VBS elementen)- Ik denk dat (senior)inspecteurs meer moeten worden betrokken. Er voortdurend voortschrijdend inzicht in hoe te inspecteren, hoe een VBS er uit moet zien enz.. Dit kan dus alleen maar gegeven worden door zittende (senior) inspecteurs en niet door iemand die niet meer actief is in de praktijk.-Wat ik al eerder zei over de tussenpozen tussen de lessen kan wat mij betreft worden verlengt. Hierdoor heb je wat meer kans om het geleerde in de praktijk te brengen. Ik realiseer me dat de opleiding dan langer duurt, maar dat win je volgens mij op de lange termijn terug.
In de OMI heb ik ook de wetgeving gemist. Wat mij betreft mag er een module in komen over hét Brzo/Rrzo en over toezicht en handhaving. Ik merk nu in de praktijk dat het handhaven nog een blinde vlek is, zowel het stilleggen als het zoeken naar artikelen om je overtreding te schrijven. Daar had in de OMI meer aandacht aan besteed kunnen worden.
In de OMI ontbrak ook het onderwerp 'eigen veiligheid'. Af en toe komen wat voorbeelden voorbij maar echt een gedegen training in het herkennen van gevaren en het maken van een risicoafweging (op basis van de RI&E) ontbreekt naar mijn idee.
-Nee, is een prima opleiding.- In mijn geval de inspecteurs vragen.-Ik weet niet wat er op dit moment gedoceerd wordt, daarom heb ik geen mening-Inhoudelijk over het algemeen goed, sommige opleidingen waren voor mij te laat, door ervaring had inmiddels daarover al kennis opgedaan. Goede timing is belangrijk.
Zie ook mijn eerdere opmerking over inhoudelijke kennis van Brzo, dit vond ik echt niet goed.
-Opleiding die ik gevolgd heb (2005/2010) waren prima, voor zover ik me dit nog kan herinneren -Ik vind hem in de basis voldoende/goed. Alleen was de kennis die werd aangedragen in combinatie met de beschikbare tijd in sommige gevallen teveel. Hierdoor lag het tempo te hoog waardoor het rendement uiteindelijk achterbleef (bv theorie ATEX, procesveiligheid); te weinig tijd om de aangedragen kennis ook daadwerkelijk te laten landen (zoals gezegd voor sommige onderwerpen).
Ik denk overigens dat het eenvoudiger te behappen was vanwege mijn werkervaring binnen het werkveld.

Analyse vraag 19:
De opleiding wordt door de meerderheid van de respondenten als goed of zelfs uitstekend ervaren.
Ze geven diverse suggesties voor verbetering van de kwaliteit door bijv. eerder meelopen, lager tempo, meer e-learning thuis, opleiding meer koppelen aan inspectiethema's, veranderingen in de maatschappij en nieuwe risico's.

vraag non-respons 0

20-Welke Sterktes/Zwaktes/Kansen/Bedreigingen ziet u voor de MHC opleiding ?

ETP Sterktes	aantal	procent

Een gedegen en goed doordachte opleiding op basis van jarenlange ervaring-kleine groepen, veel ruimte voor discussie en vragen, veel kennis bij de docenten-Vooral kennis en ervaringen delen-Goede docenten, veel kennis-diepgaand en ter zake doend -veel kennis en informatie overdracht-Op het vlak van opleidingen lopen we mijlenver voor op onze collega's van het bevoegd gezag en de veiligheidsregio. Dat is een van de sterke punten van ons.-Zie antwoord 19.-zeer uitgebreid, alle relevante onderwerpen komen aan bod- de flexibiliteit van de inhoud van de opleidingen, de link met de inspectiepraktijk (eigen inspecteurs die als docent voor hun collega's optreden). Ook een sterk punt is dat de opleidingen worden gegeven aan inspecteurs van verschillende teams, hierdoor krijgen de mensen meer kennis van elkaars werkwijzen. Aan de opleidingen zit ook een hele sterke sociale component, je maakt contact, bespreekt casussen met elkaar, leert elkaar kennen.
In de OMI opleiding zit dan ook heel sterk die intercollegiale toetsing.
-kennis interne collega's-Docenten hebben veel kennis (kan ook een valkuil zijn).-Ervaren docenten-Mogelijkheid om medewerkers uniform op te leiden-Ik ben niet in staat om hierover een uitspraak te kunnen doen-Gedegen kennis-gedegen-bruikbaarheid/toepasbaarheid -kennis en ervaring van de docenten en sommige medecursisten---Professionele aanpak.- opleiding is passend voor nieuwe inspecteurs - allround, kritisch-iedereen leert hetzelfde, alle neuzen dezelfde kant op. Je leert elkaar goed kennen en je leert ook veel van elkaar.-Kennis docenten-Actieve leren en continu evalueren en innoveren--Inhoud, breedte--diepgang, praktijkgericht----------------------------

Analyse vraag 20: Sterktes
Respondenten geven in meerderheid aan het een gedegen, op kennis en ervaring gebaseerde, opleiding te vinden.
De belangrijkste redenen zijn kennis onderling delen, sociale interactie, groepjes, ruimte voor discussie en docenten met inspectie ervaring.

sub vraag non-respons		13

ETP Zwaktes	aantal	procent
	26	

Niet alle cursisten hebben het benodigde basiskennisniveau. Door het hoge niveau van de opleiding zullen niet alle cursisten even goed in staat zijn alle leerdoelen te bereiken.-te geconcentreerd in korte periodes-Het handhaven als het belangrijkst beschouwen -Er moet ook een bijscholing opgericht worden, kennis die je opdoet maar weinig gebruikt en niet onderhoud verdwijnt -soms wat te snel in 1 cursusdag -de gespecialiseerde modules werden door externen gegeven, hierdoor vaak geen link met handhaving. Handhaving module zo wie zo niet (voldoende) ingevuld.-(Te) veel tijd besteden aan onderwerpen en diepgang die nauwelijks toegevoegde waarde heeft voor de inspectiepraktijk.-Dat de nieuwe collega's in de eerste anderhalf jaar zeer veel theorie krijgen zonder tussendoor voldoende gelegenheid te krijgen om praktijkervaring op te doen om de theorie toe te kunnen passen.-onvoldoende tijd om de opgedane kennis toe te passen in de praktijk (nauwelijks tijd voor meeloopinspecties). - druk die het opleidingsstelsel legt op (vooral de nieuwe) inspecteurs. Ik hoor nog wel eens opmerkingen over de stress die de hoeveelheid opleidingen in het eerste jaar met zich meebrengt. Inmiddels zijn er ook opleidingen vanuit de inspectieraad die vooral zit op het kennis maken met andere inspecties.-veel kort op elkaar gepland-Teveel in te korte tijd zonder kennis in de praktijk te brengen.-Snelheid van het programma-Te vrijblijvend om he geleerde toe te passen. Geen toetsmoment--Door tijdgebrek een opleiding even tussendoor doen-duurt lang-het niet nagaan of de opleiding effectief is -komt het over en leert men er echt van, is het toepasbaar in praktijk---Veel informatie in relatief korte tijd, waardoor veel kennis weer wegzakt. Weinig aandacht voor herhaling voor zittende inspecteurs.- wordt niet getoetst- Zie bij 19: docenten die niet (meer) in de praktijk werkzaam zijn-tijdsperiode waarbinnen de modules worden gehouden. Samen met andere verplichte opleidingen ligt er veel druk op. Hierdoor krijgt huiswerk vaak geen prioriteit en gaan de deelnemers onvoorbereid naar de lessen.-Veel stof in korte tijd (niet alles blijft hangen)-Het inpassen tijdens het werk.--Timing opleiding in inwerktraject, Brzo kennis--te veel willen in te korte tijd----------------------------

Analyse vraag 20: Zwaktes
Als zwakte noemen de respondenten de grote hoeveelheid kennis die wordt aangereikt en die weinig met de praktijk in verband gebracht wordt. Wegzakken van kennis dreigt hierdoor.
Verder noemen enkele respondenten het ontbreken van evaluatie, een her- en bijscholingsprogramma en toetsing. Ook het ontbreken van handhaving als onderwerp en het verschil in deelnemer- startniveau worden genoemd.

sub vraag non-respons		16

ETP Kansen	aantal	procent
	27	

Er zit een hoop ervaring binnen de vakgroep MHC, na de werving van een groot aantal nieuwe inspecteurs in 2018. Deze ervaring zou benut kunnen worden om de kwaliteit van de opleiding te blijven garanderen.-Duidelijke MHC profilering (zie ook de bedreiging); relatie met zware ongevallen in de opleiding leggen zodat in inspecties daar de focus ligt. Werkelijk specialiseren, focus op MHC behouden-Een zelfverzekerde ervaren inspecteur het veld in sturen- Ga de opleiding ook geven aan bedrijven :). Scheelt wellicht handhaving. Ook inzoomen op de inspectieprocessen, zoals VR beoordeling
-meer praktijkgericht zodat niet alleen theorie maar ook het herkennen in het veld en hoe dan te handelen wordt behandeld -kennis binnen seniors en IKC beter benutten-Uitbreiden naar specifieke onderwerpen die direct raakvlakken hebben met procesveiligheid en arbeidsveiligheid.-Veel nieuwe mensen geeft de kans op nieuwe kennis.-realistischer tijdspad nemen voor het opleidingsprogramma-verdere ontwikkelingen van opleidingen ism het IKC (kenniscentrum van de inspectie). Van oudsher hebben we dit niet zo opgepakt, ik denk dat we daar kennis weg kunnen halen. Ook samenwerken met externe opleiders kan beter, om samen ontwikkelingen te signaleren en onder te brengen in opleidingen.
Er zijn ook kansen met andere vormen van onderwijs.
-beter plannen en afstemmen-De inspecteurs meer dan voldoende kennis geven over het inspecteren van BRZO.-Opleiding uitrekken naar 12 maanden ipv 6-De uniformiteit van het toezicht te verbeteren --Uniform werken en uniforme boodschap uitdragen-maak meer gebruik van digitale middelen-betere didactische vaardigheden docenten -periodiek refreshen van bepaalde onderwerpen, interactief leren, leren van elkaar in praktijk i.p.v. in een zaaltje zitten--maatwerk aanbieden-Doseren van informatie op het goede tijdsstip; koppeling met thema-inspecties en inspectieprojecten. Betrekken van praktijkbegeleiders bij opleidingen zodat e.e.a. met elkaar in de pas loopt.- uniform basiskennis overbrengen. Mogelijk ook rollenspel/casussen invoeren - zie 19-Spreid de modules iets meer zodat meer tijd ontstaat voor meelopen en maken van huiswerkopdrachten.-Meer praktijdgerichte inspecties uitvoeren met terugkoppeling in les (Atex)-Nieuwe methodieken gebruiken en middelen zoals postcast, video, blog, etc.--Goede Brzo kennis, bijv met toetsen. Meer intervisie met nieuwe en ervaren inspecteurs--meer gebruik maken van praktijkopdrachten als bij module ATEX, samenwerken met ARBO ivm ongevallenonderzoek-----------------------------

Analyse vraag 20: Kansen

Als kansen geven de respondenten aan:

Diverse aanpassingen o.h.g.v. specialisatie, e-learning, herkennen van knelpunten in praktijk, maatwerk , timing van kennis aanbod, koppelen opleiding en thema inspecties, aandacht voor inspectie proces en VR beoordeling, her-en bijscholing, uniformiteit van opleiding en toezicht. De opleiding zou meer inbreng vanuit IKC en nieuwe inspecteurs kunnen bevatten.

sub vraag	non-respons		13
	ETP Bedreigingen	aantal	procent
		23	

Veranderingen binnen de procesindustrie, wanneer deze niet goed worden opgepakt in de opleiding. -hang naar samenvoeging met Arbo waardoor Brzo specifieke onderwerpen onder dreigen te sneeuwen -Een onzeker onervaren inspecteur die nog maar een (red)middel heeft-als we worden opgeslokt tot één Brzo-inspectiedienst -te veel theorie en te weinig inspectiepractijk waardoor de theorie weer snel wegzinkt-door externen kans op belangenverstrengeling als deze ook BRZO adviseren-Het kennisniveau op procesveiligheidsgebied van nieuwe inspecteurs is erg gevarieerd. Het is lastig om een goed instapniveau voor de opleidingen te bepalen-Door de STIP wordt nu veel bepaald door collega's zonder Brzo/MHC achtergrond. Krijgen de nieuwe mensen nog wel de juiste onderwerpen in de MHC opleiding? -te veel in korte tijd, waardoor kans op gedemotiveerde inspecteurs (komen onvoldoende toe aan de inspectiepraktijk)-in het samenwerkingsverband BRZO+ wordt gekeken om opleidingen samen te voegen. De bedreiging zit dan op de diepgang van de opleidingen, als het opleidingsprogramma voor inspecteurs van verschillende diensten geschikt wordt gemaakt. --Te snelle inzet van inspecteurs in opleiding (versnelde opleiding).--Dat er voldoende tijd voor opleidingen beschikbaar is/blijft--Ontbreken van een actueel lesaanbod dat aansluit bij de inspectiepraktijk-kostbaar traject-we zijn teveel gedreven door oude kennis(overdracht) -iedereen gaat na opleiding zijn eigen weg en mogelijk eigen invulling geven van het geleerde---Werkdruk, tijdbelasting. Nieuwe inspecteurs zijn de eerste periode van hun diensttijd teveel met opleidingen bezig en hebben te weinig tijd om mee te gaan met collega's op inspectie. Balans tussen theorie en praktijk kan beter.- omdat deze niet getoetst wordt en, zeker bij nieuwe inspecteurs, veel tegelijk wordt georganiseerd, wordt de opleiding gevolgd zonder vooraf goed in te lezen. Hierdoor blijft veel opgedane kennis niet hangen - Geen-?-Kan geen bedreiging noemen-Belang opleiding m.a.w. tijd voor reserveren--Zou ik zo niet weten-------------------------------

Analyse vraag 20: Bedreigingen

De respondenten stellen hier de juiste respons op veranderingen in de procesindustrie centraal. Ze benadrukken de balans tussen theorie en praktijk en het op de juiste manier vaststellen van de inhoud van de MHC opleiding. Enkelen noemen tijd, hoge kosten, geen toetsing en belangenverstrengeling als bedreiging.

sub vraag	non-respons		26
vraag	non-response	gemiddeld	17

21-Hoe zou de MHC opleiding zich volgens u verder moeten ontwikkelen ?

ETP toekomst	aantal	procent
	30	

Nieuwe technologieën zouden een duidelijke plaats moeten gaan innemen, als ook cognitie, organisatiewetenschap en human error. Met nieuwe technologie gaat het om sensoring, datainterpretatietechnieken, verandering van de man-machine relatie, groeiende afstand tussen mens en proces t.g.v. technologische ontwikkeling en het belang van training van operators en ergonomie.-Zie 20-Doorgaan met uitwisselen van ervaringen en kennis. Maar ook vooral ruimte maken voor discussie.-Bijscholing opstarten, of evt ervaren inspecteurs laten aansluiten bij de opleiding zodat zij zowel zelf wat kunnen leren als ervaring delen.-Goede balans theorie- practijk-interactieve opdrachten. kennis binnen seniors en IKC beter benutten eventueel in combinatie met specialistische kennis van externen, vb ATEX opleiding door externe icm Peter Klein.
-Meer richting procesveiligheid en dan op praktisch gebied. Niet op gedetailleerd rekenniveau met 20 cijfers achter de komma. Maar hoe werken regelkringen, hoe zit een procesinstallatie in elkaar, hoe werkt dat nu met een scrubber.-Zie bij antwoord 20, bij kansen en bedreigingen-Basiskennis in stand houden en periodiek checken of ontwikkelingen mbt wet/regelgeving/etc aanleiding geven tot noodzaak aanpassingen van opleidingstraject- Wat is de MHC opleiding? Bedoel je alle onderdelen? Volgens mij doorgaan op de weg die we zijn ingeslagen. Verbreden naar de gebieden van inspectieSZW.-Interne collega’s uitgebreid met kennis specialisten-Meer tijd geven aan de opleiding waar het nodig is. Tevens meer opleiding in de richting toezicht & handhaving.-Meer afstemming tussen praktijkbegeleider (praktisch inspecteren) en theorie-Niet alle inspecteurs voor alles opleiden. Beter kijken hoeveel kennis, waar nodig is.-Anticiperen op mogelijke ontwikkelingen op het vakgebied, waardoor je een professionele gesprekspartner blijft voor de bedrijven-Structureel aandacht voor opleidingen. Daar waar geleerd kan worden via elektronische weg hiervan gebruik maken. Kennis is de kunst van herhalen.-Zoals eerder genoemd zou ik het traject compacter maken, introductiemodules hoeven niet zo uitgebreid-De MHC-opleiding is specifiek en aanvullend op basisopleiding (aanname-eisen), HVK of MOSHE en andere interne opleidingen. Ik zou de onder 19 vermelde punten meer willen betrekken bij de doorontwikkeling van de opleiding.-PDCA van de opleiding (met name check en act), interactief leren en leren van elkaar in praktijk-Deze zou zich naast het “bijblijven” ook moeten richten op het vergroten van de detail kennis op specifieke gebieden. Dit om een goede gesprekspartner te zijn voor de specialisten aan de andere kant van de tafel.-meer maatwerk aanbieden-Doseren van opleidingen, koppeling leggen met inspectieprojecten en thema-inspecties. Borgen dat kennisniveau van inspecteurs op peil blijft door een (herhalings) opleidingsprogramma voor zittende inspecteurs te ontwikkelen.- Basisopleiding combineren met e-learning modules (zoals bijv de e-learning taal bij brzo bedrijven). Praktijkopdrachten kunnen ook helpen om de stof nog meer en sneller eigen te maken- Ik denk dat het goed is om de opleiding zo te ontwikkelen dat er ook een mogelijk bestaat tot certificering van opleidingen of onderdelen. Dit in het kader van de titel hoger veiligheidskundige. Dat deze actueel blijft en je jezelf kunt laten certificeren. Verder dus duidelijk meer vanuit onze praktijk en senior inspecteurs erbij betrekken.
-Meer tijd tussen de module-lessen en extra modules over wetten, toezicht en handhaving en eigen veiligheid.-Vooral zo doorgaan is mijn mening.-Integreren wat er ook in het bedrijfsleven speelt.--Theorie en inspectieervaring op elkaar afstemmen: Eerst Theorie, kort daarna werkervaring. Dit werd goed gedaan met bijvoorbeeld de ATEX opleiding.-Opleiding moet meer gaan leven bij de inspecteurs en steeds meer de aansluiting zoeken bij actuele veiligheidsthema’s die spelen bij de chemische industrie.-Meer gebruik maken van praktijkgerichte opleiding. Bijvoorbeeld door middel van praktijkopdrachten en het klassikaal bespreken hiervan. Hierbinnen ook meer aandacht voor ongevallenonderzoek uitvoeren (praktijkopdracht); dit vindt nu wel plaats maar op basis van reeds bekende incidenten. Incidenten komen gelukkig meer sporadisch voor binnen MHC maar kansen liggen er mijns inziens in de samenwerking met ARBO.-----------------------------

Analyse vraag 21:
Respondenten willen de kwaliteit behouden en wijzen op de balans tussen theorie en praktijk en op evaluatie van de inhoud. Ze benadrukken her-en bijscholing, bijvoorbeeld via e-learning, om bij te blijven met veranderingen. Procestechniek, nieuwe technologie, Handhaving, andere SZW terreinen wordt genoemd.

vraag non-respons	3

22-Welke opmerkingen heeft u naar aanleiding van deze enquête ?

Opm. bij enquête	aantal	procent
	28	

Geen.-Heel goed initiatief dat hopelijk bijdraagt aan verdere consolidering en verbetering van Brzo inspecties door MHC -BRZO is niet zwart wit. B.v. deze stelling “Tagging van apparatuur en appendages bevorderd de kans op een zwaar ongeval” -Ik mis de waardebon die verloot wordt;) -geen-Succes met je onderzoek Paul-Ik zag pas dat het a-deel voor managers was toen ik al wat vragen had ingevuld. Er zaten wel vragen tussen waar ik ook wel een antwoord op had. Ik mis verder het onderzoek wat ik zelf gedaan heb naar het uitvoeren van de Brzo-inspecties in het kader van mijn MoSHE-opleiding. Daar wordt ook aandacht besteed aan de opleidingen van de inspecteurs van de verschillende diensten. Veel succes met het onderzoek en het schrijven van het artikel.-Goed dat nu alle MHC collega’s gevraagd worden.-Positief is dat bij eindgebruikers van de opleiding geëvalueerd wordt en dan verbeteringsvoorstellen opgepakt kunnen worden. -sorry te laat gereageerd- --Geen.-Geen--Geen opmerkingen-Enquête vergt toch wel meer tijd dan 10 a 15 minuten…… tevens schrik ik nu dat de vragenlijst voor 15 november 2019 retour moet terwijl ik de vraag pas 11 december 2019 heb ontvangen. Ik ga ervan uit dat mijn enquête toch wordt behandeld.-Aangezien ik sinds juni 2019 in dienst ben heb ik nog niet een volledig beeld van de opleiding zodat ik hier een beperkt oordeel over kan geven-maar zou er nog graag een interview over willen hebben-Wat ik gemist heb (en nog steeds zoekende ben), is een opleiding die dieper op het deel handhaving gaat. Wanneer bepaal je welk handhavingsinstrument wordt toegepast? Hoe schrijf je een overtreding en een eis (juridisch standhoudend)?
Valt NIM/GIR-opleiding van BRZO+ en de HVK-opleiding ook onder dit onderzoek?
-geen--Goed dat dit onderzocht wordt.- Positief omdat de inspectie zich nu zelf ook de spiegel voorhoudt- -Veel vragen leiden (in mijn geval) tot hetzelfde antwoord. Dat is dubbelop.-Geen-Uitstekend en benieuwd naar de eindconclusies.--Ik ben benieuwd of de uitkomsten daadwerkelijk meegenomen worden om de kwaliteit van de opleiding te verbeteren.-Prima, ik heb per ongeluk het managementdeel ook ingevuld, maar heb ik laten staan.-Geen-----------------------------

Analyse vraag 22:
De overgrote meerderheid heeft geen opmerkingen over de enquete en staat positief tegenover het evalueren van de opleiding.

vraag non-respons	10

I ISSUE Analyse GROF (voor de eerste 27 reacties)
Reacties per vraag zijn eerst kort samengevat. Daaruit is een aantal clusters van aanverwante inhoud afgeleid.

Alle reacties zijn daarna gekoppeld aan de gevonden 11 clusters, verder als ISSUES GROF aangeduid.
Voor de eerste 27 respondenten is geteld hoe vaak die voorkwamen. Deze analyse is toegepast op de vragen 5,8,9,11,12,13,16,18,19,20,21 en 22, en levert een frequentie verdeling GROF op.

	ISSUES GROF	aantal	GROF procent
A	1 Kwaliteit - inhoud, vorm, docenten, borging	20	13
C	2 Deskundigheid en vaardigheid van de inspecteur	16	11
F	3 Toetsing	14	9
J	4 Ontwikkelen van de opleiding	4	3
G	5 Koppelen opleiding en praktijk toepassing	8	5
B	6 Hoog tempo en meelopen in praktijk	19	13
E	7 Balans theorie en praktijk	13	9
H	8 Evalueren van de opleiding	8	5
I	9 Her- en bijscholing	5	3
D	10 Aandacht voor specifieke onderwerpen	15	10
K	11 Overige	27	18
		0	0
	Totaal	149	100

	ISSUES GROF TIMEPERIOD 1 AND 2 ONLY	aantal	GROF procent
A	1 Kwaliteit - inhoud, vorm, docenten, borging	4	12
C	2 Deskundigheid en vaardigheid van de inspecteur	4	12
F	3 Toetsing	5	15
J	4 Ontwikkelen van de opleiding	3	9
G	5 Koppelen opleiding en praktijk toepassing	2	6
B	6 Hoog tempo en meelopen in praktijk	2	6
E	7 Balans theorie en praktijk	2	6
H	8 Evalueren van de opleiding	2	6
I	9 Her- en bijscholing	0	0
D	10 Aandacht voor specifieke onderwerpen	4	12
K	11 Overige	5	15
	Totaal	33	100

	ISSUES GROF MANAGEMENT/STAFF ONLY	aantal	GROF procent
A	1 Kwaliteit - inhoud, vorm, docenten, borging	6	11
C	2 Deskundigheid en vaardigheid van de inspecteur	7	13
F	3 Toetsing	7	13
J	4 Ontwikkelen van de opleiding	4	8
G	5 Koppelen opleiding en praktijk toepassing	4	8
B	6 Hoog tempo en meelopen in praktijk	4	8
E	7 Balans theorie en praktijk	2	4
H	8 Evalueren van de opleiding	4	8
I	9 Her- en bijscholing	2	4
D	10 Aandacht voor specifieke onderwerpen	5	9
K	11 Overige	8	15
	Totaal	53	100

		GROF Procent Totaal	GROF Procent Periode 1 en 2	GROF Procent Mgmt & Staff only
	VERGELIJKING en FREQUENCY RANKING GROF op Procent Totaal			
	ISSUES			
A	1 Kwaliteit - inhoud, vorm, docenten, borging	13	12	11
B	6 Hoog tempo en meelopen in praktijk	13	6	8
C	2 Deskundigheid en vaardigheid van de inspecteur	11	12	13
D	10 Aandacht voor specifieke onderwerpen	10	12	9
E	7 Balans theorie en praktijk	9	6	4
F	3 Toetsing	9	15	13
G	5 Koppelen opleiding en praktijk toepassing	5	6	8
H	8 Evalueren van de opleiding	5	6	8
I	9 Her- en bijscholing	3	0	4
J	4 Ontwikkelen van de opleiding	3	9	8
K	11 Overige	18	15	15
	Totaal	100	100	100

VERGELIJKING - GROF:
Seniority vergroot de zorg over bijblijven met de ontwikkelingen. Het gevoel van drukte in het opleidingsjaar is weggezakt bij langer zittende inspecteurs. Toetsing wordt door hen belangrijker gevonden. Aandacht voor verschillen tussen theorie en praktijk leeft minder bij het management dan bij nieuwe inspecteurs. Nieuwe inspecteurs zien wegzakken van kennis als bedreiging. Dit is o.a. een gevolg van het niet direct toepassen in praktijk van kennis opgedaan in de opleiding en veel kennis tegelijk aanbieden.

II ISSUE Analyse FIJN (analyse voor de eerste 30 reacties)
Een fijnmazigere analyse is mogelijk met verder opsplitsen van issues GROF in verschillende onderdelen.
Dit levert onderstaande reeks issues FIJN op. Door het aantal keer dat elk van de sub-issues voorkomt te tellen ontstaat een frequentie verdeling FIJN. Deze verschilt van de frequentie verdeling GROF onder meer door opsplitsen van reacties over meerdere sub-issues.

	**ISSUES Samenstelling en onderdeel frequenties FIJN**	aantal	sub aantal	procent
A	1 Kwaliteit - inhoud, vorm, docenten, borging	109		
	Kwaliteit van de docenten (didactisch goed en inhoudelijk ervaren)		28	7,7
	Kwaliteit borgen		40	11,0
	Inhoud van opleiding		23	6,4
	Vorm van de opleiding (discussie, groepjes, kennis delen en sociale interactie)		18	5,0
C	2 Deskundigheid en vaardigheid van de inspecteur	26		
	Veiligheid van de inspecteur		1	0,3
	Zelfvertrouwen inspecteur		2	0,6
	Uniformiteit		8	2,2
	Mijn werk gaat beter		6	1,7
	Kennis van de inspecteur		4	1,1
	Deskundigheid en vaardigheid van de inspecteur		5	1,4
F	3 Toetsing	15		
	Toetsing		15	4,1
J	4 Ontwikkelen van de opleiding	45		
	Enquete opmerkingen		3	0,8
	Ontwikkelen van de opleiding		36	9,9
	Actueel mbt proces industrie		2	0,6
	Nieuwe werkvormen en technieken		3	0,8
	Opleiding aan bedrijven geven		1	0,3
G	5 Koppelen opleiding en praktijk toepassing	21		
	Koppelen opleiding en praktijk toepassing		4	1,1

	Gebruik (ATEX, PED, Arbo, HH, risico analyse, KPI, WVG, LMRA,TRA, SIL/Lopa, Audittrail, HAZOP, PGS-6)		17	4,7
B	6 Hoog tempo en meelopen in praktijk	37		
	Hoog tempo		25	6,9
	Meelopen in praktijk		12	3,3
E	7 Balans theorie en praktijk	17		
	Balans theorie en praktijk		17	4,7
H	8 Evalueren van de opleiding	10		
	Evalueren van de opleiding (vorm, inhoud, actualiteit, kwaliteit, efficiency en effectiviteit)		10	2,8
I	9 Her- en bijscholing	15		
	Her- en bijscholing		15	4,1
D	10 Aandacht voor specifieke onderwerpen	14		
	Aandacht voor specifieke onderwerpen (VR beoordeling, HH, Arbo, nieuwe technologie, human factors en uniformiteit van toezicht)		8	2,2
	Nodig voor mijn werk (ATEX,PED,Arbo)		6	1,7
K	11 Overige	53		
	Reorganisatie MHC		2	0,6
	Belangenverstrengeling		1	0,3
	Kosten		2	0,6
	Geen opmerkingen		19	5,2
	Overige		16	4,4
	Weet niet		13	3,6
		362	362	100

ISSUE Frequentie verdeling FIJN

		FIJN aantal	FIJN Procent
A	1 Kwaliteit - inhoud, vorm, docenten, borging	115	30
C	2 Deskundigheid en vaardigheid van de inspecteur	27	7
F	3 Toetsing	15	4
J	4 Ontwikkelen van de opleiding	46	12
G	5 Koppelen opleiding en praktijk toepassing	23	6
B	6 Hoog tempo en meelopen in praktijk	39	10
E	7 Balans theorie en praktijk	17	4
H	8 Evalueren van de opleiding	10	3
I	9 Her- en bijscholing	15	4
D	10 Aandacht voor specifieke onderwerpen	17	4
K	11 Overige	54	14
	Totaal	378	100

VERGELIJKING Frequenties Issues GROF vs FIJN

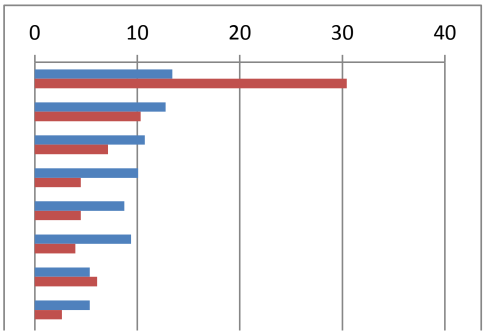

	ISSUES	GROF Procent	FIJN Procent
A	1 Kwaliteit - inhoud, vorm, docenten, borging	13	30
B	6 Hoog tempo en meelopen in praktijk	13	10
C	2 Deskundigheid en vaardigheid van de inspecteur	11	7
D	10 Aandacht voor specifieke onderwerpen	10	4
E	7 Balans theorie en praktijk	9	4
F	3 Toetsing	9	4
G	5 Koppelen opleiding en praktijk toepassing	5	6
H	8 Evalueren van de opleiding	5	3

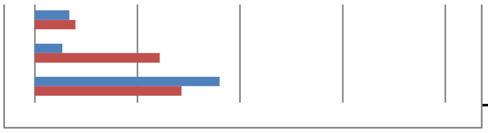

I	9 Her- en bijscholing	3	4
J	4 Ontwikkelen van de opleiding	3	12
K	11 Overige	18	14
	Totaal	100	100

SUB-ISSUES omschrijving met ranking naar frequentie FIJN — FIJN procent

(A) **1 Kwaliteit - borgen** — 11,0

Kwaliteitsborging via KMS
Voldoende leer- en oefenmateriaal
Nieuw geleerde kennis en opfrissen bestaande kennis
Kennis aanbod groot en breed
Docenten met inhoudelijke kennis nodig

(J) **4 Ontwikkeling van de opleiding** — 9,9

Meer aandacht nodig voor: actueel houden ogv BRZO werkveld, vakgebied, wetgeving, handhaving in praktijk, inspectie werkwijze, onderwijskundig, emerging technology, human factors, nieuwe risicos, SZW aspecten
Inspecteurs hebben verschillende startkennis en worden in de tijd steeds anders getraind door veranderingen
Effectiviteit verbeteren door Maatwerk (theorie aan inspectie thema in praktijk koppelen) en meer thuis uitvoeren van leer opdrachten (e-learning, projecten, etc)
Didactische kwaliteit en kennis overdracht
Meer diepgang in ATEX en PED opleidingen
Meer specialiseren op zware ongevallen
Meer inbreng vanuit IKC en door seniors bij de ontwikkeling
Lesmateriaal in verhaalvorm in kennisbank IKC opnemen
Opleiding openstellen voor bedrijven en certificeerbaar maken

(A) **1 Kwaliteit van de docenten (didactisch goed en inhoudelijk ervaren)** — 7,7

Goede mix van docenten nodig
Didactische vaardigheden nodig
Enthousiastme werkt beter
Te veel in detail gaan
Te theoretische of abstracte les stof
Senior inspecteurs zouden hier meer bij betrokken kunnen zijn
Docenten met actuele praktijk ervaring nodig
Kennis van instromende nieuwe inspecteurs delen
Kennis van IKC kan beter benut

(B) **6 Hoog tempo** — 6,9

Hoge tempo zorgt dat kennis niet altijd blijft hangen
Drukte met opleiding, werk en andere opleidingen
Hoog tempo vermindert kansen voor parallel oefenen in praktijk
Diepgang van opleiding lijdt onder hoog tempo

(A) **1 Kwaliteit - Inhoud van de opleiding** — 6,4

Meer behoefte aan praktijk ervaringskennis uitwisselen
Er is nog een gat in opleidingsniveau tussen de inspecteurs bij MHC en bij de andere bevoegde gezagen
Ervaringsdeskundigheid bij docenten wordt hoog aangeslagen
Het ingangsniveau van nieuwe inspecteurs is verschillend en daarmee een knelpunt
Meer trainingen door senior inspecteurs over actuele theorie en praktijk

(A) **1 Kwaliteit - Vorm van de opleiding** — 5,0

Elkaar als deelnemers leren kennen, waardevol contact
Elkaars kennis delen
Intercollegiale toetsing
In groepjes werken

Ruimte voor discussie
Interactieve praktijk opdrachten
Interactief leren
traject is omvangrijk, intensief
meer maatwerk
nieuwe technieken e-learning, blog,

(G) **5 Gebruik** 4,7
(ATEX, PED, Arbo, HH, WVG, LMRA,TRA, SIL/Lopa, Audittrail, HAZOP, PGS-6, Risico beoordeling, procesveiligheid, KPI's, Taal.)

€ **7 Balans theorie en praktijk** 4,7
Betere balans tussen aangeboden theorie en bruikbaarheid in praktijk nodig
Kennis aanbod te groot, te abstract, theoretisch, gedetailleerd, moeilijk op te nemen, slecht toepasbaar

(F) **3 Toetsing** 4,1
On the job door toepassing van opgedane kennis en vaardigheid
Afsluitende toets / examen
Interactief vaststellen
Aanwezigheid/deelname registreren
Examen opdracht doen

(I) **9 Her- en bijscholing** 4,1
Nodig vanwege bijblijven met BRZO werkveld, vakgebied, innovaties, herhaling van training, kennis op peil houden, verdiepen van kennis, delen van ervaringen.
Opleiding openstellen voor bedrijven en certificeerbaar maken

(B) **6 Meelopen in praktijk** 3,3
Meer meelopen in praktijk vergroot effectiviteit van aanreiken van theoretische kennis

(H) **8 Evalueren van de opleiding** 2,8
Evalueren van de opleiding is nodig ivm effectiviteit, innoveren, actualiseren, verbeteren.
(vorm, inhoud, actualiteit, kwaliteit, efficiency en effectiviteit)

[C) **2 Deskundigheid en vaardigheid van de inspecteur - Uniformiteit** 2,2
Uniformiteit in toezicht en handhaving
Onderling vervangen door inspecteurs

(D) **10 Aandacht voor specifieke onderwerpen** 2,2
Aandacht vergroten voor: PED, ATEX, aging, wetgeving (incl. BRZO), handhaving, VR beoordeling, Arbo aspecten, scenario's, nieuwe technologie, human factors en uniformiteit van toezicht.
