# Supplementary material for: Improving Education and Training of Dutch Major Hazard Control Inspectors: A 15 Years Longitudinal Case Study
Source: Int J Environ Res Public Health. 2020 Mar 17;17(6):1959. doi: 10.3390/ijerph17061959 (PMC7143160; doi:10.3390/ijerph17061959)
Supplement: Supplementary file 1 [file ijerph-17-01959-s001.zip › supplementary/Data file 4 External and internal triggers for change.pdf]

# Datafile 4 - External and internal triggers for change of the MHC ETP

| Nr | ETP Subject matter | Change 2003 – 2008 – 2014 – 2018 |
|---|---|---|
| 1 | Job Requirements | [HBO/Acad] [Same]<br>[HVK/Moshe] 2018 Swuste&Sillem Moshe evaluation |
| 2 | General legislation | PED, ATEX<br>AWB WAHHS |
| 3 | Special Legislation | BRZO'99 [ - - - - - ARIE - - - - - -] BRZO2015, RRZO2016 |
| 4 | Cooperation | LAT GIR RUD BRZO+ |
| 5 | How companies work | [same] |
| 6 | Safety Management System | BRZO'99 ISO 31000:2009 Seveso III,2012<br>Elements #1-7 Elements #a-h Elements #i-vii |
| 7 | Process installations | MOOIS Maintenance Bio-technology KPI<br>Software Architecture Ageing |
| 8 | Dangerous chemicals | Fuels quantity<br>GHS REACH CLP |
| 9 | Learning from accidents | [same] [new accidents] |
| 10 | Learning from practice | EC joint visits BES islands |
| 11 | Coaching | [Duo] [Accounts] [Peer support] |
| 12 | Safety Inspection Approach | AVRIM2 NIVRIM NIM NIM2 [LBR] |
| 13 | Reporting System | Bundled set Joint GIR report |
| 14 | Appraisal/Ranking | GIR ranking MHC Team reviews |
| 15 | Public Information | Joint public summary |
| 16 | Negotiations | CPR-20 /RIB NTA-8620, PGS-6/-15 |
| 17 | Law Enforcement | Small fines / Criminal law Large fines / Administrative law |
| 18 | Create Safety Knowledge | MHC Annual report LFA meetings Storybuilder Theses, Publications |
| 19 | Regulator organisation | Regional teams Directorate Programme structure |
| 21 | Professional standards | Uniform inspections Establish joint ICT/knowledge platform ISO-19011, 2018<br>Use SMS appraisal guidelines System vs Measures inspections/5 year cycle<br>Auditor role Risk based inspection<br>Cooperation between inspection partners<br>NIM inspection leader<br>Stronger law enforcement tools<br>Inspection must continue |
| 22 | ETP Change process | [same] [same] Less theory - more practice<br>Less company input – more SZW input<br>Restructured around SMS<br>Updated accident cases |
| 23 | Evaluation practice internal<br><br>external | 2003 Execution performance 2014 Exectution performance 2018 Execution performance<br>BTO,2007 2008 Hulst-Ouwerkerk- Criminal law enforcement weakness<br>KPMG,2003 2018 Wingerde et al-Administrative law enforcement burden<br>2012 Ale&Mertens 2017 contents/teaching style evaluation<br>2013 Lieshout 2015 Veld 2017 Ale et al 2018 Pronk et al<br>2013 contents evaluation 2017 Swuste& Reniers |
| 24 | Educational objectives | Less theory<br>Focus on risk assessment<br>Non standard conditions<br>Poor design and supervision<br>Use'what went wrong'<br>Less statistical theory<br>Include kpi, ageing, plant layout and software archit<br>Include culture, procedures and language issues<br>Companies to continuously assess risks<br>Less company input<br>Elaborate on risk management<br>Requirements & compliance<br>Learn from practice<br>Elaborate on process safety<br>Elaborate on toolbox |
| 25 | Quality of education | Adopt auditor style Structure along SMS elements<br>Reduce intro part Joint inspection partner training<br>Plan earlier after hiring<br>More interactive<br>Change teaching style and e-learning<br>Use info from inspection practice<br>Introduce examination |
